# Short Peptide-Based Smart Thixotropic Hydrogels †

**DOI:** 10.3390/gels8090569

**Published:** 2022-09-07

**Authors:** Bapan Pramanik

**Affiliations:** Department of Chemistry, Ben-Gurion University of the Negev, Beer-Sheva 84105, Israel; bapanpramanik92@gmail.com or pramanik@post.bgu.ac.il

**Keywords:** peptide, hydrogel, thixotropy, injectability, co-assembled hydrogel, composite hydrogel

## Abstract

Thixotropy is a fascinating feature present in many gel systems that has garnered a lot of attention in the medical field in recent decades. When shear stress is applied, the gel transforms into sol and immediately returns to its original state when resting. The thixotropic nature of the hydrogel has inspired scientists to entrap and release enzymes, therapeutics, and other substances inside the human body, where the gel acts as a drug reservoir and can sustainably release therapeutics. Furthermore, thixotropic hydrogels have been widely used in various therapeutic applications, including drug delivery, cornea regeneration and osteogenesis, to name a few. Because of their inherent biocompatibility and structural diversity, peptides are at the forefront of cutting-edge research in this context. This review will discuss the rational design and self-assembly of peptide-based thixotropic hydrogels with some representative examples, followed by their biomedical applications.

## 1. Introduction

**Scheme 1:**
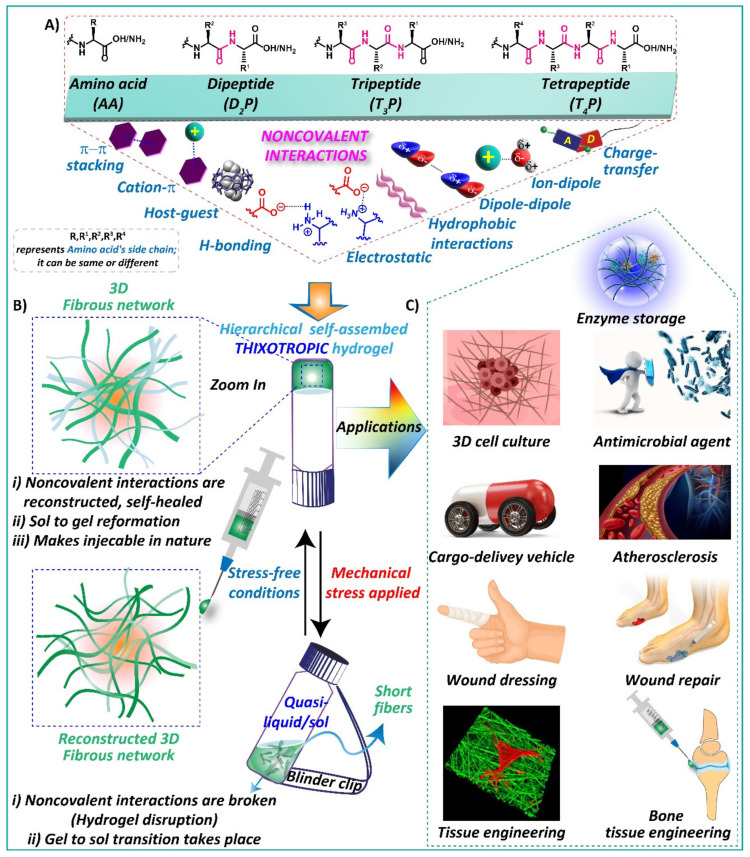
(**A**) Molecular structure and schematic representation of the involved noncovalent interactions to achieve hierarchical amino acid/short peptide-based self-assembled thixotropic hydrogel. (**B**) Schematic illustration of gel-sol-gel transition under applied mechanical stress and stress-free conditions, respectively, with representative microstructures. (**C**) Applications of thixotropic hydrogels. The pictures are adapted either from the internet or research articles. Enzyme storage [40]; 3D cell culture [41]. Both Refs. [40,41] are an open access article distributed under the Creative Commons Attribution License; antimicrobial agent [42]; cargo-delivery vehicle [43]; atherosclerosis [44]; wound dressing [45]; wound repair [46]. This article is licensed under a Creative Commons Attribution 4.0 International License; tissue engineering [47]; bone tissue engineering [48]. This article is licensed under a Creative Commons Attribution 4.0 International License.

Thixotropy is an amazing mechanical property observed in many gel systems where the gels break into a quasi-liquid or solution-like state under mechanical strain and recover their original form (here, gel) under static conditions (Scheme 1) [1,2,3,4,5,6]. In layman’s terms, thixotropy is a reversible, isothermal, time-dependent shear thinning behavior of gel systems distinguished by reversible transformation from gel-sol-gel structural transition [2,6,7,8,9,10]. Self-healing is another important rheological term recurrently connected to thixotropy and may be demarcated as the tendency of a gel system to eccentrically reorganize the bonding interactions after mechanical deformation [7,11]. The self-healing process can be visually established when separate pieces of gel connect to form an entity of hydrogel [4,5,12,13].

Thixotropic property can be examined with the help of different techniques. The most common classic procedure is the three intervals thixotropic test (3ITT), in which three zones were created in the whole cycle with “OFF–ON–OFF” modes [14,15,16]. In the first cycle (OFF state), a low strain is applied, i.e., within the linear viscoelastic region (LVR) [10,11] in which storage modulus (G′) is higher than loss modulus (G″) [11,13]. In the second cycle (ON state), a higher strain is applied, i.e., above the LVR region [10,11,17], and the change in dynamic mechanical moduli (G′ and G″) is monitored. G′ and G″ are the most critical rheological parameters and are generally employed to dictate the viscoelastic character of gels. G′ value is always greater than G″ value for an ideal gel system, but the inverse phenomenon is noticed when the gels undergo liquefaction [14,18]. In the last step of the cycle (OFF state), the same parameters are used as in the first cycle to restore the gel state. On this subject, the determination of gel strength recovery after the cessation of high strain is essential to know for their therapeutic applications, and that can be expressed in terms of the % Recovery parameter (% R) by employing the following equation [10]:

% R = (G_i_/G_a_) × 100 (1)

where G_i_ and G_a_ represent the storage modulus values of the gel before and after the disintegration of the gel network. At a lower strain, the gel strength recovery was found because of the reformation of the gel network by temporarily disturbed non-covalent interactions [10].

The hysteresis loop area test is another technique employed to measure the gel’s thixotropy in which the hysteresis area was formed by ramping up (structural break down) and ramping downward (recovery of deformed structure) protocol of shear rate [8,10].

In this context, self-assembling amino acids (AA), peptide-based (nature’s preferred building blocks) supramolecular hydrogels are at the forefront of advanced biomaterials owing to their fascinating properties such as exclusive biocompatibility, biodegradability, low toxicity, and bioactivity as well as their remarkable applications in biomedicine, including drug delivery, in vivo feedbacks in targeted tissue niches, regenerative medicine, etc. [19,20,21,22,23,24]. Hydrogels are three-dimensional (3D) cross-linked polymeric networks in which a substantial amount of water is entrapped and have been universally accepted biomaterials for tissue engineering applications [24,25,26,27,28,29]. The 3D fibrous networks of these hydrogels efficiently mimic the fibrous part of the extracellular matrix (ECM) protein architectures and are capable of sustaining cell differentiation and growth when applied as coatings or 3D matrices [30]. Among them, low molecular weight gelators (LMWGs) have attracted enormous attention in the field of medicine compared to other materials such as polymers, such as polymers, polypeptides [31,32], and nanocomposites, owing to their facile synthesis, high scalability, low-cost production, spontaneous self-assembly, stimuli responsiveness, injectability, etc. [9,19,23,27,33]. In comparison, most of other materials except for LMWGs rely on organic synthesis and polymerization. Tedious procedure and high-cost value of the chemical reactions and purification steps are other limitations that restrict their application in the biomedicine field [34]. Additionally, amino acid/peptide-based hydrogels have garnered great attention due to the fine-tuning ability of their gelation behavior and mechanical properties without disturbing their biocompatibility [19,35,36]. LMWGs usually form 3D fibrous networks through the self-assembly of the building blocks in which non-covalent interactions such as H-bonding, hydrophobic interactions, van der Walls forces, electrostatic interactions, and π-π stacking play a vital role (Scheme 1) [9,36,37,38]. The reversible breaking and restructuring of the self-assembled network mediated by the above-mentioned weak physical bonds offer thixotropic properties, i.e., rapid shear thinning and shear recovery (Scheme 1) [33,37,39]. Furthermore, the entire fabrication process for preparing these LMW supramolecular hydrogels is more straightforward and easier compared to chemically cross-linked polymeric materials, ideal for biomedical applications.

Only few of them are blessed with this unique thixotropic property, making them a fascinating class of dynamic self-assembled biomaterials and paving the way for application in various biological fields. It is still not well understood why some gels are thixotropic in nature but others are not. Unfortunately, there is no way to predict whether a hydrogelator will be thixotropic. In the same perspective, in 2017, Tomasini et al. published an outstanding review highlighting the thixotropic behaviors of peptide building blocks [33]. The review outlined some representative thixotropic hydrogels’ preparation and biological applications, particularly in cell culture and encapsulation. Thereafter, there has been a prolonged deficiency of comprehensive reviews on the usage of LMWGs as smart hydrogels endowed with thixotropic and injectable behavior, except for Tomasini’s one [33]. Therefore, there is still much room for exploring the thixotropic properties of peptides with more useful information and, ultimately, applications in the field of tissue engineering and regenerative medicine. In this review, the representative examples of thixotropic peptide hydrogelators are restricted up to tetrapeptides. The peptides, i.e., dipeptide, tripeptide, and tetrapeptide, were assigned D_2_P, T_3_P, and T_4_P, respectively, throughout the manuscript.

Under a high applied shear force, the fibrous network diminished because of the mechanical disruption of the physically weak non-covalent bonds associated with the network and exhibited a loop-sided curl structure [7,11]. As a result, the entrapped solvent molecules become free to flow, resulting in a gel-to-sol transition state [7,49]. However, reorganization of the fibrillar network entrapping solvent molecules was observed at a lower shear force. The recovery of the hydrogel network is highly dependent on shear rate, shear duration, and stiffness of the gels before shear. In addition, the thixotropic nature relies highly on the strength of interactions among the non-covalently cross-linked motifs. Depending on the recovery time of the non-covalent cross-links, these gels can be classified as strongly thixotropic (a considerable amount of time needed), moderate thixotropic and weakly thixotropic (rapid recover time) [28]. The thixotropic behavior of gels dictates the gels’ injectability nature, i.e., the rapid gel formation after being extruded through a high gauze syringe needle, which is indispensable for clinical operation [4,50,51]. These LMWGs usually showed storage moduli in the range of 10^2^–10^3^, which perfectly matches with native biological soft tissues, making them a good candidate for injectable hydrogels [51].

In particular, therapeutics/cells loaded with these novel peptide thixotropic gels can be utilized as a promising injectable drug delivery platform for local drug delivery because these gels can be directly injected at the site of interest through a syringe needle [25]. Injectable hydrogels are promising biomaterials in the field of regenerative medicines, including brain injury and wound dressing owing to their plentiful advantages such as easy incorporation of cargos/cells, easiness in implantation, in situ formability, and nominal invasiveness [25,51,52]. In addition, thixotropic hydrogels exhibited their importance compared to existing implantation methods for cell delivery applications, as they evade the plausible risks of cell loss as well as protect the cells from shear damage during injection and sustain the retrieval of the injected hydrogels [51].

This review presents the most recent examples (last five years) of LMWGs with thixotropic properties with an overview. The rational design and self-assembly of these LMWGs with thixotropic features are well focused. The detailed thixotropic rheological parameters of the representative amino acid, dipeptide, cyclic dipeptide, tripeptide, tetrapeptide, co-assembled, and composite hydrogels are displayed in tabular form. Finally, these thixotropic hydrogels’ biological applications, challenges, and future directions are thoroughly discussed. This is a much-improved review with much more helpful information for designing new thixotropic peptide molecules to apply in tissue engineering applications.

## 2. Amino Acid (AA)-Based Thixotropic Hydrogel

**Figure 1:**
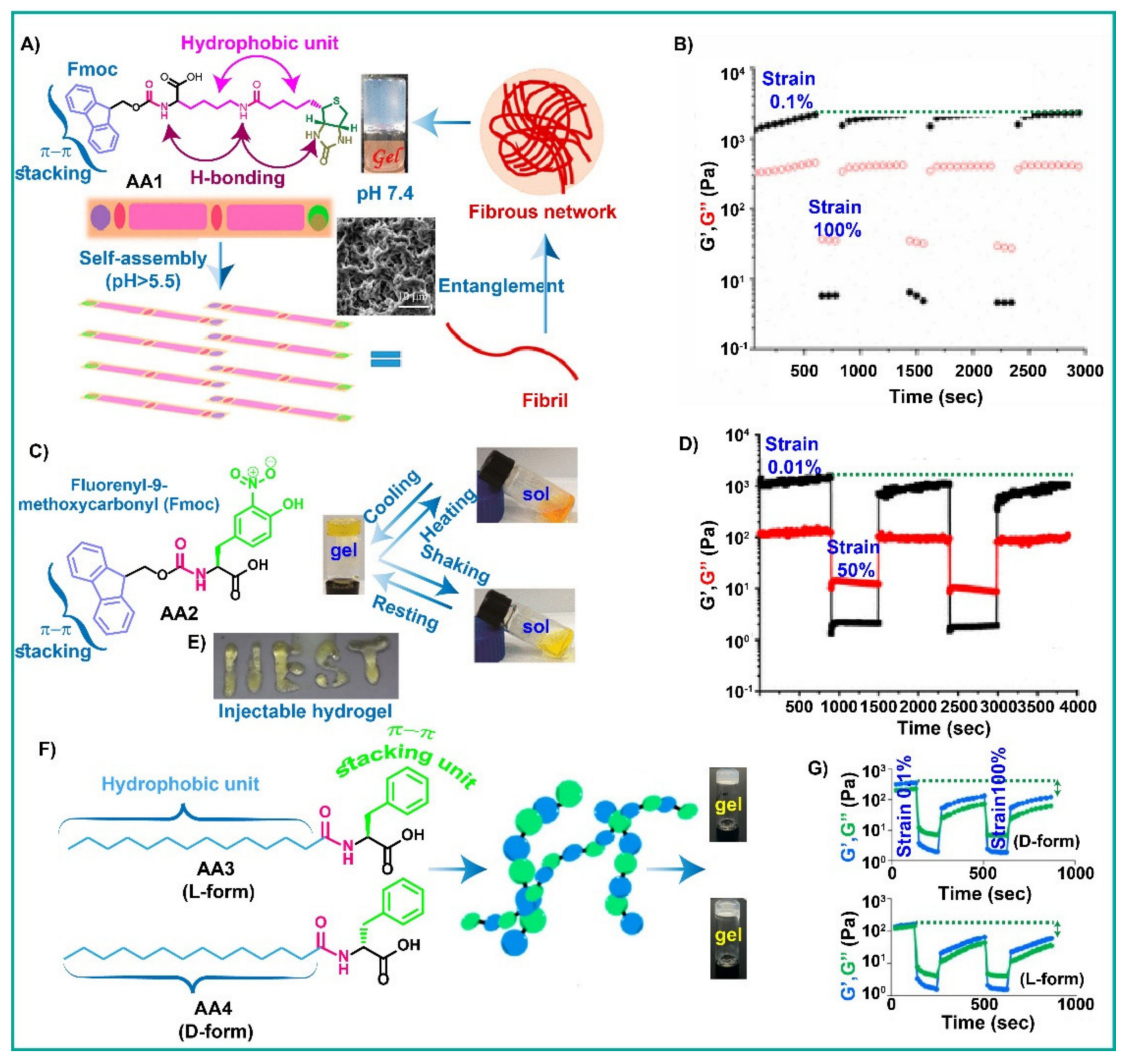
(**A**) Chemical structure of the hydrogelator (AA1) and possible self-assembly schematic. (**B**) Thixotropy test of AA1 hydrogel; reproduced with permission from Ref. [54]. Copyright (2018) American Chemical Society. (**C**) Chemical structure and stimuli-responsive behavior of the AA2 gelator. (**D**) Rheology experiment confirming the thixotropic behavior of AA2. (**E**) Injectability behavior of AA2; reprinted with permission from Ref. [61]. Copyright (2019) American Chemical Society. (**F**) Self-assembly and hydrogel formation of amino acids, AA3 and AA4. (**G**) Shear-recovery cycle of AA3 and AA4; reproduced with permission from Ref. [62]. Copyright (2019) MDPI.

In light of LMWGs, AA-based supramolecular gels are an ideal example [53]. Lysine (Lys) is an essential basic amino acid widely used to prepare LMW hydrogelators [3,54,55]. Lys plays a vital role in maintaining the hydrophilicity of the system, and side-chain free amine (–NH_2_) takes part in pH sensitivity [56,57].Taking these into consideration, in 2018, Shanmugam et al. reported biocytin based LMWG (AA1, Figure 1), made of 9-fluorenylmethyloxycarbonyl (Fmoc)-Lys and D-biotin, which undergoes self-assembly through H-bonding between NH and C=O of the peptide backbone, hydrophobic interactions between the amino acid backbones and π-π stacking intermolecular interactions between aromatic Fmoc moieties in a wide pH range (5.5–9.8) to form a supramolecular gel (Figure 1A, Table 1) with thixotropic behavior [54]. Biocytin is an amidation product of D-biotin and Lys, used as an intracellular marker and neuroanatomical tracer. Over the last several decades, Fmoc moiety has been widely used to promote peptide-based hydrogelators owing to their hydrophobic and π-π stacking interactions within fluorenyl rings, methoxycarbonyl-assisted steric optimization, and finally, the tendency of a carbonyl group for additional H-bonding [55,58,59,60]. Thixotropic behavior of the gel was evaluated by applying alternating low and high strain on it (Figure 1B). At a low magnitude of strain (γ = 0.1%), a characteristic feature of G′ > G″ was noted, indicating the viscoelastic nature of the gel. However, when the strain was enhanced (γ = 100%), the gel underwent liquefaction (G′ < G″). The liquid-like state recovered its gel state (G′ > G″) within a few minutes upon removing high strain, indicating the self-healing nature of the hydrogel (Table 1). The measurement was continued for another two cycles. The gel almost completely recovered its moduli value, confirming the excellent self-healing ability of the hydrogel (Figure 1B). The phenomenon can be attributed to the noncovalent aspects of interactions to promote self-assembly. Furthermore, Nanda et al. demonstrated the self-assembly behavior of modified tyrosine derivative, Fmoc-Tyr(3-NO_2_) (AA2, Figure 1C), which forms a self-supporting hydrogel in PBS (pH 4.5–8.0) [61]. 3-nitro tyrosine (3-NT) is well known as a marker of cell damage and inflammation and is found in biological fluids such as lung aspirates (bronchoalveolar lining fluid), plasma, and urine. The presence of the nitro group enhanced the gelation ability in a broad pH window. The hydrogel starts to flow under either the presence of heat or mechanical stress and reforms again under stress-free conditions. The gels formed at pH 5.5–8.5 displayed thixotropic behavior. Still, out of these prepared hydrogels, the gel at pH 7 showed the best thixotropic response with the excellent recovery of the moduli owing to its relatively lesser recovery time than other gels (Figure 1D, Table 1). The gel exhibited injectable properties, as shown in Figure 1E. In the same year, Kraatz et al. reported myristic acid (CH_3_(CH_2_)_12_COOH)-capped L- and D-Phenylalanine (Phe) (AA3 and AA4, Figure 1F), which are much more prone to form a hydrogel in a monobasic phosphate buffer at pH 7 [62]. Phe is a well-known aromatic hydrophobic amino acid that can readily undergo a self-assembly process within a network through hydrophobic and π-π interactions to form supramolecular gels [55,59,63,64,65,66,67]. Both gels showed thixotropic behavior, as confirmed by cyclic step-strain rheological measurement (Figure 1G, Table 1).

**Figure 2:**
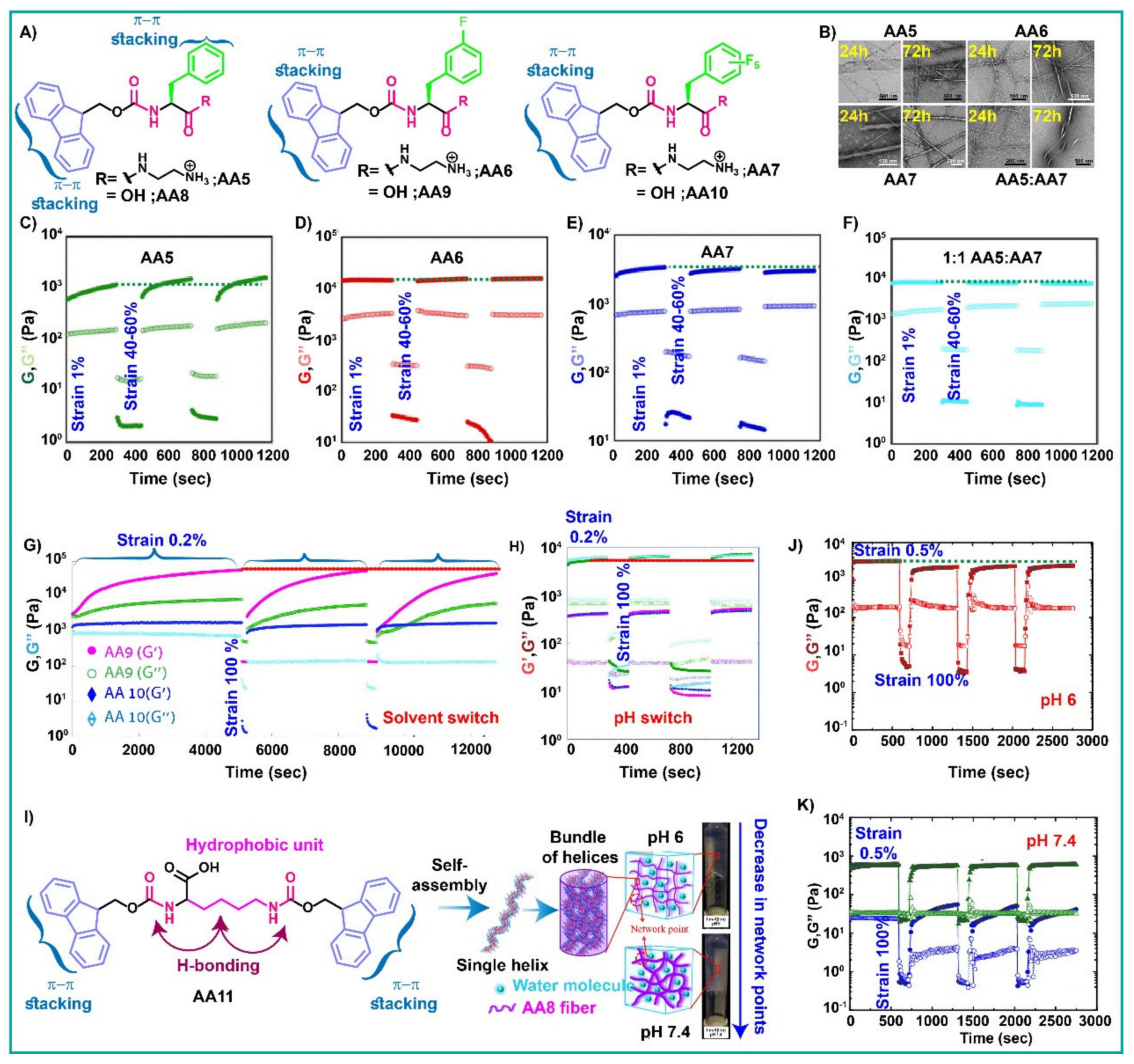
(**A**) Molecular structure of the amino acid hydrogelators (AA5–AA10); redrawn from Refs. [53,68]. Copyright issue is not applicable here. (**B**) TEM images of the representative pure and co-assembled hydrogelators. (**C**–**F**) Step-strain rheological test of the representative hydrogel; reproduced with permission from Ref. [53] Copyright (2019) American Chemical Society. (**G**,**H**) Thixotropy cycle of the amino acid gelators; reproduced with permission from Ref. [68]. Copyright (2020) Royal Society of Chemistry. (**I**) Schematic illustration of possible self-assembly and network formation of AA8 hydrogelator. (**J**,**K**) Rheology experiment confirming the thixotropic behavior of AA8 at pH 6 and 7.4, respectively. Reproduced with permission from Ref. [69]. Copyright (2020) American Chemical Society.

It is well documented in the literature that the presence of Phe in Fmoc-capped peptides facilitated synergism with Fmoc moiety through π-π stacking during the self-assembly process [68,70,71]. For example, Nilsson et al. engineered Fmoc-capped cationic derivatives of Phe (AA5, AA6 and AA7, Figure 2A) in which the C-terminal of the amino acid is protected with diamino propane (DAP) [53]. Halogen molecules are also introduced to form self-assembled self-supporting hydrogels with fibrillar architectures (Figure 2B). The presence of a halogen group (here fluorine (F)) amplified the self-assembly by perturbing intermolecular aromatic interactions. All these gels showed rapid and good gel recovery except for AA5, as shown in Figure 2C–F, where the gradual increment of G′ over one minute was observed to reform the gel network (Table 1). Despite being the weakest gel in the series, AA5 demonstrated better moduli recovery than the original moduli, which can be attributed to the more stable gel formation following the first deformation. In a similar context, the same group anticipated the emergent thixotropic properties of Fmoc-capped Phe derivatives (AA8, AA9, and AA10, Figure 2A) [68]. The derivatives undergo self-assembly to form a supramolecular gel through two comparative gelation conditions: solvent switch and gradual pH adjustment. The thixotropy property of the solvent switch-induced gels was measured with the help of dynamic time sweep rheological experiments (Figure 2G). At a low magnitude of strain (γ = 0.2%) for 85 min, both AA9 and AA10 exhibited a higher G′ value compared to G″, indicating the viscoelastic nature of the hydrogel (Table 1). G′ was found to be significantly higher in the case of AA9. The inversion of the moduli value was observed (G′ < G″) at a higher strain (γ = 100%) for 2.5 min, owing to the disruption of the gel network, implying a quasi-liquid state. However the gel recovered its structure (G′ > G″) when again the strain was decreased to its initial value (γ = 0.1%) with enough time of 1 h. In the case of AA10, the gap value between G′ and G″ was enhanced more during the recovery window than in the initial formation. This may be attributed to a more stable gel network after the first deformation. On average, the gel regained 86% of its initial moduli value, showing delayed self-healing shear recovery behavior across longer recovery times. Next, the thixotropic properties of pH-induced gels were evaluated with the alternating cyclic strain experiment in which a low strain (γ = 0.2%) was applied for five minutes, followed by a high strain (γ = 100%) for 2.5 min, and then again low strain (γ = 0.2%) for five minutes and continued for the rest of the cycle (Figure 2H). The gel exhibited a viscoelastic nature at a lower magnitude strain (G′ > G″). However, when the strain was increased, the moduli values were inverted (G′ < G″) owing to gel network destruction to reach the quasi-liquid state. However, when the strain was removed to its initial value, the reconstruction of the gel network was observed immediately, confirming excellent self-healing ability.

In advanced studies, Shanmugam’s group already established that the presence of an additional Fmoc group (AA11) to prepare the hydrogelator of twin Fmoc-capped L-Lys induced thixotropicity in the hydrogel [9]. Later, in 2020, Deshpande’s group, in collaboration with Shanmugam’s group, reported the same Fmoc-functionalized L-Lys, which forms a supramolecular gel at pH 6 and 7.4 with a three-dimensional fibrillar network (Figure 2I) [69]. The gels are thixotropic in nature and exhibited a slow improvement in recovery percentage from 73.9 to 76.5, while the deformation percentage decreased from 26.1 to 23.6 (Figure 2J, Table 1). In contrast, the gel at pH 7.4 showed poor recovery of moduli at the low strain stage, followed by the deformation step (Figure 2K). The deformation percentage value upgraded from 90.4 to 91.3 and 92.8%, whereas the recovery percentage value kept falling from 9.6 to 8.7 and 7.2%. The recovery rate can be improved when the high strain value is fixed at 20%. The opposite trend was observed for recovery (92.8 to 95.5 and 99.94%) and deformation percentage (7.2 to 4.5 and 0.06%), confirming the transient nature of the gels.

Based on the reported amino acid-based thixotropic hydrogels, Fmoc-Lys-Fomc showed good recovery (>92%) at the end of four cycles. Still, the toxicity of the Fmoc-group limits their practical applications in the biomedicine field. It is documented that the presence of the Fmoc unit makes the hydrogelators necrotic to some human cell lines over prolonged incubation times owing to the Fmoc-degraded byproducts [58]. In addition, the chemical instability of the carbamate group at higher pH causes partial deprotection during pH-switched gel formation. Therefore, aliphatic hydrocarbon-capped Phe hydrogelators are preferred for biological applications.

## 3. Dipeptide (D_2_P)-Based Thixotropic Hydrogel

**Figure 3:**
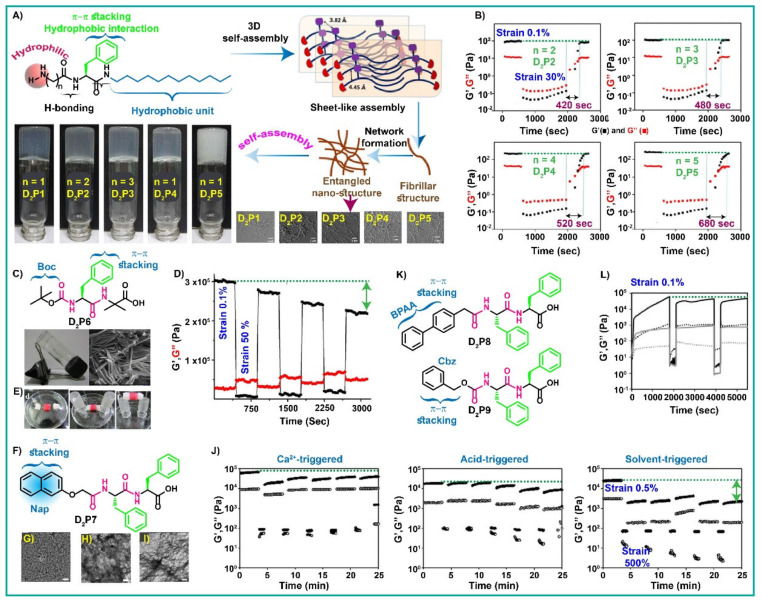
(**A**) Molecular structure and self-assembly of the dipeptide hydrogelators (D_2_P1-D_2_P5), FE-SEM images and vial containing hydrogel photographs. (**B**) Rheological step-strain measurements of the respective dipeptide hydrogels; reproduced with permission from Ref. [72]. Copyright (2017) American Chemical Society. (**C**) Chemical structure, gelation studies and SEM image of the dipeptide (D_2_P6) xerogel. (**D**) Thixotropy test of the D_2_P6 hydrogel. (**E**) Macroscopic self-healing ability of D_2_P6; reproduced with permission from Ref. [73]. Copyright (2018) American Chemical Society. (**F**) Molecular structure. (**G**–**I**) TEM image. (**J**) Recovery test of the dipeptide, D_2_P7 at different experimental conditions; reproduced with permission from Ref. [74]. Copyright (2017) Royal Society of Chemistry. (**K**) Chemical structure. (**L**) Thixotropic analysis of the dipeptide hydrogelators (D_2_P8 and D_2_P9); reproduced with permission from Ref. [75]. Copyright (2018) American Chemical Society.

Inspired by the Phe properties (discussed in the amino acid-based thixotropic hydrogel section), in 2017, Banerjee et al. reported a series of dipeptides in which the C-terminal of the amino acid is coupled with dodecyl (C_12_) amine, whereas the N-terminal is protected with different chain lengths (1: glycine, D_2_P1; 2: β-alanine, D_2_P2; 3: 4-aminobutyric acid, D_2_P3; 4: 5-aminovaleric acid, D_2_P4; and 5: 6-aminocaproic acid, D_2_P5) free amine (–NH_2_) [72]. The peptides experience self-assembly in ultrapure water at pH 6.6 with a nanofibrillar network (Table 2). All these peptides form self-supporting thixotropic hydrogels (transparent in nature for D_2_P1-D_2_P4, and translucent for D_2_P5, Figure 3A) except for D_2_P1 with intertwined nanofibrillar architectures. The gel recovery time after the first cessation of high magnitude strain is highly dependent on the chain length of N-terminal residue and follows the order D_2_P5 (680 sec) > D_2_P4 (520 sec) > D_2_P3 (480 sec) > D_2_P2 (420 sec), confirming high mechanical strength of D_2_P5 (Figure 3B). This can be attributed to the profound van der Waals interactions within the D_2_P5 peptides in their gel state.

Based on the outstanding self-assembly ability of Phe, Haldar et al. demonstrated a dipeptide (D_2_P6) composed of Phe and a non-natural amino acid, α-aminoisobutyric acid (Aib), which undergoes self-assembly into freshly prepared NaOH solution through non-covalent interactions to form a transparent hydrogel (Figure 3C) [73]. The gel is thixotropic in nature, as confirmed by the rheological loop test (Table 2). The gap between G′ and G″ is higher, indicating a stable gel network, but the gap continuously decreased after the disintegration of the gel network with the applied high strain (Figure 3D). Therefore, the gel failed to recover its initial moduli value, confirming moderate self-healing ability. The gel showed various self-supporting geometrical shapes, as shown in Figure 3E.

Diphenylalanine (PhePhe) is the most used minimalistic dipeptide motif ever for the creation of different self-assembled nanoarchitectures by peptide chemists as well as by material chemists and biologists [5,57,88,89,90,91,92,93,94,95]. The noncovalent interactions such as H-bonding, hydrophobic, and π-π stacking interactions between PhePhe building blocks are primarily responsible for offering well-defined nanoarchitecture such as nanofibrils, nanotubes, nanowires, nanospheres, etc. [88,89,90,96,97,98]. In addition, PhePhe-based peptides with a nanofibrous network are highly desirable owing to their functional similarity with natural extracellular matrices. Utilizing this self-assembly approach of PhePhe, in 2017, Adams et al. demonstrated the gelation behavior of a naphthalene (Nap)-capped dipeptide, D_2_P7 (Figure 3F), by employing three different ways such as metal (Ca^2+^), acid-, and solvent-triggered processes with thixotropic behavior [74]. To form the gels, (i) D_2_P7 was dissolved at high pH followed by the addition of Ca^2+^ salt (metal-induced); (ii) the reduction of pH after dissolution at high pH (acid triggered); and (iii) the dilution by water after dissolution at high concentration in water-miscible organic solvent (here DMSO, solvent-induced). Nap’s presence enhanced the gelator molecule’s self-assembly propensity through additional π-π stacking among the Nap units (Figure 3G–I). The rheological step-strain experiment indicated that the Ca^2+^-triggered gel recovered only 32% of its original G′ value after the first applied higher strain and an average of 50% after five cycles (Figure 3G, Table 2). In comparison, the acid-triggered gels almost completely recover their original G′ value after the first high magnitude strain and an average of 58% after five cycles, whereas solvent-triggered gel recovers only 10% of the initial value and maintains the value over the cycles (Figure 3G, Table 2). In the same context, in the same year, Fan and Sun et al. reported biphenyl acetic acid (BPAA)-capped dipeptide, D_2_P8 (Figure 3K), that self-assembles to form transparent thixotropic hydrogels by temperature switching or ion induction with a nanofibrillar network structure [99]. The biphenyl group is composed of two adjacent phenyl rings. The delicacy of the group is that the rings cannot adopt a stable plane but can quickly adopt a twisted equilibrium geometry with a dihedral angle and create a new aromatic capping group along with Nap, Fmoc, pyrene, etc., to serve as a building block for LMWGs. In addition, in this context, Gazit et al. showed the self-healing thixotropic hydrogel of carboxybenzyl (Cbz)-capped dipeptide, D_2_P9, by adopting *β*-sheet arrangements (Figure 3L) [75].

**Figure 4:**
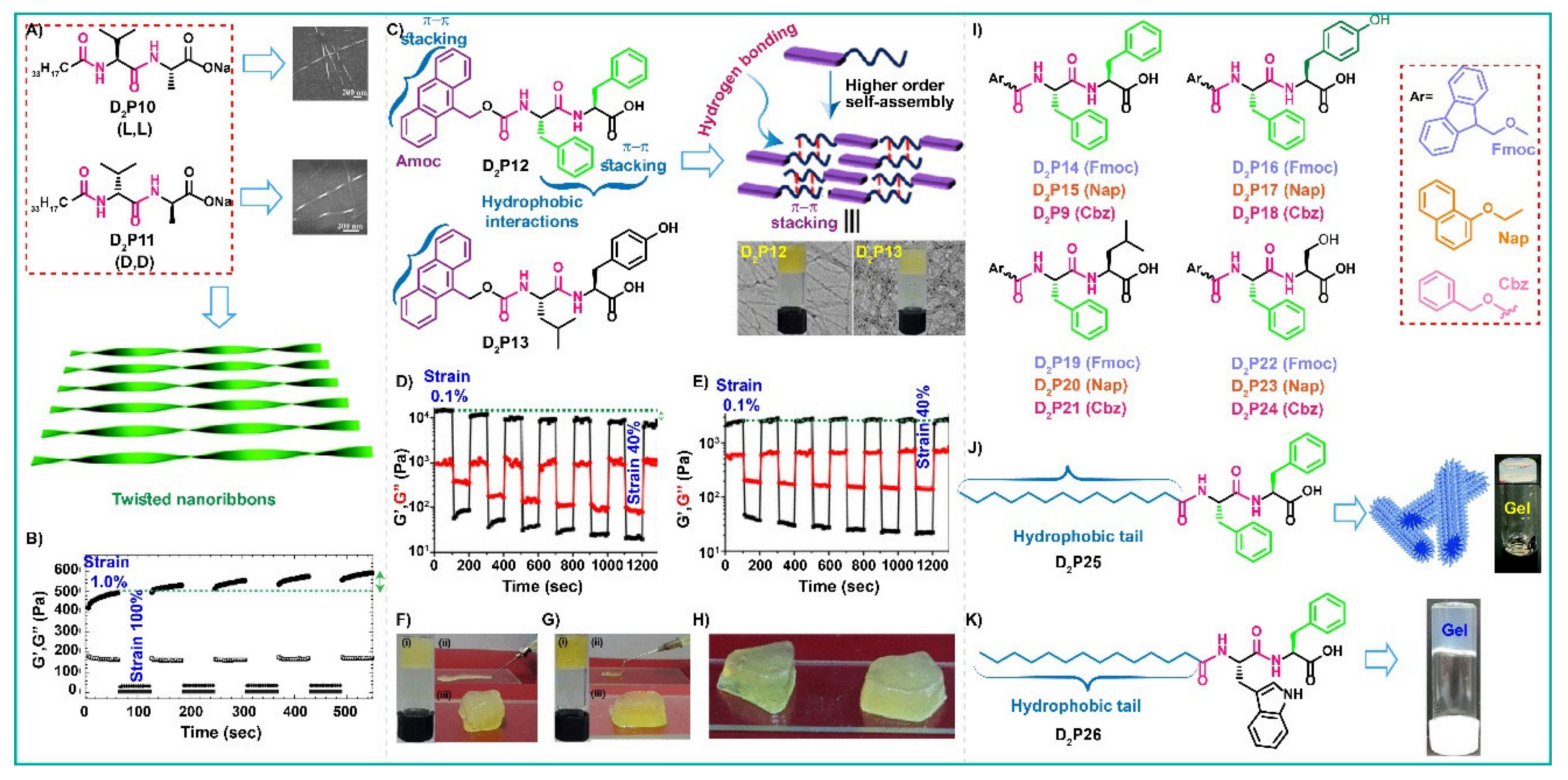
(**A**) Molecular structures and FE-SEM images of the lipodipeptide hydrogelators (D_2_P10 and D_2_P11); Schematic illustration of twisted nanoribbons. (**B**) Rheological step-strain measurements of the respective lipodipeptide hydrogels; reproduced with permission from Ref. [78]. Copyright (2018) Royal Society of Chemistry. (**C**) Chemical structures, schematic illustration for self-assembly to form the gels and TEM images of the resultant hydrogels. (**D**,**E**) Thixotropy test of the dipeptide (D_2_P12 and D_2_P13) hydrogel. (**F**,**G**) Self-supporting hydrogel images: (i) inverted vial confirming gelation character; (ii) injectable nature of the hydrogels; (iii) macroscopic hydrogel block; (**H**) macroscopic-shaped (triangular and pentagonal) hydrogel; reproduced with permission from Ref. [79]. Copyright (2018) American Chemical Society. (**I**) Molecular structure of the self-assembled peptide hydrogelators (D_2_P14-D_2_P24); redrawn from Ref. [76]. Copyright is not applicable here. (**J**) Chemical structure and photographs of vial containing hydrogels; reproduced with permission from Ref. [80]. Copyright (2020) Royal Society of Chemistry. (**K**) Chemical structure and photographs of vial containing hydrogels; reproduced with permission from Ref. [81]. Copyright (2020) American Chemical Society.

To explore the role of other amino acids to prepare thixotropic hydrogels, Yang and Li et al. utilized two homochiral (L, D) amino acids such as valine (Val) and alanine (Ala) sodium salts to prepare D_2_P10 and D_2_P11 in which the N-terminal of the sequence was-capped with palmitic acid (C_17_H_35_COOH) to form translucent hydrogels with twisted nanoribbons and thixotropic behavior (Figure 4A,B, Table 2) [78]. Thixotropic behavior can be explained in terms of strong H-bonding interactions between the monomer molecules. Remarkably, the gel prepared from D-version displayed good injectability behavior, as can be reflected in the longer gel thread in 1.0 mM HCl solution. In the same connection, Das and co-workers reported a series of 9-anthracenemethoxycarbonyl (Amoc)-capped dipeptides employing Phe, Tyr, and isoleucine (Ile) amino acid [79]. The Amoc group is typically used to create peptide-based hydrogelators because of the unique self-assembling nature through their inherent aromaticity and hydrophobicity of anthracene unit [100,101]. The synthesized peptides (D_2_P12 and D_2_P13) forms injectable and self-supporting hydrogels at pH 7.4 through antiparallel *β*-sheet-like structural arrangement, endowed by noncovalent interactions such as H-bonding, hydrophobic, and π-π stacking (Figure 4C). G′ was found to be higher than G″ for D_2_P12 (γ = 0.5%) and D_2_P13 (γ = 0.1%), respectively, suggesting a viscoelastic nature with a cross-linked gel network (Table 2). However, the rupture of the gel network followed by a liquid-like sol state was observed when the strain was increased to 40% for both gels, as confirmed by the lower G′ value compared to G″ from strain-sweep experiments (Figure 4D,E). Again, when the lower strain was applied to these gels for 100 s, immediate reformation of the cross-linked network was observed, and as a result, the mechanical properties (G′ > G″) of these hydrogels were restored. This process was continued for another five cycles, confirming the good self-healing abilities of these hydrogels. However, D_2_P12 exhibited better self-healing, as can be ensured by almost 100% recovery of their moduli than D_2_P13, which can be attributed to the dynamic deformation–reconstruction of the three-dimensional fibrillar network. The injectability properties and shape-memory hydrogels are shown in Figure 4F–H.

In 2019, Roy et al. demonstrated a series of ultrashort amyloid-like peptides in which the N-terminal of the peptide was capped with different aromatic groups such as Cbz, Nap, and Fmoc, with Phe as the common amino acid (D_2_P9, D_2_P14-D_2_P24, Figure 4I) [76]. C-terminal with different functional amino acids, including aromatic Phe, polar aromatic Tyr, nonpolar aliphatic leucine (Leu), and polar aliphatic serine (Ser), covering a broad range of hydrophobicity, undergoes self-assembly to form thixotropic hydrogels with fibrillar nanostructures (Figure 4I). To enlighten the importance of sequence hydrophobicity, the peptides were rationally designed to drive the self-assembly propensity in an aqueous medium. The thixotropy property of the gels was established with the help of the step-strain experiment, and it was observed that Fmoc-capped molecules recovered 73% value of G′ and G″, whereas 93% and 95% recovery was observed after 60 sec for Nap- and CbZ–capped peptides (Table 2). This can be explained in terms of the hydrophobicity of the capped aromatic group, which is inversely proportional to the recovery rate of the gels. In the same direction, the myristic group-capped dipeptides self-organized into thixotropic hydrogels at physiological conditions reported by Kraatz [80] and Banerjee [81] (D_2_P25, D_2_P26, Figure 4J).

**Figure 5:**
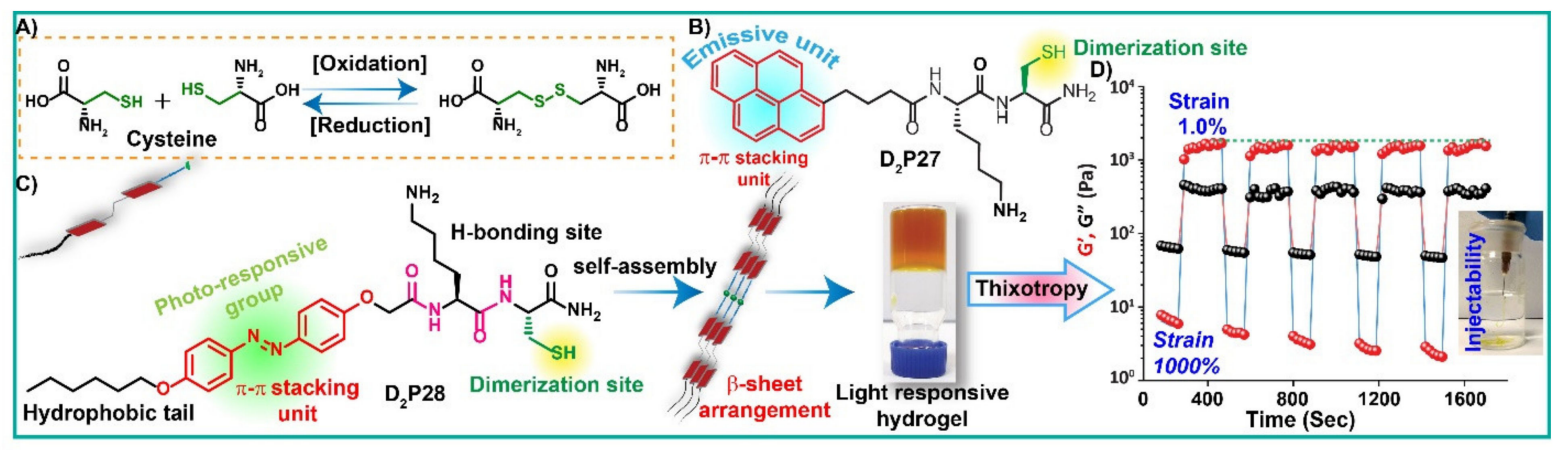
(**A**) Dimerization of two cysteine units. (**B**) Chemical structure of the pyrene-capped dipeptide hydrogelator (D_2_P27); redrawn from Ref. [40]. Copyright is not applicable here. (**C**) Molecular structure and possible self-assembly of the Azo-capped dipeptide hydrogelator (D_2_P28). (**D**) Rheological step-strain analysis of D_2_P28, confirming thixotropicity. Inset: injectability behavior; reproduced with permission from Ref. [82]. Copyright (2020) Royal Society of Chemistry.

Cysteine (Cys) is one of the vital amino acids and is prone to form disulfide bonds (–S–S–) in basic pH or under aerobic conditions, or by oxidation of dissolved oxygen in solvents (Figure 5A) [40,82,102,103]. Disulfide chemistry has been found to be of significant importance in biological systems in order to stabilize the protein’s secondary and tertiary structures [3]. By considering this, our group explored pyrene-capped dipeptide, D_2_P27 (Figure 5B), which forms a self-supporting hydrogel in tris buffer at pH 8 (Table 2) through H-bonding, -S-S-dimerization in addition to π-π stacking of pyrene units [40]. The beauty of the gel is its insolubility in water. Inspired by this unique property, in 2020, our group designed and reported azobenzene-capped dipeptide, D_2_P28, which undergoes self-assembly in a fresh NaOH solution to afford a self-supporting hydrogel (Figure 5C) [82]. The gel is thixotropic by virtue, as confirmed by the time-dependent strain-sweep rheological experiment. At a constant strain (γ = 0.1%), the gel exhibited its elastic behavior (G′ > G″), but the gel lost its elasticity when a higher strain (γ = 1000%) was applied. The gel transformed into a quasi-liquid state, and the material recovered its mechanical properties almost completely within a short stretch of time after the release of strain (Table 2). This step-strain experiment was continued for another four cycles, confirming good injectability, as shown in Figure 5D.

**Figure 6:**
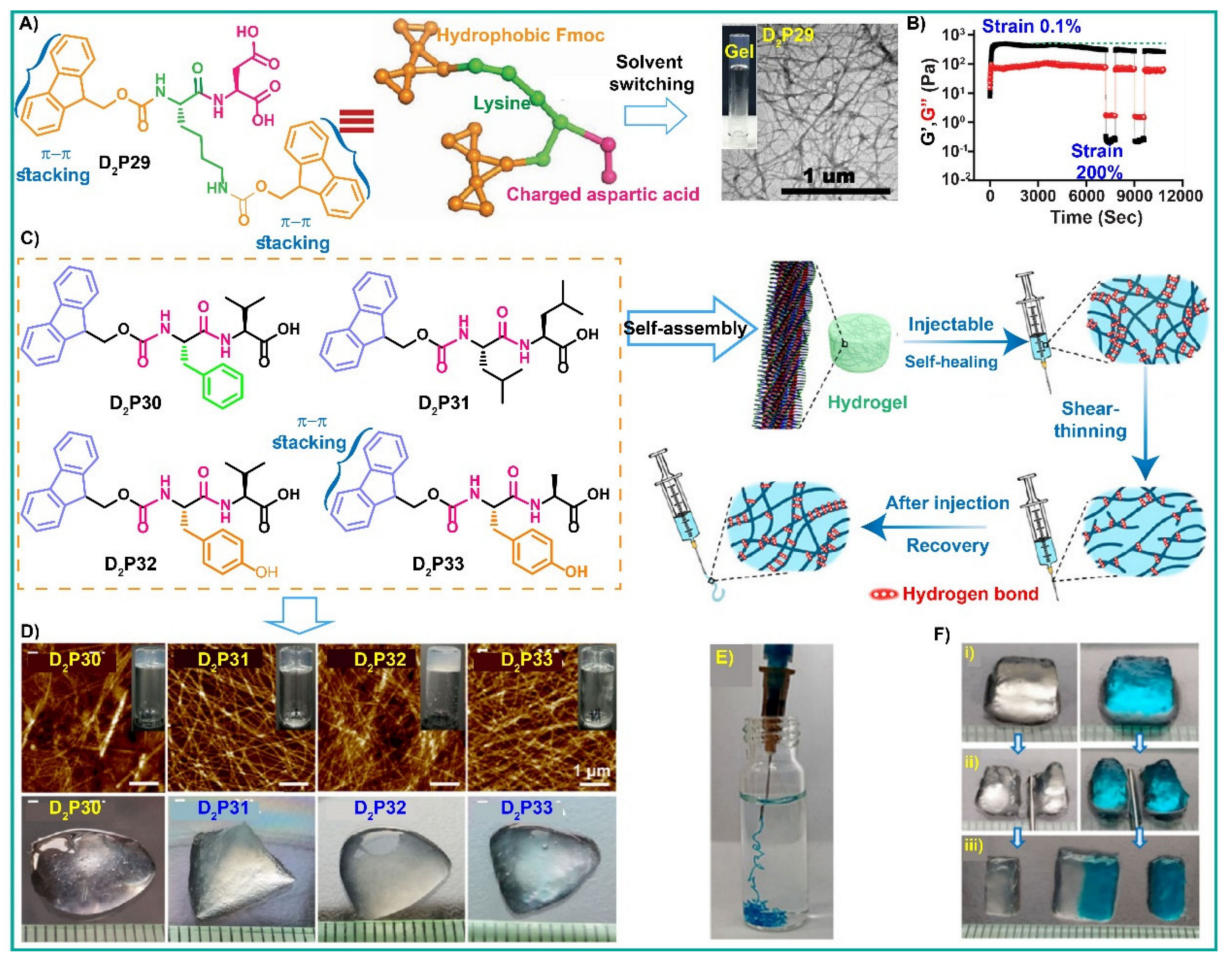
(**A**) Chemical structure and TEM image of the dipeptide hydrogelator (D_2_P29). (**B**) Thixotropy test of D_2_P29 hydrogel; reproduced with permission from Ref. [83]. Copyright (2020). John Wiley and Sons. (**C**) Molecular structures and schematic representation of the dipeptide gelators (D_2_P30-D_2_P33) for the self-thinning and self-healing behavior. (**D**) AFM images and photographs of 26-gauge needles injected into dipeptide hydrogels. (**E**) Injectability behavior of methylene blue-stained hydrogel (D_2_P32). (**F**) Self-healing behavior of D_2_P32 hydrogel; reproduced with permission from Ref. [84] Copyright (2020) American Chemical Society.

In the same year, Gazit, Wei and co-workers reported an Fmoc-capped minimalistic de novo dipeptide hydrogelator, composed of Lys with an additional Fmoc group and aspartic acid (Asp). D_2_P29 forms a hydrogel at 0.002 wt% (28.3 × 10^−6^ M), the lowest critical gelation concentration (CGC) ever (Figure 6A, Table 2) [83]. The gelator was found to be thixotropic in nature, as revealed by the time-dependent rheological step-strain measurement (Figure 6B). Next, Bai, Li, and co-workers reported a series of Fmoc-capped dipeptides, D_2_P30, D_2_P31, D_2_P32 and D_2_P33, which self-assembled into PBS buffer solution to form colorless and transparent (D_2_P32 and D_2_P33), semi-transparent (D_2_P30), and opalescent (D_2_P31) hydrogels (Figure 6C, Table 2) [84]. When the hydrogels were injected through a 26-gauge syringe needle to give a tetrahedral shape, the D_2_P32 hydrogel retained the shape well, whereas D_2_P33 displayed a slightly collapsed structure, D_2_P31 exhibited a weak gel state, and phase separation was observed in the case of D_2_P30. This can be explained by differential mechanical behaviors as accessed by rheological measurements. The presence of the phenolic -OH group in Tyr offered more H-bonding interaction, strengthening the mechanical properties. As shown in Figure 6E, 1% methylene blue staining D_2_P32 hydrogel showed injectable properties and stability in PBS solution. In addition, the D_2_P32 exhibited good self-healing abilities, as the two dissected parts conglutinated immediately into an integrated block when they were kept closer (Figure 6F).

**Figure 7:**
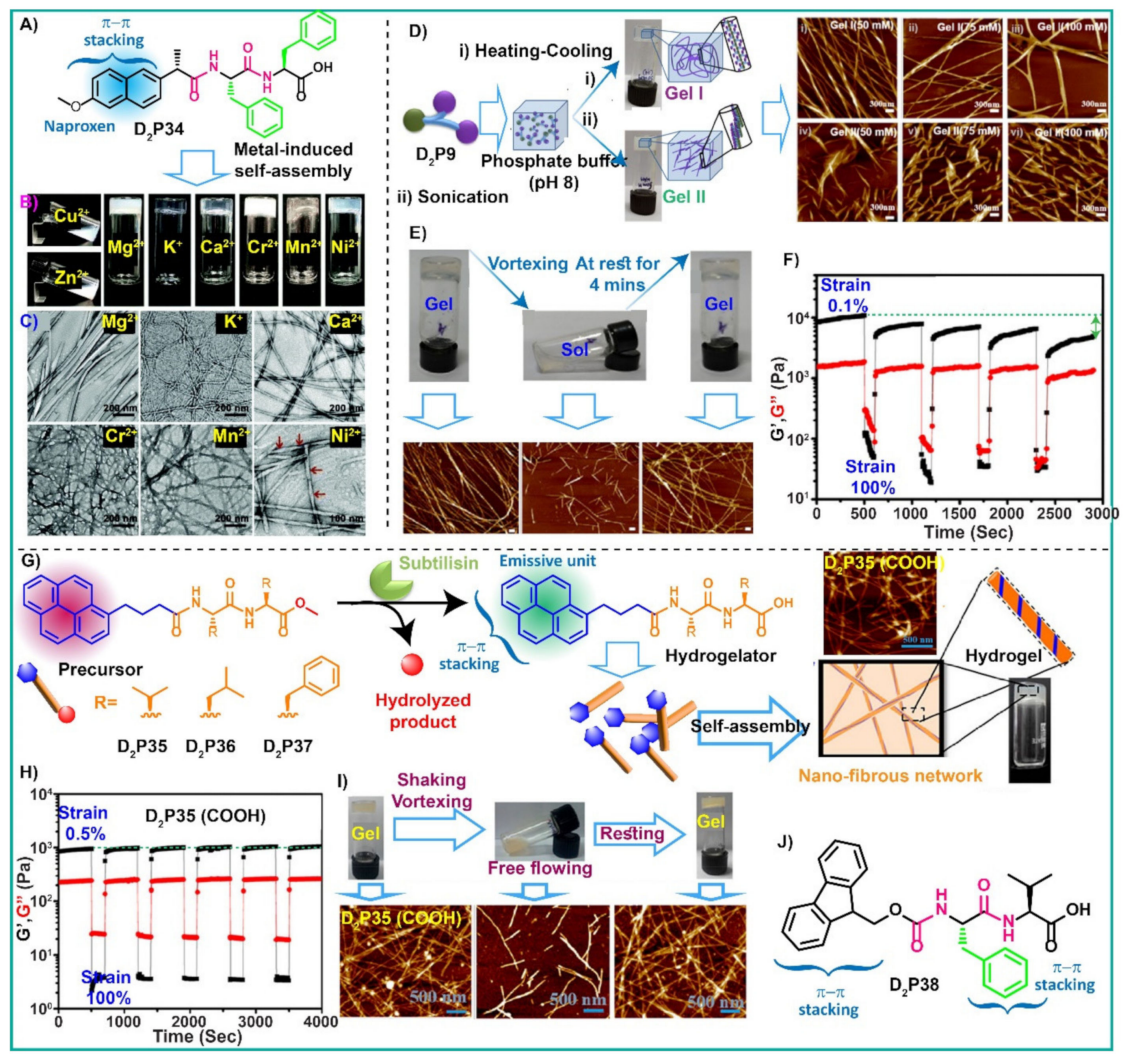
(**A**) Chemical structure of the dipeptide hydrogelator (D2P34). (**B**) Metal-induced self-assembly and hydrogel formation. (**C**) TEM images of the respective metallogel; reproduced with permission from Ref. [85] Copyright (2020) Royal Society of Chemistry. (**D**) Schematic illustration of gel formation and the AFM images of the resultant gels. (**E**) Self-healing behavior of the hydrogel with the representative AFM images. (**F**) Rheology experiment confirming the thixotropic behavior of the hydrogels; reproduced with permission from Ref. [77]. Copyright (2020) American Chemical Society. (**G**) Molecular structures of the hydrogelators, representative self-assembly process followed by gel formation with AFM image. (**H**) Step-strain rheological analysis for thixotropicity. (**I**) Self-healing ability of the gel with demonstrative AFM images. Reproduced with permission from Ref. [86] Copyright (2021) American Chemical Society. (**J**) Chemical structure of the dipeptide hydrogelator; redrawn from Ref. [87]. Copyright is not applicable here.

The naproxen-capped PhePhe dipeptide (D_2_P34, Figure 7A, Table 2) also experiences metal ion-induced thixotropic hydrogel formation developed by Chen and colleagues [85]. In the same year, Roy et al. utilized the PhePhe dipeptide motif in which the N-terminal of the sequence is capped with Cbz moiety (D_2_P9), which self-assembles in PBS buffer at pH 8 by employing different pathways (heating–cooling method and sonication) to form self-supporting thixotropic gels as shown in Figure 7D [77]. The gel was transformed into a solution owing to vigorous shaking, and upon free-standing, the gel state reappeared, which can be reflected from AFM images as well, indicating a self-healing ability (Figure 7E). At a constant strain (γ = 0.1%), the gel maintained its viscoelastic nature (G′ > G″) due to the nanofiber’s architecture. However, when the strain was increased (γ = 100%), the gel structure ruptured due to the dissolution of the long fibers to give a liquid-like state (G′ < G″), and upon rest, the short fibers came together to reform the fiber network followed by gel (G′ > G″) formation (Figure 7F, Table 2). The gel failed to regain its initial G′ value after each step, and the recovery percentage was found to be 72% and 82% for gel I and gel II, respectively.

To expand the scope, the same group explored a series of pyrene-capped dipeptides (D_2_P35, D_2_P36 and D_2_P37) methyl ester as a molecular switch motif [17]. In the presence of subtilisin, a hydrolytic enzyme from *Bacillus licheniformis*, the peptide derivatives undergo hydrolysis of methyl ester to form the corresponding acid derivatives, which self-assembled through noncovalent interactions to form a thixotropic hydrogel (Figure 7G, Table 2). The D_2_P35 hydrogel showed excellent moduli recovery after removing high strain, whereas the D_2_P37 hydrogel displayed 93.58% recovery, indicating good self-healing ability (Figure 7H,I). Najafi and Tamaddon et al. demonstrated the self-assembly behavior of D_2_P9, which adopted antiparallel *β*-sheet arrangement to form a self-supporting thixotropic hydrogel with an entangled fibrillar network in alkaline NaOH solution followed by HCl treatment [87]. In the same year, the same group stated the injectable behavior of self-supporting hydrogel formed by the Fmoc-capped dipeptide, Fmoc-Phe-Val (D_2_P38, Figure 7J, Table 2) in PBS at pH 7.4 by a pH-titration procedure through *β*-sheet nanofiber arrangements [104].

In this section, a dipeptide-based supramolecular hydrogel with its structural and functional diversity is discussed. The synergistic effect of the capped aromatic units with the monomer units’ noncovalent interactions help form self-assembled peptide hydrogels with thixotropic behavior. It was noted that many stimuli, including pH, temperature, metal ions, sonication, solvents, ionic strength, etc., played a vital role in creating hydrogels with nanoscale architectures. Out of the above-mentioned dipeptide hydrogelators, some are showing (D_2_P12, D_2_P28, D_2_P37) excellent recovery of their initial moduli value after removing high strain with good injectable property.

## 4. Cyclic Dipeptide (CDP)-Based Thixotropic Hydrogel

**Figure 8:**
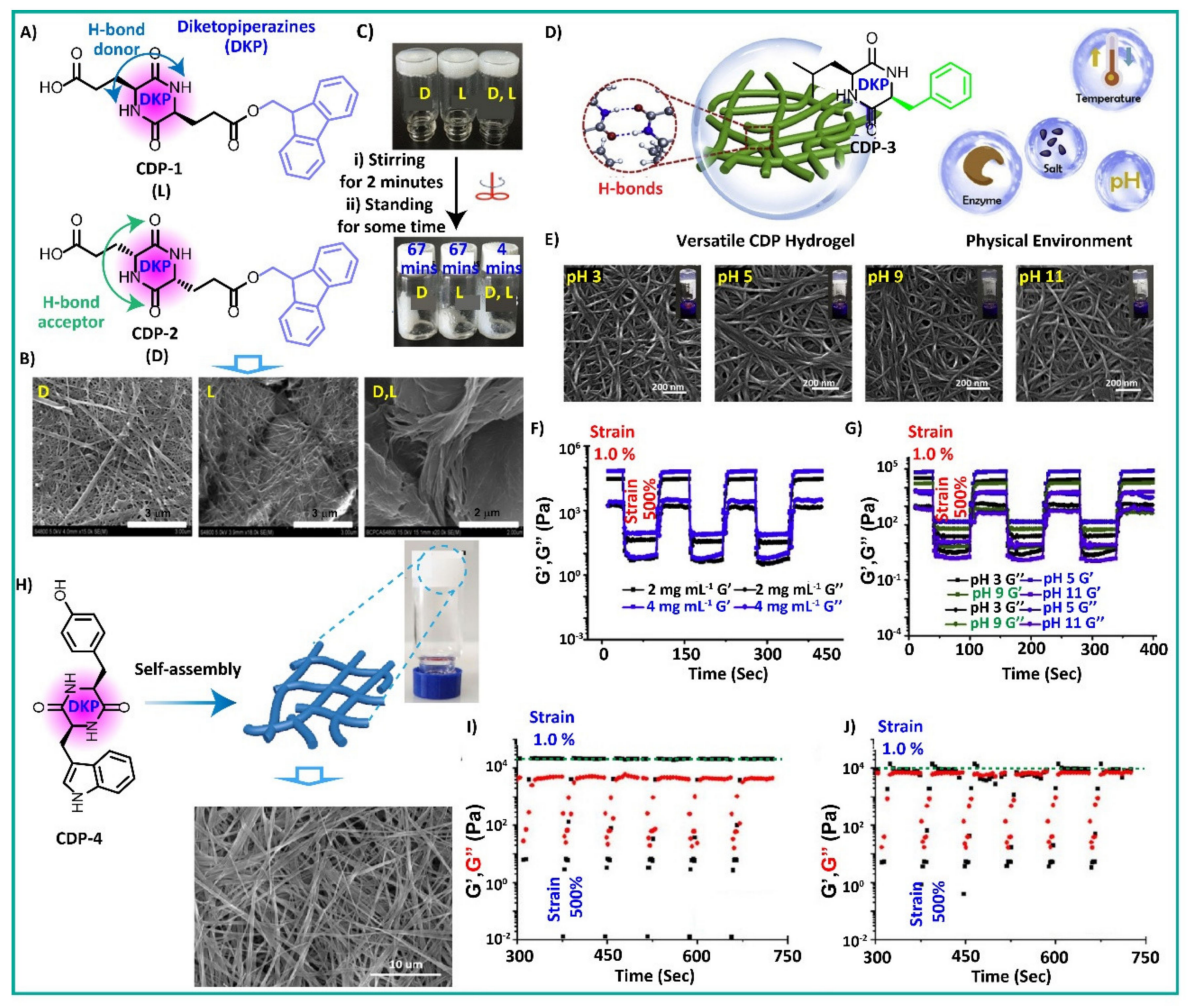
(**A**) Molecular structure. (**B**) FE-SEM images. (**C**) Vial containing hydrogel photographs of the cyclic dipeptide hydrogelators (CDP-1 and CDP-2); reproduced with permission from Ref. [105]. Copyright (2017) American Chemical Society. (**D**) Chemical structure and self-assembly schematic illustration of the cyclic dipeptide hydrogelators (CDP-3). (**E**) FESEM images of CDP-3 at different pH. (**F**,**G**) Rheological step-strain measurements of the respective hydrogels; reproduced with permission from Ref. [108]. Copyright (2019). Elsevier. (**H**) Chemical structure, gelation studies and FE-SEM image of the CDP-4. (**I**,**J**) Thixotropy test of the CDP-4 hydrogel; reproduced with permission from Ref. [106]. No copyright is needed, as this is an Open Access article under the terms of the Creative Commons Attribution License.

Along with linear dipeptides, cyclic dipeptides (CDPs) have also received considerable interest in the biological and pharmacological field. CDPs are a distinct type of small cyclic form of peptides with heterocyclic 2,5-diketopiperazines building blocks (Figure 8) [105,106,107]. In recent years, CDP has gained increasing attention in the field of self-assembly to form supramolecular gels owing to the presence of four H-bonding sites (two donors and two receptors) and other noncovalent forces, including electrostatic interactions, π-π stacking, hydrophobic effect, van der Waals forces [105,107,108]. In addition, CDPs are molecularly rigid in nature, conformationally constrained structures, and they exhibit low enzymatic degradation as well as high physiological stability in comparison to their linear analogues, which make CDPs ideal building blocks for self-assembly studies to create unique and unusual assemblies [107,109,110]. Therefore, dissimilar self-assembly performances and stability of peptides with linear and cyclic analogues can be expected. For example, Lim et al. reported the different self-assembly behavior between linear and cyclic peptide [110]. Considering all these facts, Feng, Bezuidenhout, and co-workers designed two thixotropic chiral CDP supergelators, namely cyclo[LGlu(OFmoc)-L-Glu] (CDP-1) and cyclo[D-Glu(OFmoc)-D-Glu] (CDP-2) (Figure 8A) [105]. Both the chiral CDPs undergo self-assembly to form a hydrogel (MGC 0.4 wt%, Table 3) with a uniform densely packed tangled nanofiber, whereas the racemate, D, L (MGC 0.6 wt%) forms comparatively loosely clumped fibers with a large width, indicating the impact of chirality on the self-assembled structure (Figure 8B). The gels were mechanically stirred into a sol state to test the thixotropic properties (Figure 8C). The CDP-2 and racemate gel recovered around 93% and 89% of their original G′ value upon resting for 67 and 4 min, respectively. Thereafter, in case of the racemate hydrogel, it was observed that in the second and third cycle, the G′ value increased gradually, even higher than the native ones, implying high stability of the gel after disintegration. The pristine hydrogel’s comparatively lower G′ value can be attributed to the random arrangements of chiral enantiomers of racemate hydrogels to form nanocrystalline self-assemblies.

Later, another CDP, cyclo-(Leu-Phe) (CDP-3, Figure 8D), was reported by Yan et al., which experiences self-assembly to form a nontransparent thixotropic hydrogel with crystal features and close-knit three-dimensional fibrous network structures (Table 3) [106]. The intrinsic intermolecular hydrogen bonding ability between CDP-3 peptides imparts super gelation capability to the CDP with almost complete recovery of their original moduli values, confirming a self-healing ability. Intuitively, the mechanical stability of the supergelator is enhanced with an increase in gelator concentration because of the greater H-bonding between CDP-3 nanofibers and water molecules, but it does not affect the self-healing propensity of the hydrogel (Figure 8F). In addition, CDP-3 forms a transparent thixotropic hydrogel at different pH, from acidic (pH 3) to basic (pH 11), with intertwined long nanofibers (Figure 8E), indicating the versatility of the gelator (Figure 8G). Furthermore, CDP-3 maintains the thixotropic nature even in the physiological solutions containing phosphate buffer saline (PBS), Dulbecco’s Modified Eagle Medium (DMEM), or trypsin. Therefore, CDP-3 can be used as a unique biomaterial for biomedical applications in harsh biological or chemical environments. Later, in the same year, the same group demonstrated the self-assembly of cyclo-(Trp-Tyr), CDP-4 to form a thixotropic hydrogel with crystal features and close-knit three-dimensional network structures (Figure 8H, Table 3) [108]. The CDP displayed better mechanical properties upon aging. The alternate high (γ = 500%) and low (γ = 1%) step-strain rheological experiment exemplified that the recovery time is highly dependent on aging time. Therefore, the hydrogel at 240 h exhibited a comparatively quicker recovery speed than the gel at 48 h (Figure 8I,J).

CDP3 demonstrated outstanding self-healing properties among the cyclic dipeptides discussed. The capacity to self-heal is invariant primarily to gelator concentration, pH (3–11), and even buffer, making them excellent gelators with thixotropic properties. Compared to linear analogs, it is difficult to compare the thixotropic behavior of these CDPs. Both type of peptides showed 3D fibrous structures in the gel network with good gel recovery. The complex and monotonous purification process of CDPs and higher MGC compared to the linear part makes them less explored in terms of thixotropic properties.

## 5. Tripeptide (T_3_P)-Based Thixotropic Hydrogel

**Figure 9:**
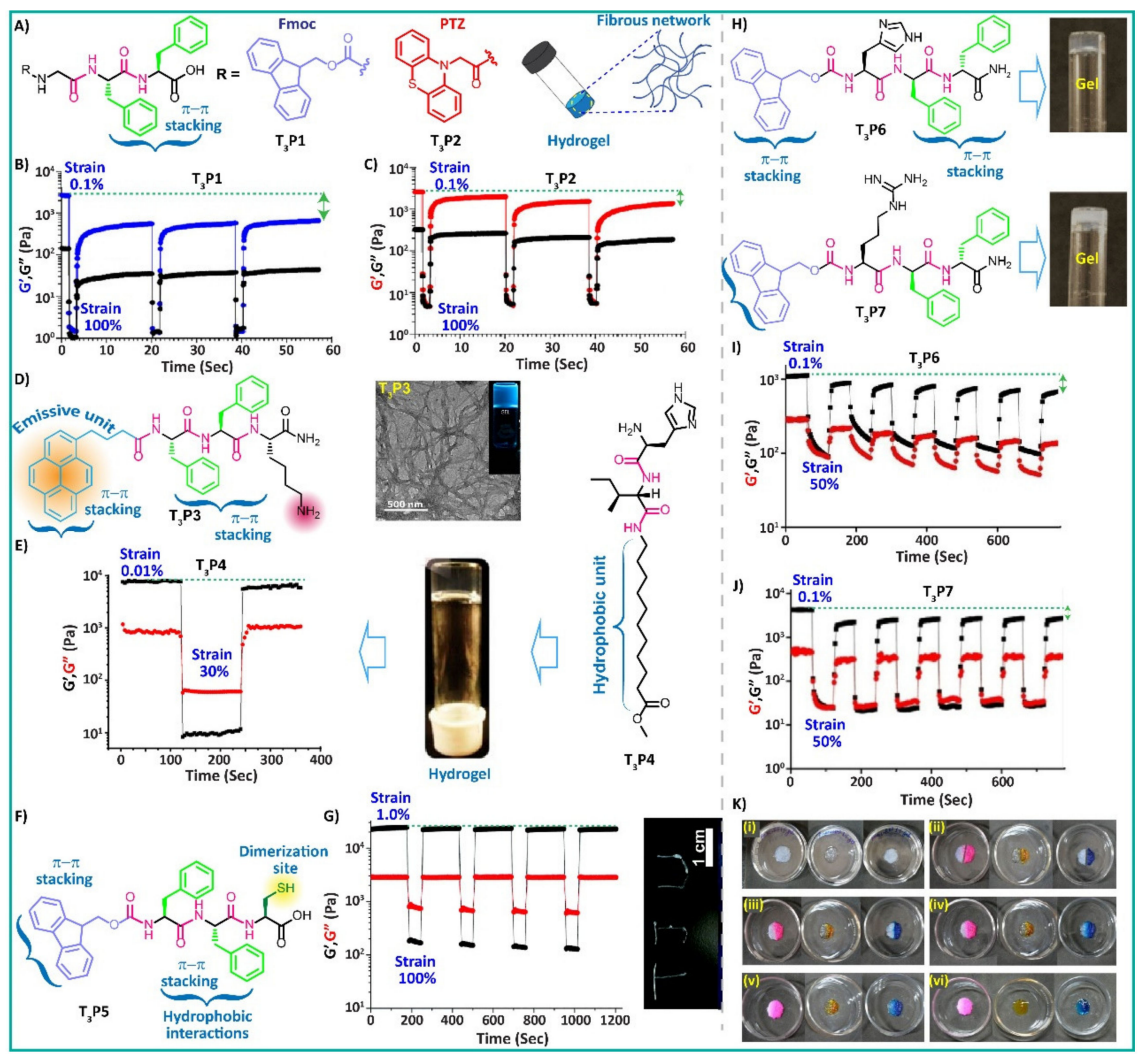
(**A**) Chemical structure and self-assembly of the tripeptide hydrogelators (T_3_P1 and T_3_P2). (**B**,**C**) Thixotropic step-strain experiment of the tripeptide gelators; reproduced with permission from Ref. [111]. Copyright (2017) John Wiley and Sons. (**D**) Molecular structure, TEM image of the tripeptide (T_3_P3) hydrogelator; reproduced with permission from Ref. [57]. Copyright (2019) American Chemical Society. (**E**) Chemical structure, hydrogel and recovery test of the hydrogelator T_3_P4; reproduced with permission from Ref. [113]. Copyright (2019) American Chemical Society. (**F**) Chemical structure. (**G**)Thixotropic analysis and injectability of the hydrogelator T_3_P5; reproduced with permission from Ref. [114]. Copyright (2019) The Royal Society of Chemistry. (**H**) Molecular structure and photographs of the hydrogelator (T_3_P6 and T_3_P7). (**I**,**J**) Rheology experiment confirming the thixotropic behavior of T_3_P6 and T_3_P7. (**K**) Self-healing test of macroscopic hydrogels; reproduced with permission from Ref. [115]. Copyright (2020) American Chemical Society.

Typically, peptide-based LMWGs are made of N-terminal-capped aromatic moieties such as benzene, Nap, pyrene, Fmoc, Amoc, etc., for π-π interactions and a *β*-sheet-forming peptide motif to drive the self-assembly process [5,26,35,40]. Thus, by taking those interactions into attention and inspired by the well-known *β*-sheet forming tripeptide motif, GlyPhePhe, in 2017, Thordarson et al. anticipated different aromatic (Fmoc and PTZ)-capped tripeptides (T_3_P1 and T_3_P2), which undergo self-assembly in DMEM to form a supramolecular hydrogel with thixotropic properties (Figure 9A, Table 4) [111]. Phenothiazine-10-acetic acid (PTZ) is a crucial heterocyclic ring and is widely used in pharmaceuticals owing to its good biocompatibility. It is well documented in the literature that the presence of a PTZ unit endowed the super gelation ability in LMWGs [112]. In addition, the metal ions in the DMEM medium promoted the gelation through charge screening, and a critical gelation concentration for PTZ and Fmoc-capped hydrogel was found to be 0.05 (*w*/*v*) and (0.1 *w*/*v*), respectively. Both hydrogels displayed moderate thixotropic behavior, as can be seen from Figure 9B,C. After withdrawal of high shear force, the gel recovers only 21% and 79% of the G′ value for Fmoc and PTZ-capped gels, respectively. Unfortunately, PTZ-capped hydrogel lost the recovery rate from 79% to 60% to 53% in the subsequent cycles (Figure 9C). In contrast, the Fmoc-capped hydrogel showed a nominal improved recovery rate from 21% to 22% to 25% (Figure 9B), implying the role of the capping group. However, the thixotropic properties were raised from the gel microstructures from the GlyPhePhe unit. The ordered *β*-sheet structure in the PTZ-capped hydrogel may be responsible for such a type of mechanical behavior. Likewise, in 2019, our group reported a pyrene-capped tripeptide, Py-PhePheLys (T_3_P3, Figure 9D, Table 4), forming a self-supporting thixotropic hydrogel in 1:1 ACN:water with a fibrous network [57]. PhePheLys is another well-studied *β*-sheet-forming sequence. In the same year, Banerjee’s group described a C-terminal-protected tripeptide sequence, composed of His, Ile, and an unnatural amino acid, 11-aminoundecanoic acid (AUDA), for the self-assembly in PBS in a broad pH range from 5.5–8.5 to form a transparent hydrogel with thixotropic behavior (Figure 9E) [113].

Next, Qi and Wang et al. fabricated a tripeptide in which the N-terminal of the peptide was capped with an Fmoc group. The central part is composed of a well-known self-assembling PhePhe motif, and Cys at the C-terminal for the crosslinking of the sulfhydryl group undergoes self-assembly in 0.5 M NaOH solution to form a stable, self-supporting hydrogel with a helical network (Figure 9F, Table 4) [114]. The thixotropic behavior of the hydrogel was evaluated with the help of a time-dependent step-strain experiment in which alternation of low (γ = 1.0%) and high (γ = 100%) strain was applied to check gel-sol-gel transitions (Figure 9G). The hydrogel completely recovered its initial moduli after four cycles, indicating good self-healing ability. In 2020, Singh et al. reported an Fmoc-capped tripeptide composed of a popular dipeptide motif, PhePhe in the D form, owing to its high proteolytic stability and tendency for enhanced gelation, and a cationic amino acid Arg or His in L-form because of the potent bactericidal activities (Figure 9H, Table 4) [115]. These peptides displayed self-assembled fibrillar morphology with thixotropic behavior. A time-dependent alienating step-strain experiment showed that at a lower strain (γ = 0.1%), the system perseveres its gel character (G′ > G″). When the high strain was applied to the system (γ = 50%), the gel collapsed to form a liquid state (G′ < G″). However, as the strain was dropped to the original one, the system regained its 3D fiber network, confirming the self-healing behavior. The process was repeated for another five cycles (Figure 9I,J). The gel failed to recover its initial G′ value after the first destruction of the gel, and after that, the gel maintained the G′ value for the rest of the cycles, as can be seen in Figure 9I,J. In addition, the self-healing ability of the hydrogel was established macroscopically (Figure 9K). Therefore, the gels were cut into two equal halves with the help of a surgical blade, one of the parts was stained with a dye, and the other part was kept intact. After 9 h of rest, the gel showed excellent self-healing properties, as the dye was diffused from the stained region to the unstained one. However, the T_3_P6 gel failed to retain its strength after 9 h of self-healing.

**Figure 10:**
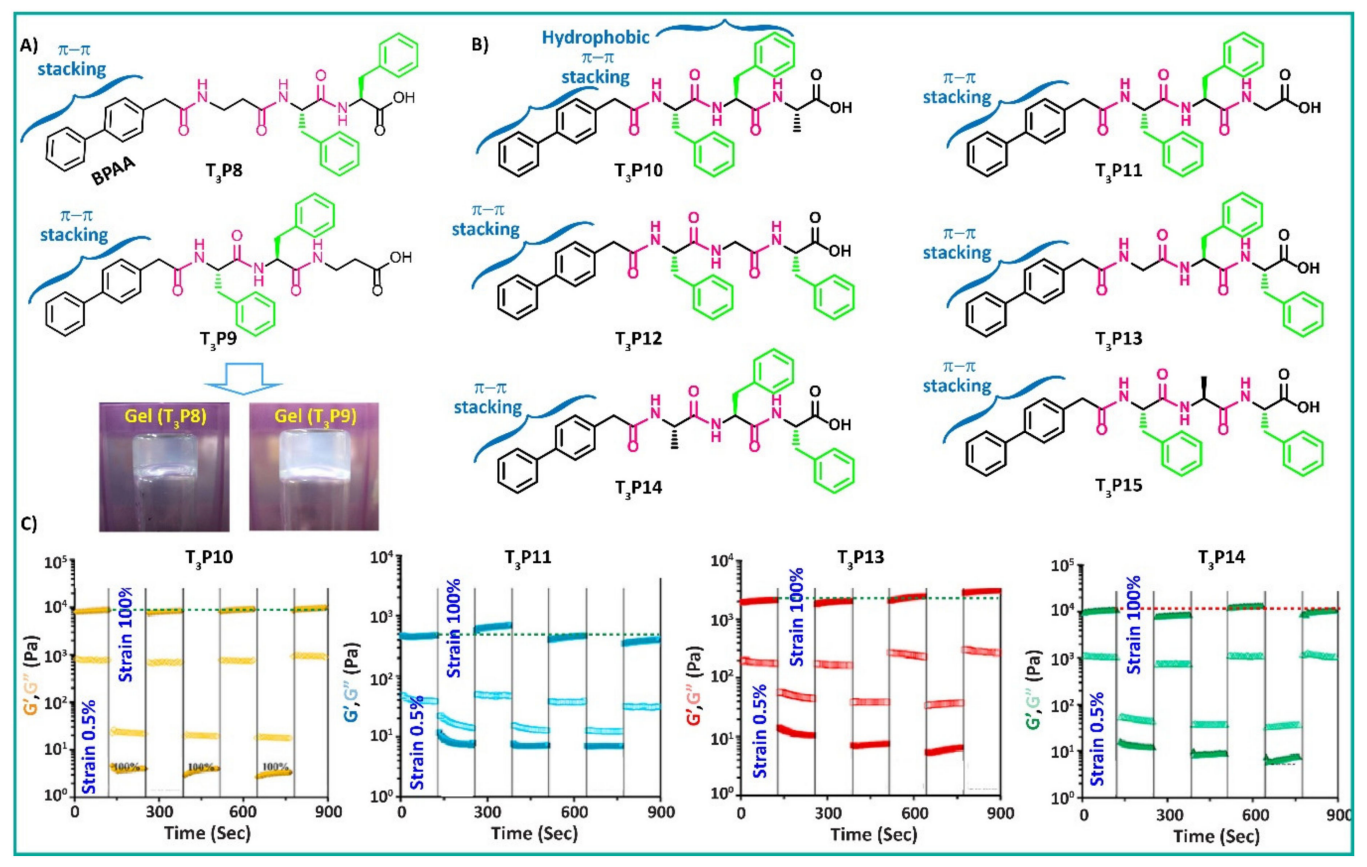
(**A**) Chemical structure of the hydrogelator (T_3_P8 and T_3_P9) with the hydrogel vials; reproduced with permission from Ref. [116]. Copyright (2021) Elsevier. (**B**) Molecular structures of BPAA-capped tripeptide hydrogelators (T_3_P10-T_3_P15). (**C**) Thixotropy test of the above mentioned in (**B**) hydrogels; reproduced with permission from Ref. [117]. No copyright is needed, as this is an Open Access article under the terms of the Creative Commons Attribution License.

Within this context, 4-biphenyl acetic acid (BPAA)-capped tripeptides (T_3_P8 and T_3_P9, Figure 10A) were reported by Sun, Jiang, Wang and co-workers, which are composed of beta-alanine (*β*-Ala), a well-studied PhePhe dipeptide motif forming a thixotropic hydrogel (Table 4) via a pH switch method through *β*-sheet arrangement promoted by intermolecular H-bonding and π-π stacking interactions [116]. Both hydrogels almost completely recover the moduli at the end of the cycle, as confirmed by the rheological time sweep analysis, confirming good self-healing ability. More recently, a series of BPAA-capped tripeptides (T_3_P10-T_3_P15) was reported by Sun et al. The PhePhe dipeptide motif was introduced as a core segment with different C-terminal amino acids such as Gly, Ala, and Phe followed by an alternative position of Gly and Ala in the sequence (Figure 10B) [117]. All these peptides encounter self-assembly through the pH switch procedure to form a self-supporting gel (Table 4). It was found that T_3_P10 exhibited almost complete recovery of its initial moduli (G′ and G″) compared to T_3_P11, T_3_P13 and T_3_P14. The good self-healing nature of T_3_P10 can be attributed to the presence of a methyl group in Ala, which impeded the flexibility of the T_3_P10 unit, and consequently, conformational entropy of the T_3_P10 peptides decreased (Figure 10C). More recently, Li et al. presented drug–peptide conjugates made of the anti-inflammatory drug, dexamethasone (Dex), and either the ArgGlyAsp or ArgGlyGlu peptide motives undergo self-assembly in PBS at pH 7.4 to form a supramolecular hydrogel with nanotube morphology [119]. The ArgGlyAsp peptide motif was chosen owing to the brilliant trans-corneal permeability and pharmacological activity, which work through ligand-receptor interaction. Both the gels exhibited nice thixotropic behavior with more than 90% recovery of the initial modulus values.

**Figure 11:**
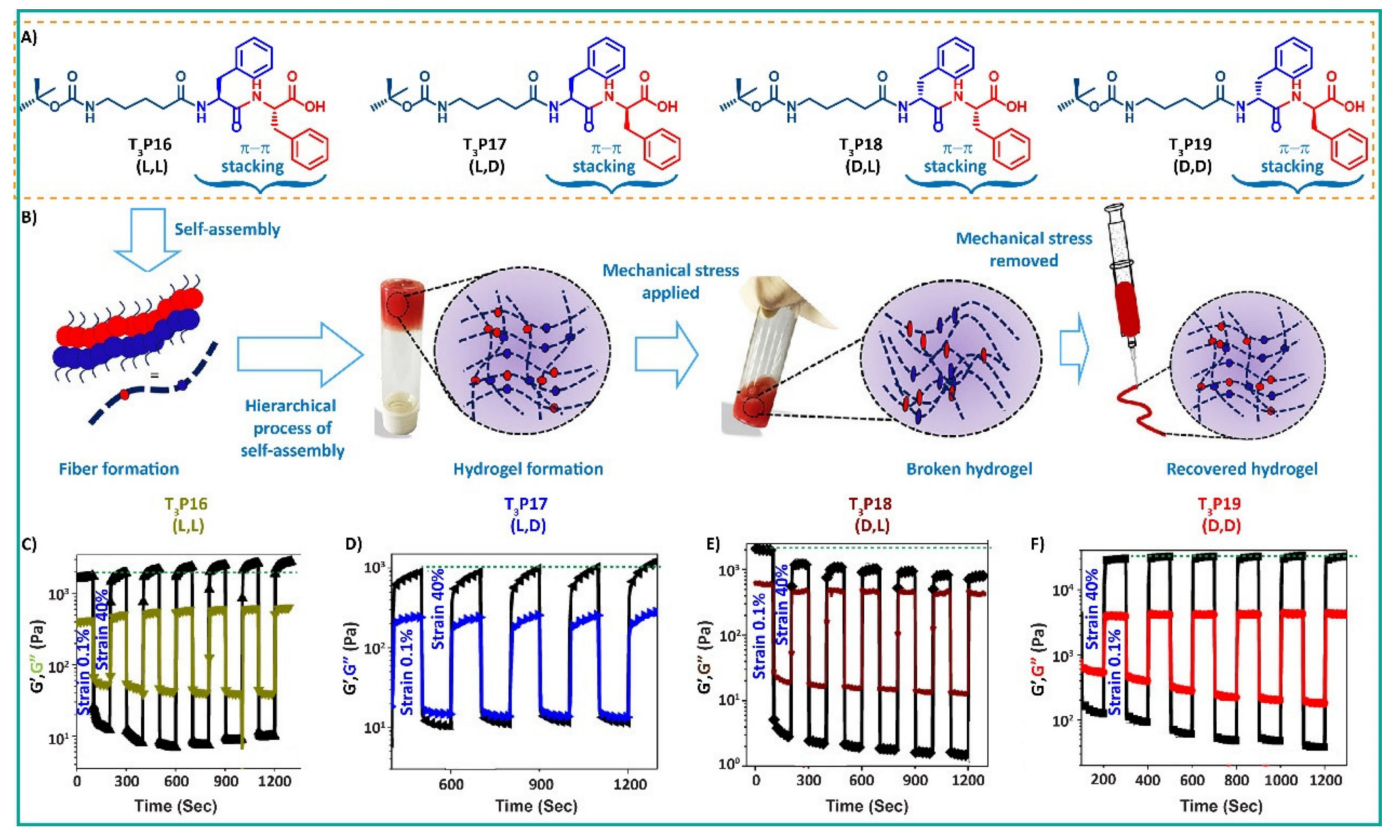
(**A**) Chemical structure. (**B**) Self-assembly, self-healing, injectability schematic illustration of the tripeptide hydrogelators (T_3_P16-T_3_P19). (**C**–**F**) Rheology step-strain experiment confirming the thixotropic behavior of the tripeptide hydrogelators (T_3_P16-T_3_P19); reproduced with permission from Ref. [118]. Copyright (2021) American Chemical Society.

Aligned to the context, Konar et al. fabricated a series of tripeptide gelators (T_3_P16, T_3_P17, T_3_P18 and T_3_P19) by fine tuning the configuration of the building amino acid Phe to investigate the tendency of this construct in rigidifying the solvent system (Figure 11) [118]. The gelators form a supramolecular hydrogel with thixotropic and injectable properties, as can be seen in Figure 11B. The non-covalent interactions and supramolecular *β*-sheet secondary arrangements provide a nanofibrillar morphology to stabilize the hydrogel. Among the series, T_3_P16 (L,L) exhibited the lowest minimum gelation concentration (MGC; 0.7 mg/mL), whereas T_3_P17 (L,D), T_3_P18 (D,L) and T_3_P19 (D,D) showed MGC 1.5, 1.5 and 0.8, respectively (Table 4). The rheological moduli of the hydrogels follow the trend: hydrogelators T_3_P19 (D,D) > T_3_P17 (L,D) > T_3_P16 (L,L) > T_3_P18 (D,L). The trend can be explained in terms of the presence of D-isomers, which strengthen the gels. During the thixotropic recovery test, T_3_P16 (L,L) and T_3_P19 (D,D) completely recovered the moduli within 25 s; however, T_3_P17 (L,D) and T_3_P18 (D,L) took around 100 s to recover the moduli as can be seen from Figure 11C. The difference in their recovery could be attributed to the delayed restoration of non-covalent interactions in the heterochiral derivatives.

The peptide hydrogels, especially drug–peptide supramolecular hydrogelators, are at the forefront of therapeutic biomaterials owing to their brilliant biodegradability, biocompatibility, minimum gelation concentration, thixotropicity, and above all, precise and comparatively high drug payload. In the above-mentioned tripeptide systems, although various aromatic capping groups were employed, it was found that the thixotropy behavior is independent of the capped group. Phe/PhePhe plays a vital role in designing tripeptide-based thixotropic hydrogels, as can be observed from the literature mentioned above. Among the reported tripeptide thixotropic hydrogels, T_3_P5 showed good recovery, but the gelation medium (0.5 Mol NaOH) can be one of the limitations for biomedical applications, whereas the hydrogelator T_3_P10 showed the best recovery and self-healing properties. Out of these tripeptide gelators, the T_3_P16 gelator is the best in terms of both thixotropy and biological applications.

## 6. Tetrapeptide (T_4_P)-Based Thixotropic Hydrogel

**Figure 12:**
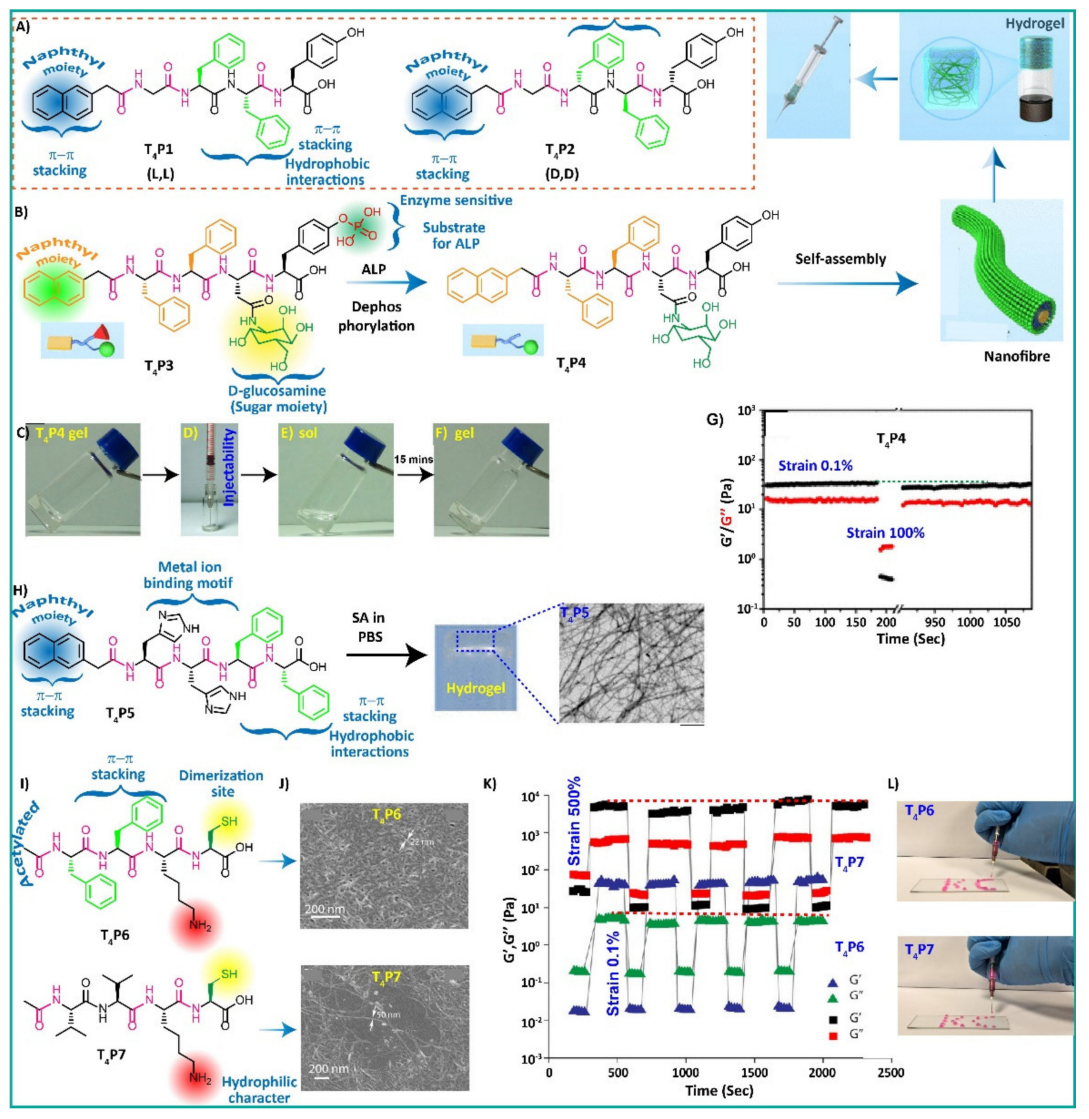
(**A**) Chemical structure of the tetrapeptide hydrogelators (T4P_1_ and T4P_2_); redrawn from Ref. [122]. Copyright is not applicable here. (**B**) Molecular structure of a glycopeptide with self-assembly schematic to form an injectable hydrogel. (**C**–**F**) Injectability and self-healing photographs of the hydrogel. (**G**)Thixotropy test of the T_4_P4 hydrogel; reproduced with permission from Ref. [123]. Copyright (2018) American Chemical Society. (**H**) Chemical structure and an inverted vial containing the hydrogel and TEM image; reproduced with permission from Ref. [124]. Copyright (2018) American Chemical Society. (**I**) Molecular structure. (**J**) SEM images. (**K**) Rheology experiment confirming the thixotropic behavior. (**L**) Injectability behavior of the tetrapeptide hydrogelator; reproduced with permission from Ref. [103]. Copyright (2020) American Chemical Society.

LMW hydrogels have attracted considerable attention in biomedical sciences as an ideal biomaterial because of their high porosity with a large amount of entrapped water and inherent biocompatibility, such as human tissue [120,121]. Tetrapeptides are also universally considered as LMWGs. In 2017, Yang, Gong, Li, and co-workers reported naphthyl acetic acid-capped tetrapeptides (T_4_P1 and T_4_P2, Figure 12A) composed of Gly, PhePhe dipeptide, and Tyr [122]. The peptide forms a hydrogel in PBS buffer at pH 7.4 with thixotropic behavior with injectable properties (Table 5). The D-enantiomeric gel (T_4_P2) also exhibited thixotropic properties. Furthermore, Li and co-workers modified the above-mentioned tetrapeptide sequence in which phosphate-protected Tyr was used for enzyme sensitivity, and Asp was functionalized with D-glucosamine (T_4_P3, Figure 12B) [123]. The peptide T_4_P3 undergoes alkaline phosphatase (ALP)-induced self-assembly through hydrogen bonding and π-π stacking to form an injectable supramolecular transparent thixotropic hydrogel (Figure 12C) under physiological conditions with 85% recovery of their original one (Figure 12C–G, Table 5). When the gel was extruded through a 0.4 mm diameter of the needle (Figure 12D), the sample transformed into a stable gel within 15 min, confirming the injectable property (Figure 12F). In the same year, Huang et al. reported the same naphthyl acetic-capped tetrapeptide (T_4_P5), comprising PhePhe with high self-assemble propensity. Di-His for metal ion binding in proteins undergoes self-assembly in PBS to form thixotropic-transparent hydrogels with a nanofibrous network (Figure 12I, Table 5) [124].

Inspired by the LysCys peptide hydrogelator series [40,82] in 2020, our group demonstrated a library of unprotected or acetyl-protected tetrapeptides composed of GlyGly, AlaAla, PhePhe, LeuLeu, IleIle, ValVal at the N-terminal and a common LysCys dipeptide unit at the C-terminal experience self-assembly in Tris buffer at pH 8 through dimerization of a sulfhydryl group, H-binding, and hydrophobic interactions [103]. Only acetylated PhePhe (T_4_P6) and ValVal (T_4_P7) form self-supporting thixotropic hydrogels with fibrous networks (Figure 12K,L). π-π stacking interaction plays an essential role in the case of T_4_P6. The injectability nature of these hydrogels is shown in Figure 12L.

**Figure 13:**
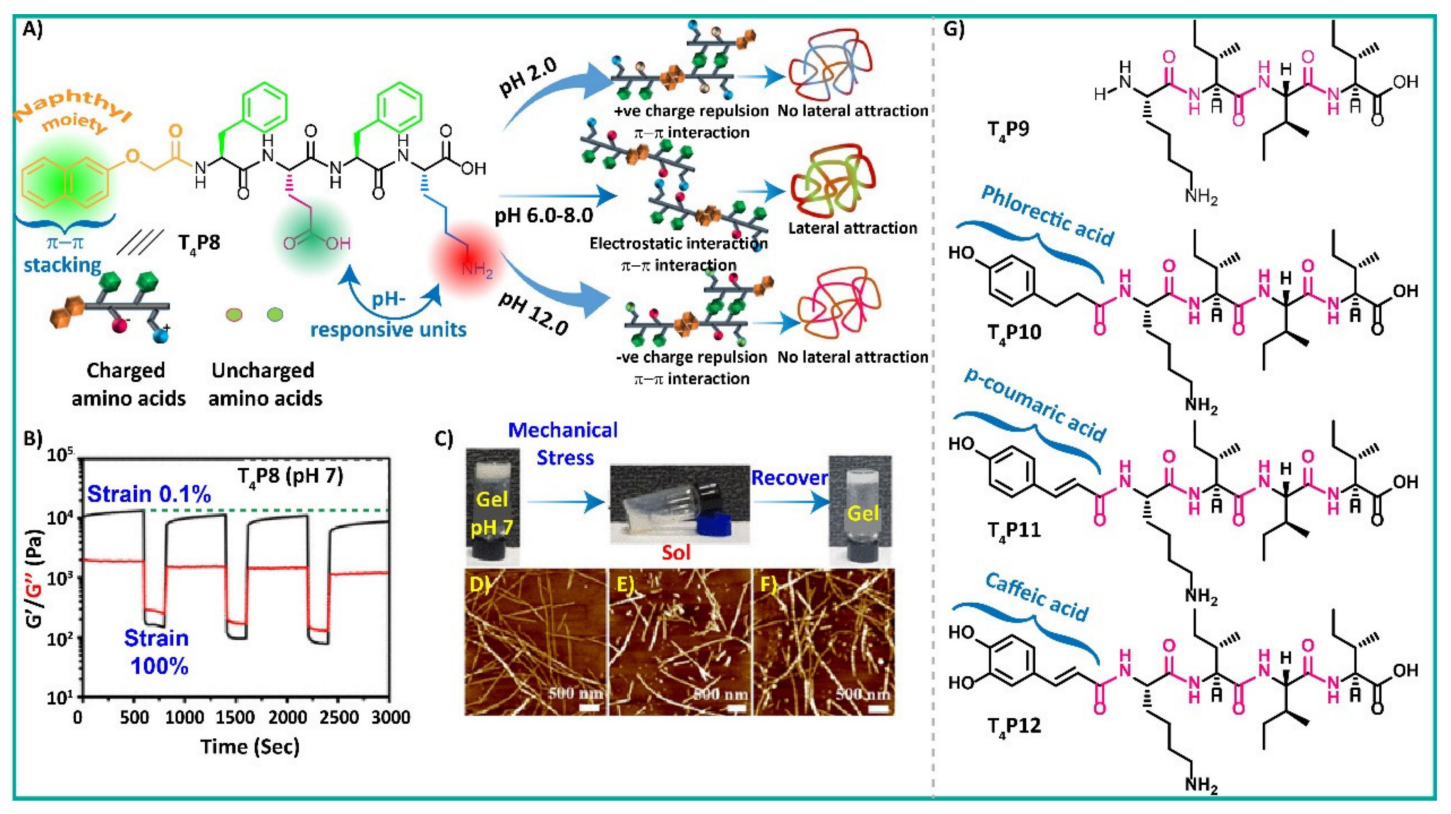
(**A**) Chemical structure of the tetrapeptide hydrogelator (T_4_P8) and possible self-assembly schematic illustration showing formation of different nanoarchitectures at different pH. (**B**) Thixotropy test of the tetrapeptide hydrogel at pH 7.0. (**C**) Stimuli-responsive gel-sol-gel transition of T4P8. (**D**–**F**) AFM images of the stimuli-responsive gel-sol-gel state; reproduced with permission from Ref. [125]. Copyright (2020) American Chemical Society. (**G**) Molecular structure of the tetrapeptide hydrogelators. The structures were redrawn from Ref. [126].

In the same year, Roy’s group anticipated a tetrapeptide, T_4_P8 (Figure 13A), made of alternating hydrophobic (Phe) and hydrophilic (-Lys (positive), -Glu (negative)) amino acids, and the N-terminal of the peptide is capped with a Nap group [125]. The tetrapeptide forms a stable, self-supporting supramolecular hydrogel over a broad pH range of 2.0–12.0 (Table 5). The gel formed at pH 7.0 was employed to measure the thixotropy of the gel (Figure 13B). The gel lost its three-dimensional network to form a quasi-liquid state (G′ < G″) at a higher applied strain (γ = 100%), and when the strain was lowered to the initial value (γ = 0.1%), reconstruction of the gel network occurred (G′ > G″) with 70% recovery of the initial moduli, indicating a modest self-healing behavior. The group presented the thixotropic behavior both optically and microscopically, as shown in Figure 13C–F). Next, Dewangan and colleagues reported a series of aromatic-capped (phloretic acid, p-coumaric acid, and caffeic acid) conjugated tetrapeptides (T_4_P9, T_4_P10, T_4_P11 and T_4_P12) by employing an amyloidal protein-originated LysLeuIleIle peptide motif [126]. Out of these peptides, T_4_P10 forms a supramolecular self-supportive stiff nanofibrous hydrogel network through π-π stacking of an aromatic-capped group in addition to hydrophobic and H-bonding interactions. The thixotropy property of the gel was checked mechanically (Table 5). When the gel was forcefully shaken by hand, the gel immediately broke down to form a clear solution, but upon standing for 1 h, the solution immediately recovered its gel character, indicating injectability behavior.

This section explores a diversity of amino acids with different chemical facilities to prepare tetrapeptide hydrogelators, which undergo self-assembly through different noncovalent interactions to form supramolecular nanostructures with thixotropic behavior. Therefore, these peptide hydrogels can be a suitable biomaterial for tissue engineering applications. The hydrogelator reported by Roy et al. showed a wide range of pH tuning with self-recovery behavior. Therefore, the gel may be an ideal candidate to entrap cargoes that can be released at the site of interest.

## 7. Co-Assembled Thixotropic Hydrogel

Co-assembly is a special type of self-assembly process where more than one molecule participates in creating supramolecular nanoarchitectures [127,128,129,130]. In recent years, supramolecular co-assembled gels, i.e., gels made of two or more molecules, have gained enormous attention in supramolecular chemistry and biomedical sciences due to their tunability in physical properties [131,132].

**Figure 14:**
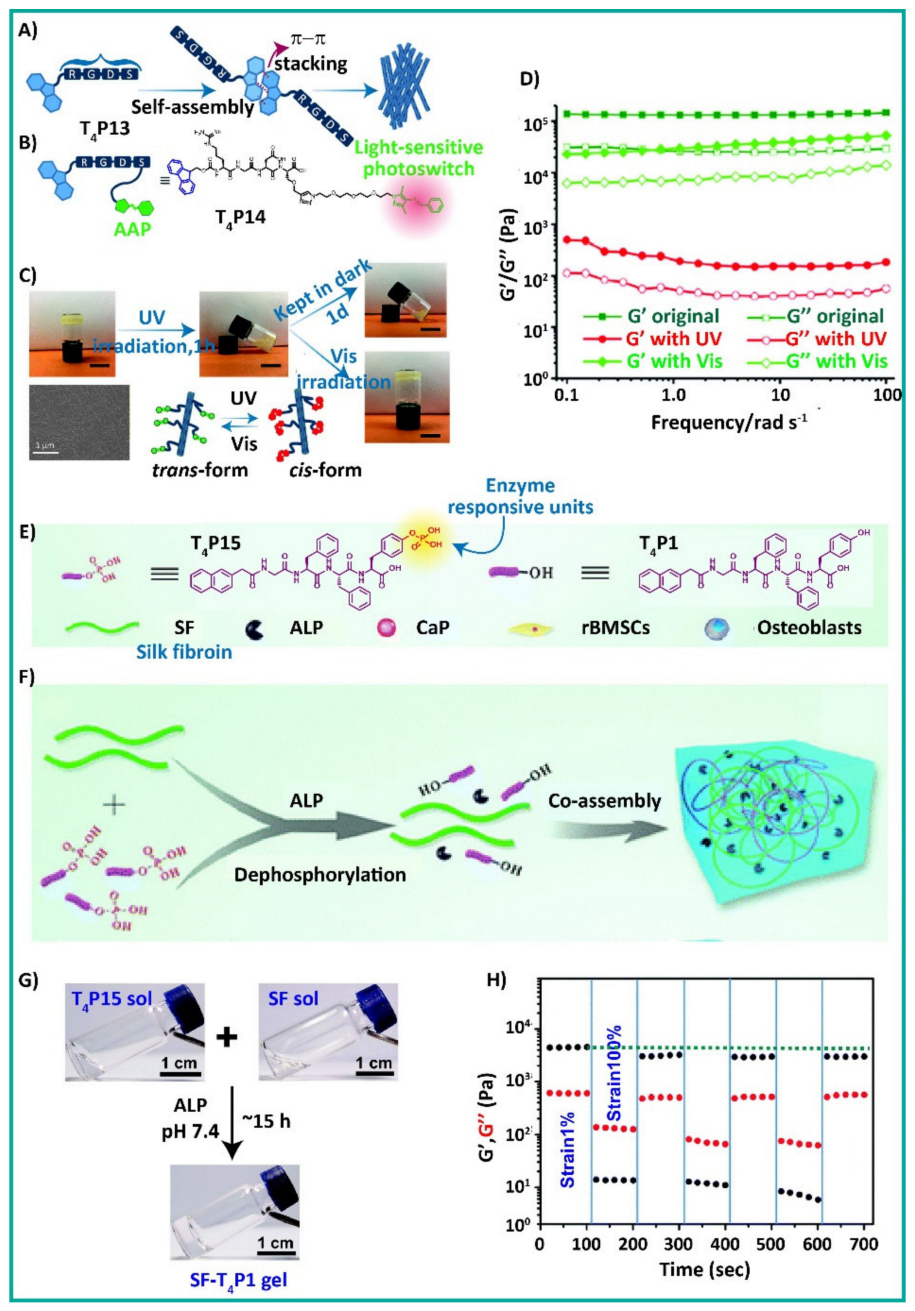
(**A**,**B**) Chemical structure and schematic illustration of the self-assembly of the hydrogelators (T_4_P13 and T_4_P14). (**C**) Reversible photo-responsive experiment between *trans*- and *cis*-isomerization of an AAP unit, possible schematics and SEM images of the resultant gel. (**D**) Thixotropy test of the resultant hydrogel; reproduced with permission from Ref. [138]. Copyright (2017) Royal Society of Chemistry. (**E**) Chemical structures of the co-assembled precursors. (**F**) Pictorial representation of co-assembly process to form the hydrogel. (**G**) Digital images of solution and gel containing vials. (**H**) Shear-recovery cycle of the resulted co-assembled hydrogel; reproduced with permission from Ref. [149]. Copyright (2020) Royal Society of Chemistry.

Host–guest chemistry is one of the leading techniques to create a supramolecular peptide hydrogel [133,134,135,136,137]. The peptide sequence, Fmoc-ArgGlyAspSer (T_4_P13), was chosen by Ravoo’s group owing to its good water solubility and high biocompatibility nature [138]. In addition, ArgGlyAspSer is a well-known cell adhesive peptide motif used for many biological applications [56,139]. To explore the stimuli responsiveness, a light-responsive group, arylazopyrazole (AAP) [140,141], was introduced to the side chain of Ser because of the photo-isomerization property as well as a high binding affinity toward beta-cyclodextrin (*β*-CD) [142,143] through host–guest chemistry. In advanced studies, compared to orthodox CD-decorated polymers, cyclodextrin vesicles (CDVs) [144,145] are generally used to create supramolecular polymers because of non-covalent cross-linkers. Among the available stimuli, light as a trigger is worthy of mention due to the specific targeting ability of the gel with high resolution in space and time and in tandem with its non-invasive nature [146,147,148]. On account of this, a light-responsive supramolecular self-supporting hydrogel was established by the mingling of Fmoc-ArgGlyAspSer (T_4_P13, 25%, 38 mM), Fmoc-ArgGlyAspSer-AAP (T_4_P14, 0.25%, 2.3 mM), CDPs (50 μM), which undergoes self-assembly through π-π stacking between Fmoc units, H-bonding as well as this host–guest interaction to form entangled nanofibers (Figure 14A–C, Table 6) [138]. Under UV light irradiation, the gel experiences a weak self-supporting nature because of the loss of host–guest supramolecular interaction. This can be explained in terms of isomerization of *trans*-AAP to *cis*-AAP, which cannot bind with *β*-CD, and as a consequence, weak noncovalent interaction within the network was observed (Figure 14C). As expected, the gel recovered its initial rheological properties upon resting at dark light or visible light irradiation, although the gel failed to recover the initial moduli value. The rheological data also support the moderate thixotropic nature of the gelator (Figure 14D).

Enzymes are another well-known biocompatible external stimulus utilized to prepare hydrogels from representative precursors or substrates via molecular transformations [152,153]. For instance, Li et al. demonstrated the co-assembled hydrogel from a well-recognized phosphorylated peptide sequence, Nap-GlyPhePheTyr (H_2_PO_3_) (T_4_P15), and a fibrous protein, silk fibroin (SF) [149,154] (Figure 14E–G). SF hydrogels are remarkable candidates for tissue engineering applications due to similar fibrous structures to native ECM, tuneful porosity, and high water content, and above all, they allow for easy encapsulation of cells, enzymes, and cargoes of interest [155]. Additionally, SF exhibited good biocompatibility, low immunogenicity, slow biodegradability, and notable strength and toughness, a primary concern of biomedical applications [149]. The peptide precursor undergoes enzymatic catalysis in the presence of ALP [156,157], resulting in the formation of self-assembled molecules that later develop into a self-supporting hydrogel (Figure 14E,F; Table 6). ALP removes the hydrophilic phosphate groups from the peptide precursors to create Nap-GlyPhePheTyr (T_4_P1), thereby tweaking the balance of multiple noncovalent interactions such as H-bonding, hydrophobic interactions, and electrostatic interactions to activate the self-assembly, which eventually leads to a 1D nanofibrillar followed by gelation of the ensuing product (Figure 14F). During this process, the self-assembled peptide synergistically induces the secondary conformation of SF from a random coil to a stable *β*-sheet-ordered arrangement, and as a result, a stable hydrogel (SF-T_4_P1) is formed. The resultant gel recovered around 66% of the initial G′ value because of the dynamic non-covalent interactions between SF and the dephosphorylated peptide unit, indicating self-healing propensity under physiological conditions (Figure 14H).

**Figure 15:**
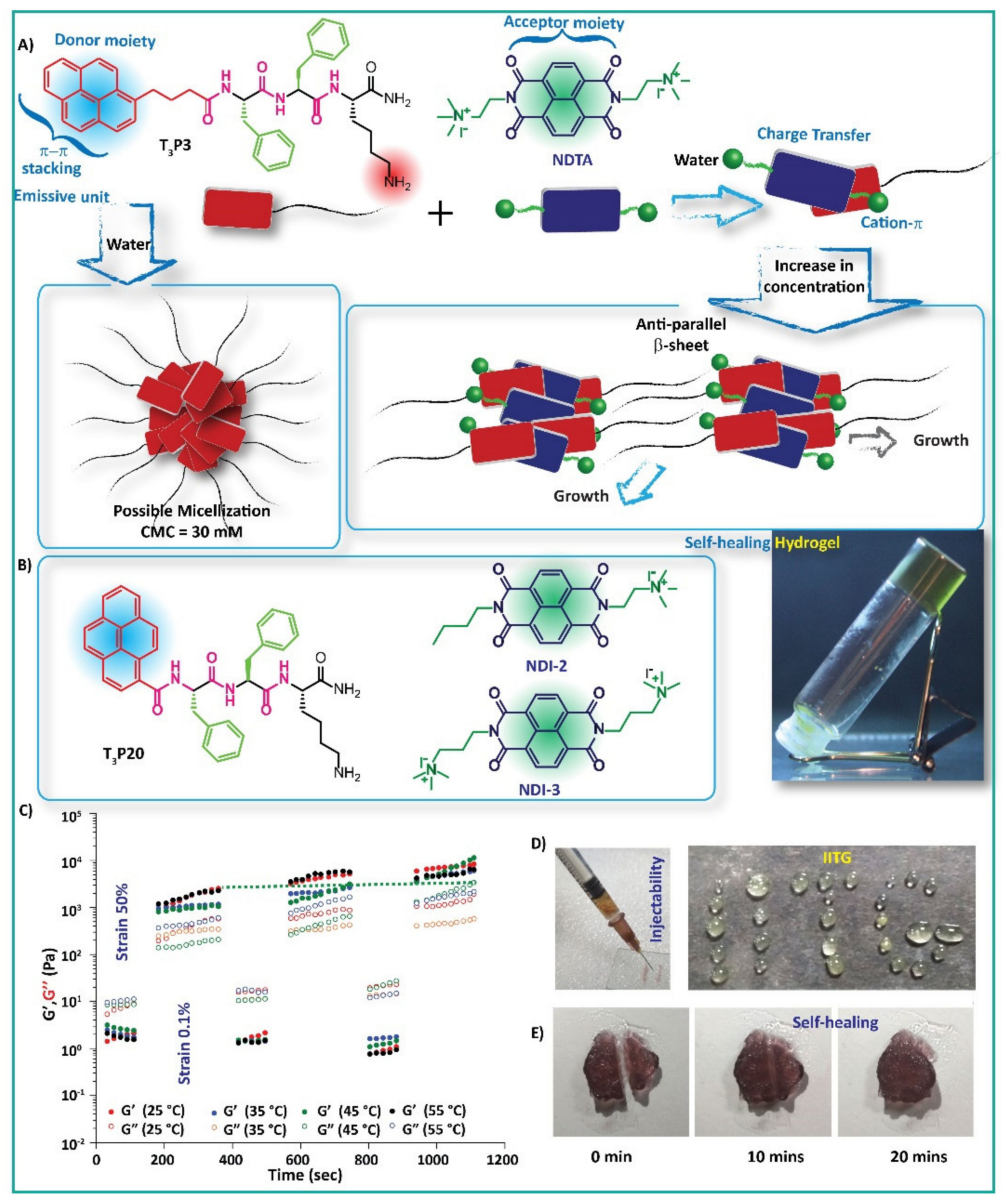
(**A**,**B**) Chemical structure of the pyrene-protected tripeptides (T_3_P3 and T_3_P20) and NDI derivatives (NDTA, NDI-2 and NDI-3).; schematic illustration of the self-assembly of T_3_P3 and co-assembly with NDTA; an inverted vial showing the co-assembled hydrogel. (**C**) Thixotropy test of the co-assembled hydrogel at different temperature. (**D**) Injectable behavior. (**E**) Macroscopic self-healing property of the co-assembled hydrogel; reproduced with permission from Ref. [5]. Copyright (2019) American Chemical Society.

Charge-transfer (CT) interaction is a special kind of noncovalent interaction between an electron-rich donor molecule and an electron-deficient acceptor molecule, which has shown its potential to develop soft biomaterials [158,159,160,161,162]. The interaction differs from the conventional supramolecular noncovalent forces such as H-bonding, hydrophobic interactions, and π-π stacking. In addition to the CT interaction, cation–π interaction is another critical noncovalent interaction recognized in biological systems to stabilize the tertiary or quaternary structures in proteins [5,163]. Taking together an unorthodox combination of the above-mentioned interactions, in 2019, our group explored the co-assembly of pyrene-capped tripeptides (Py-PhePheLys:T_3_P3 (Py = 1 pyrene butyric acid) and T_3_P20 (Py = 1 pyrene carboxylic acid); Figure 15A,B), which undergo self-assembly with cationic naphthalene diimide (NDI) through CT interactions between the donor (pyrene) and acceptor (NDI) molecules (Figure 15A,B), cation-π interaction between pyrene and quaternary ammonium group from NDI, and *β*-sheet arrangement of the PhePheLys unit to form a self-supporting thixotropic hydrogel [5] (Figure 15A, Table 6). The G′ value was found to be increasing in the second and third cycle of the time-dependent step-strain cycle. This might be because of the formation of a more stable gel network after the first deformation step, substantiating a fast self-healing ability of the gel. Additionally, the gels exhibited excellent self-healing properties at elevated temperatures (Figure 15C). When the gel was extruded through a syringe, the extruded liquid immediately transformed into a gel, indicating an injectable nature (Figure 15D). Furthermore, the self-healing property was explored macroscopically. A piece of hydrogel was cut into two parts with the help of a surgical blade and put together. The gel self-healed completely after 20 min (Figure 15E).

**Figure 16:**
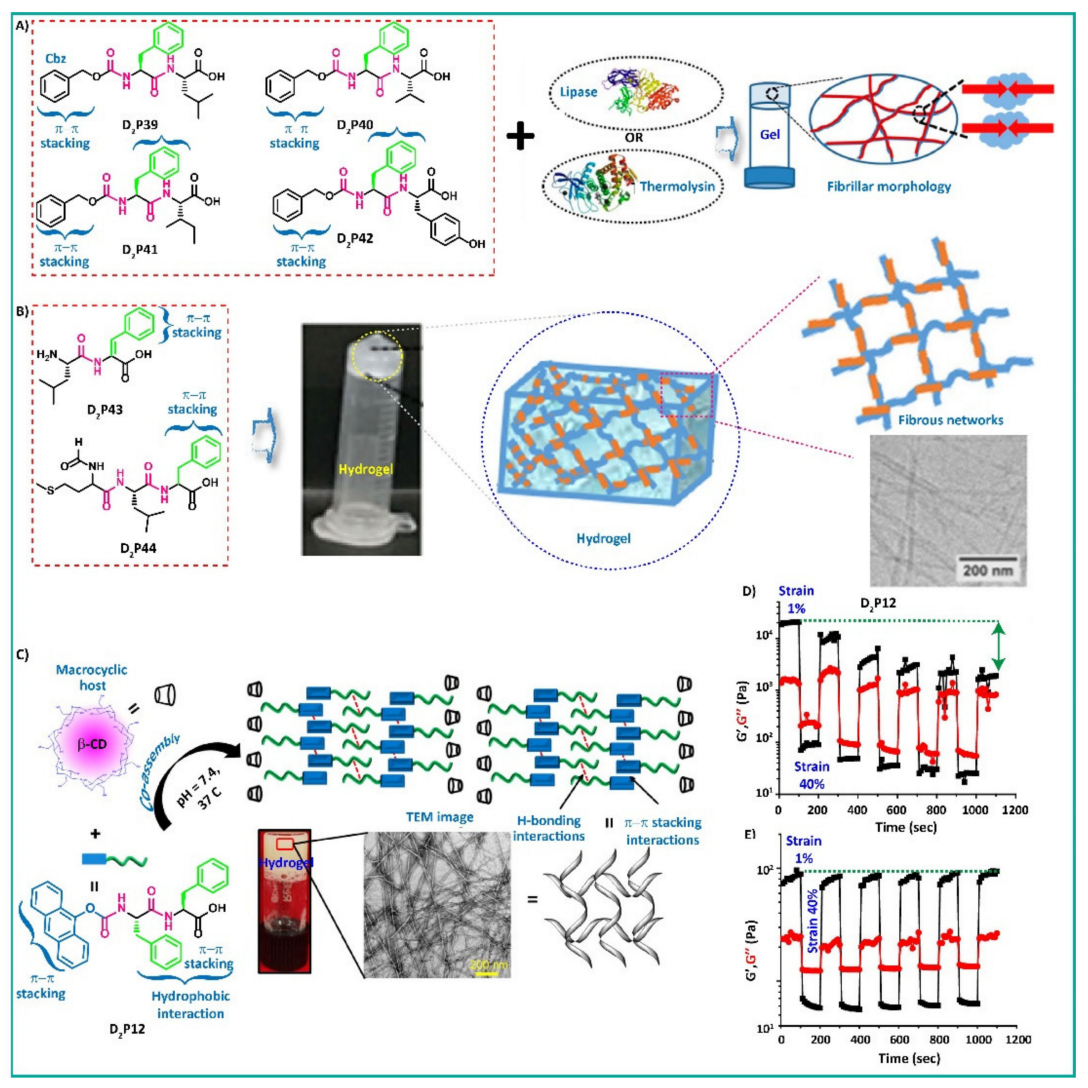
(**A**) Chemical structure of the co-assembled hydrogelators (D_2_P39-D_2_P42); reproduced with permission from Ref. [17]. Copyright (2020) American Chemical Society. (**B**) Molecular structure, gel photograph, pictorial fibrous networks and SEM images of co-assembled hydrogels between D_2_P43 and D_2_P44 [150]. No copyright is needed, as this is an Open Access article under the terms of the Creative Commons Attribution License. (**C**) Molecular structure of β-CD, D_2_P12 hydrogelator; schematic co-assembly between β-CD and D_2_P12 hydrogelator; an inverted vial and TEM image of the resulted hydrogel. (**D**,**E**) Thixotropy test of D_2_P12 and co-assembled hydrogel; reproduced with permission from Ref. [151]. Copyright (2020) American Chemical Society.

**Figure 17:**
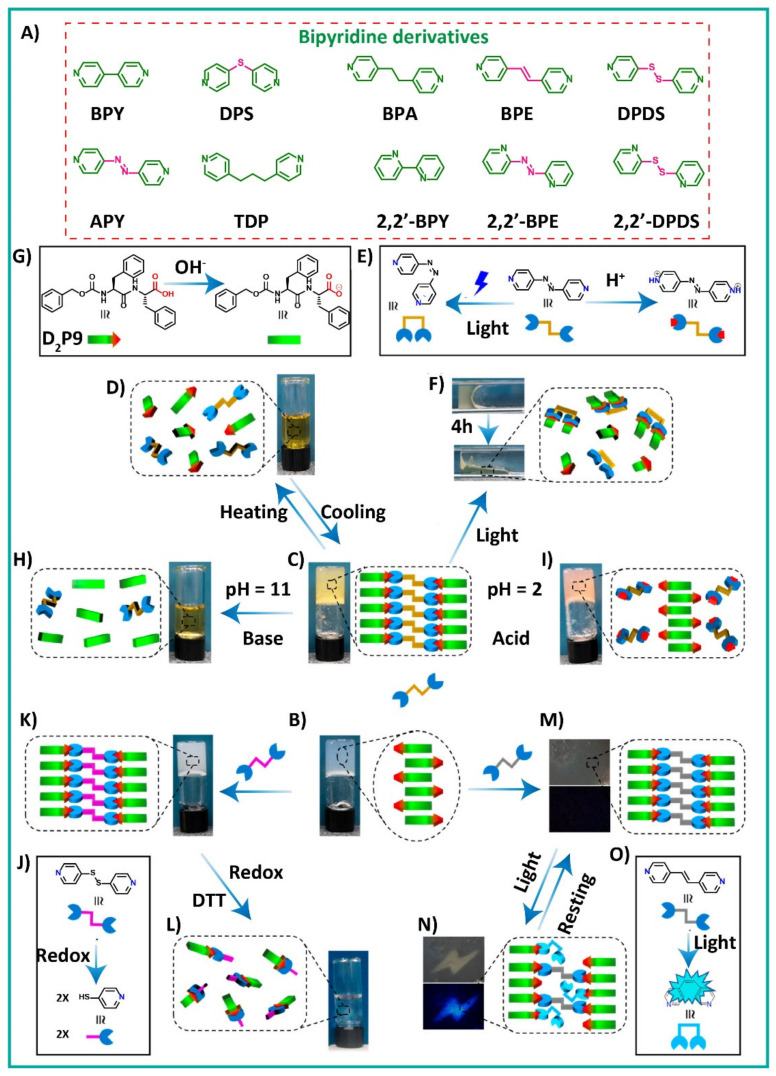
(**A**) Molecular structures of bipyridine derivatives; redrawn from Ref. [98]. Copyright is not applicable here. (**B**–**O**) Multiple-responsive (pH, redox reagents, light, heat, acid and base) co-assembled hydrogels produced between D_2_P16 and bipyridine derivatives; reproduced with permission from Ref. [98]. Copyright (2021) American Chemical Society.

Following, Roy et al. reported a series of PhePhe dipeptide analogs in which different amino acids replaced C-terminal Phe with different polarities such as Leu, Val, Ile, and Tyr, and the N-terminal was capped by the Cbz group [17] (Figure 16A). The dipeptides are unable to form a gel under experimental conditions, but in the presence of hydrolytic enzymes (here, lipase or thermolysin), the nongelator molecules undergo self-assembly to form a thixotropic hydrogel through protein–peptide interactions (Table 6). Protein–peptide interactions are omnipresent in nature and play a vital role in various cellular processes, and the imbalance of the interaction causes several human diseases [17]. In addition, the interaction holds great promise to enhance the efficacy of peptide therapeutics. The developed hydrogel loses its gel character upon vertexing and is transformed into a sol state. Upon resting, the gel state is regained, confirming the thixotropic behavior of the gel. The thixotropic properties are independent of the nature of the proteins used. Ironically, all the resultant hydrogels can recover almost entirely in a period of the first 60 secs, confirming the injectable behavior for biomedical applications. Later, in the same year, Koksch et al. introduced a nonproteinogenic amino acid, α,β-dehydrophenylalanine (ΔPhe), containing a dipeptide hydrogelator (LeuΔPhe, Figure 16B), which experiences co-assembly with N-formyl-MetLeuPhe in 0.8 Mol sodium acetate buffer through intermolecular π-π stacking between the dehydrophenyl groups (LeuΔPhe) and a phenyl (f-MLF) unit to generate a self-supporting injectable hydrogel (Table 6) [150]. The presence of ΔPhe triggers hydrogel formation in the ultrashort peptide sequence. In this direction, Das et al. demonstrated the co-assembly of an Amoc-capped dipeptide, Amoc-PhePhe [151]. The peptide itself undergoes self-assembly in PBS (pH 7.4, 37 °C) to form a thixotropic hydrogel, but the gel lacks injectability. Therefore, to bring the injectability naturally, the hydrogelator was co-assembled with the help of *β*-CD. The presence of CD greatly affects the physical properties of the hydrogel. The consequent co-assembled hydrogel (Figure 16C, Table 6) displayed nearly 95% recovery compared to pure hydrogel as confirmed from the periodical low (γ = 1.0%)–high (γ = 40%)–low (γ = 1.0%) step-strain rheological experiment (Figure 16C,D). This can be attributed to noncovalent interactions such as H-bonding in the co-assembled state. In a similar attempt to generate a co-assembled hydrogel, Gazit and co-workers also reported an N-terminal-capped PhePhe motif, in particular, Cbz-protected peptide, which undergoes self-assembly with different bipyridine derivatives, namely BPY, DPS, BPA, DPDS, TDP, 2,2′-BPY, 2,2′-BPE (Figure 17A) to form self-supporting thixotropic hydrogels via strong intermolecular H-bonding between a free –COOH group at the C-terminal of the peptide sequence and the pyridine N [98]. The co-assembled hydrogels are mechanically weak in comparison to pure hydrogel and can be explained in terms of a more ordered arrangement of nanofibers in the pure gel. The co-assembled hydrogels offer mechanoresponsive gel-sol-gel transition, an indication of thixotropicity. In the same perspective, Shanmugam et al. reported 4,4′-azopryidine (APy)-induced supramolecular thixotropic hydrogels utilizing well-known LMW-based gelators, Fmoc-Phe and Fmoc-Tyr [164]. Due to the presence of two pyridyl moieties at the end of the azo group, Apy forms strong H-bonding with the carboxylic acid (–COOH) group present in Fmoc-Phe/Fmoc-Tyr molecules in a single as well as in two-component gel systems. It was found that both G′ and G″ almost fully recovered their initial value after the end of the periodical step-strain cycle, confirming the self-healing nature of the hydrogels. Out of these hydrogels, the recovery rate of the FmPhe:Apy hydrogel was uninterruptedly reduced after the end of each cycle, implying the low self-healing ability of the gel.

In this section, co-assembled thixotropic hydrogels were discussed in which charge transfer interactions, H-bonding, and host-guest interactions play vital roles in the self-assembly process. Charge-transfer and a cation-π-induced thixotropic hydrogel reported by Das et al. appeared to have good recovery of the rheological properties even at elevated temperature with excellent self-healing ability. Host-guest-induced co-assembled hydrogels are also in the same queue for their outstanding self-recovery ability.

## 8. Thixotropic Composite Hydrogel

**Figure 18:**
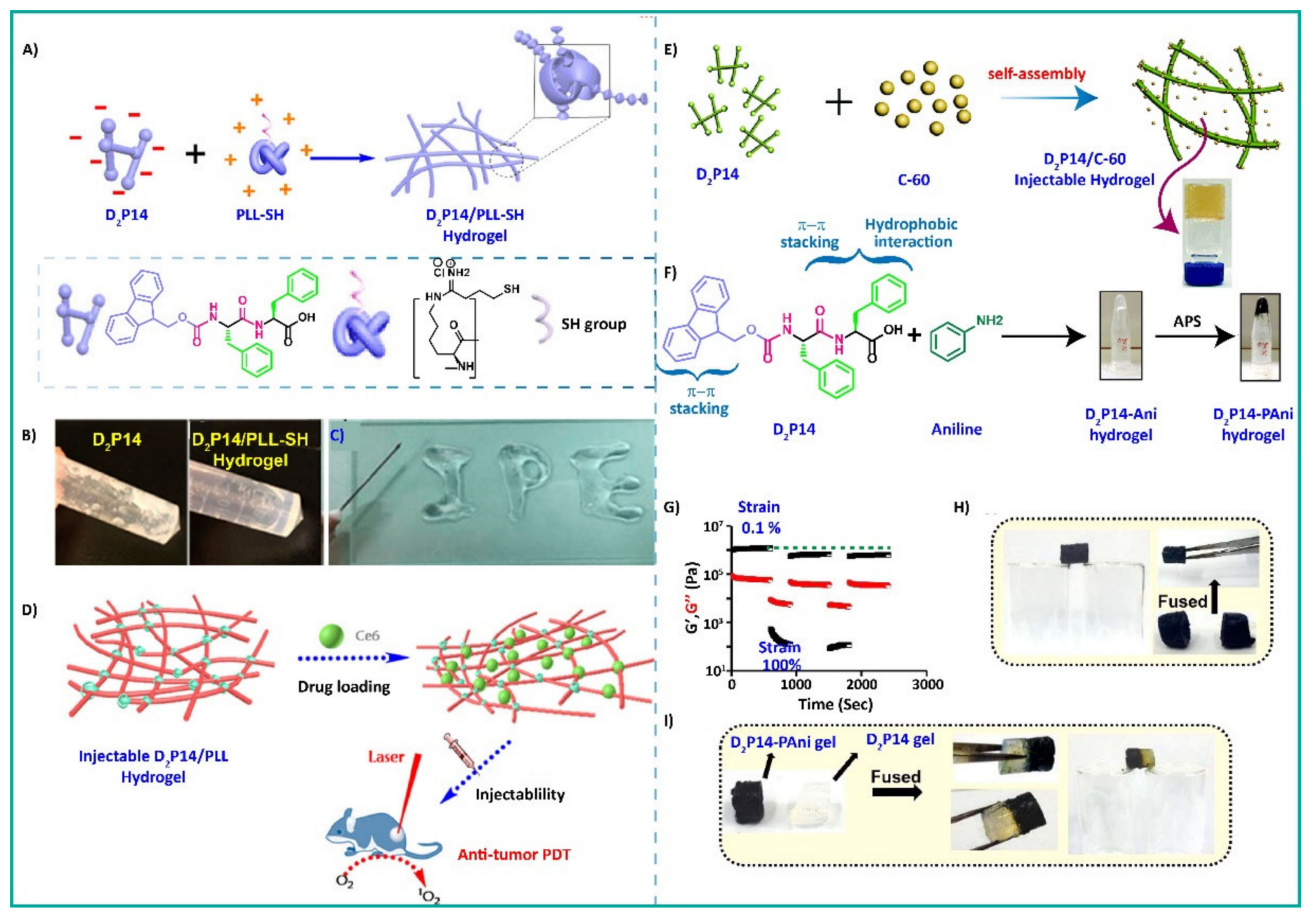
(**A**) Schematic illustration of the D_2_P14/PLL-SH composite hydrogel. (**B**) D_2_P14 hydrogel showing separation of water and gel; D_2_P14/PLL-SH hydrogel. (**C**) Injectability behavior of the D_2_P14/PLL-SH hydrogel; reproduced with permission from Ref. [171]. Copyright (2017) American Chemical Society. (**D**) Schematic representation of injectable D_2_P14/PLL hydrogels for delivery of Ce6 and anti-tumor PDT; reproduced with permission from Ref. [172]. Copyright (2018) American Chemical Society. (**E**) Schematic sketch of D_2_P14/PLL-SH injectable hydrogel with vial inverted photograph; reproduced with permission from Ref. [173]. Copyright (2018) Royal Society of Chemistry. (**F**) Chemical structure, photographs of D_2_P14/PAni composite hydrogel. (**G**) Rheology experiment confirming the thixotropic behavior of D_2_P14/PAni. (**H**,**I**) Self-healing behavior of D_2_P14 and D_2_P14/PAni hydrogels; reproduced with permission from Ref. [174]. Copyright (2019) American Chemical Society.

Composite hydrogels made of peptide building blocks and biocompatible polymers have been at the helm of advanced research for the last few decades [165,166,167]. It is well-documented in the literature that Fmoc-Phe-Phe (D_2_P14) can undergo self-assembly under physiological conditions to form well-organized fibrous hydrogels by a chronological change in pH [35,67,168]. At pH > 3.5, deprotonation of a free acid (–COOH, pKa~3.5) group resulted in electrostatic repulsion between the anionic Fmoc-PhePhe unit, which inhibited the formation of a self-supporting gel. However, at pH 7.8, the –COOH group remains as an ionizable form, whereas the free amino (–NH_2_, pKa~8.8) group becomes protonated [167,169]. However, the mechanical properties of the hydrogel are not satisfactory for biomedical applications [170]. Therefore, to improve the mechanical properties of the hydrogel, in 2017, Yan’s group demonstrated a composite hydrogel composed of D_2_P14 and a well-known biocompatible cationic biomacromolecule, poly-L-lysine (PLL), which formed a hydrogel through electrostatic interactions with improvised mechanical properties [171] (Figure 18A,B, Table 7). The presence of a thiol unit (–SH) in PLL stabilizes the nanostructures through disulfide (–S–S–) bond formation. The composite hydrogel exhibited thixotropic properties with good recovery of their moduli. When the gel was injected through a needle of 26-gauge (φ = 260 μm), the solution state restored its gel state after 30 secs, indicating a self-healing ability (Figure 18C). The main reason for enhanced mechanical properties is the highly ordered supramolecular arrangement and high cross-linking between the self-assembled nanofibers induced by PLL. In advanced studies, the same group anticipated photodynamic antitumor therapy based on the previously reported injectable behavior of D_2_P14/PLL hydrogels (Figure 18D) [172]. Therefore, to reach their target, a photosensitive drug, Chlorin e6 (Ce6), was incorporated within the fibrous network, and the resultant hydrogel maintained the thixotropic and injectable properties. Paralleling this, the same group fabricated a hybrid hydrogel of D_2_P14 and a fullerene derivative, C60 pyrrolidine Tris–acid (C60-PTC), for photodynamic antibacterial therapy (Figure 18E, Table 7) [173]. The presence of C60-PTC induced the appearance of gel from transparent to yellowish. The formed hydrogel showed a better mechanical property than pure gel, which can be attributed to the nonvalent interactions between the peptides and nanoparticles. In addition, the hybrid gel showed a 92% recovery of its initial moduli value. In contrast, the pure peptide exhibited only 84%, confirming the superior mechanical behavior of the hybrid gel and making it a better candidate for injectable applications. Gazit and co-workers also employed a D_2_P14 dipeptide to prepare a composite hydrogel [174]. In this case, the D_2_P14 dipeptide was polymerized with aniline to form the gel by utilizing the solvent-switch procedure (Figure 18F). In brief, a concentrated DMSO solution of Fmoc-PhePhe was diluted with the help of an aqueous solution of aniline followed by oxidative polymerization by employing ammonium persulfate (APS) to yield a deep green-colored D_2_P14-PAni composite hydrogel with self-healing properties (Figure 18G, Table 7). When the blocks of the D_2_P14-PAni hydrogels were fused, within 5 min, one monolith of hydrogel was formed without any external agent, implying macroscopic self-healing behavior (Figure 18H). Similar macroscopic self-healing behavior was noted when two separate blocks of semitransparent D_2_P14 and dark D_2_P14-PAni hydrogels were fused, attributable to the excellent self-healing ability of D_2_P14 (Figure 18I). In line with the context, Adler-Abramovich et al. investigated a composite hydrogel with sodium alginate using the solvent-switch method while keeping the same peptide unit [175]. The gel was thixotropic, and the resulting gel recovered an average of 80% of its initial moduli.

**Figure 19:**
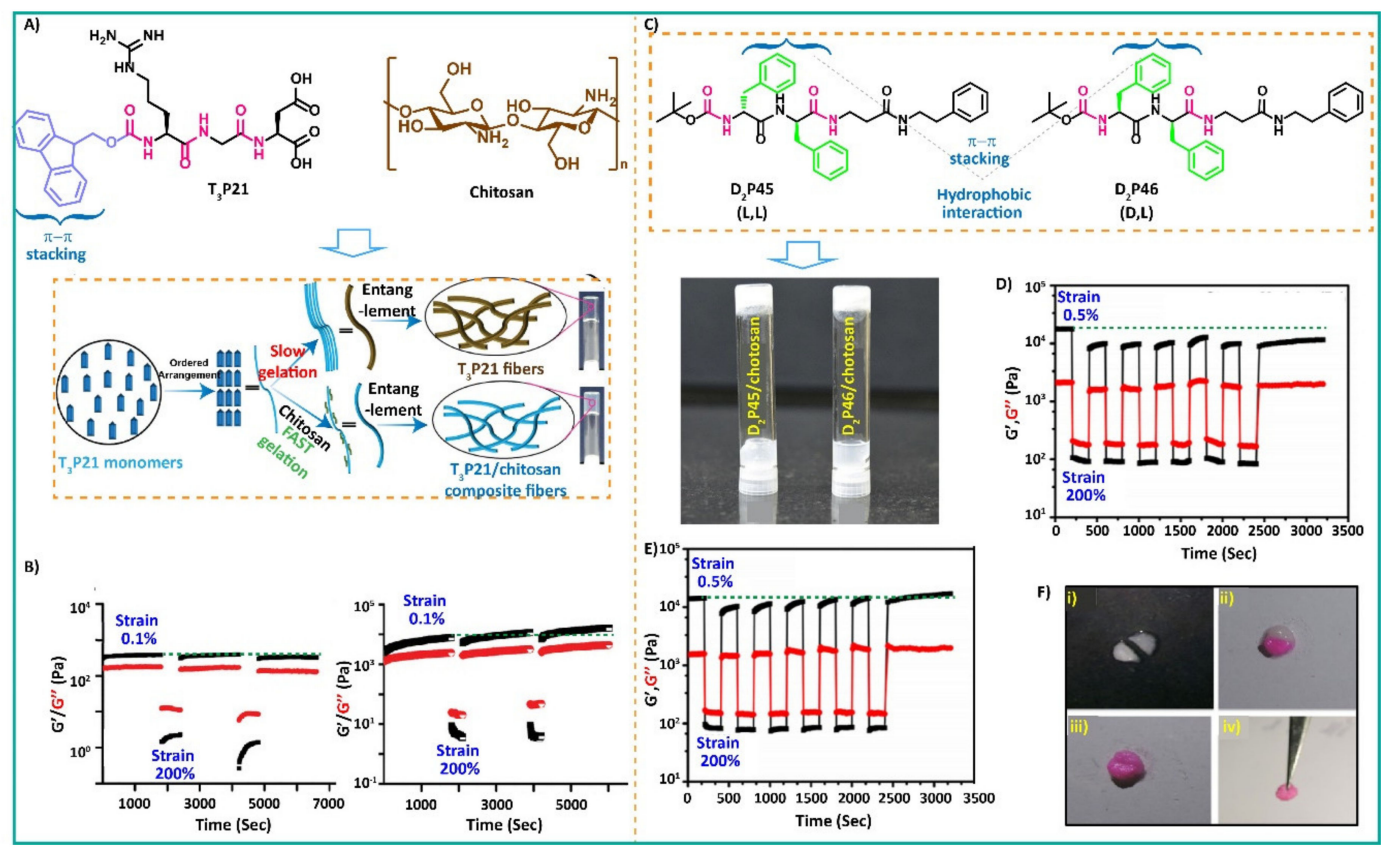
(**A**) Chemical structure of the tripeptide biopolymer, chitosan; schematic model of self-assembly process to form fibrous nanostructures and digital photographs of T_3_P20 and T_3_P20/Chitosan hydrogel. (**B**) Rheological step-strain analysis of T_3_P20 and T_3_P20/chitosan hydrogel; reproduced with permission from Ref. [176]. Copyright (2019) John Wiley and Sons. (**C**) Molecular structure of the dipeptides (D_2_P45 and D_2_P46) and digital photographs of D_2_P45-46/chitosan hydrogel. (**D**,**E**) Rheological thixotropic measurement of D_2_P45-46/chitosan hydrogel. (**F**) Self-healing characteristics of the gels; reproduced with permission from Ref. [178]. Copyright (2020) Elsevier.

Next, Gazit and co-workers anticipated another composite hydrogel with thixotropic properties [176]. However this time, the dipeptide was upgraded to a tripeptide, Fmoc-ArgGlyAsp (T_3_P21, Figure 19A), which undergoes self-assembly with a biodegradable, biocompatible, antibacterial biopolymer chitosan through H-bonding interactions with a free terminal carboxylic acid group of the tripeptide. The composite hydrogels exhibited 209% recovery of the moduli value, implying a stable hydrogel with high strength after the deformation, whereas pure hydrogel showed only 85% (Figure 19B, Table 7). This unique behavior is exciting and has hardly ever been noticed. In 2019, PVA and an Fmoc-capped tripeptide composite hydrogel was reported by Weiss et al. with thixotropic properties [177]. Singh et al. also fabricated chitosan-based composite gels by employing Boc-L-Phe-γ^4^-L-Phe-PEA (D_2_P45) and Boc-D-Phe-γ^4^-L-Phe-PEA (D_2_P46) and a peptide, which undergoes complexation with chitosan separately to form a self-supporting opaque gel [178] (Figure 19C, Table 7). At the end of the step-strain cycle, the gels recovered almost >90% of the initial value, confirming a promising self-healable nature (Figure 19D,E). The chitosan complexation may be responsible for such nice properties. Furthermore, the self-healing behavior was established macroscopically, as shown in Figure 19F. The prepared gels were cut into two halves, one of which was stained with rhodamine dye, and the other half was left intact, and the two halves were then stacked together. The dye diffused from the stained part to the untreated part, and the gel was healed after 36 h of incubation, owing to its self-healing ability.

**Figure 20:**
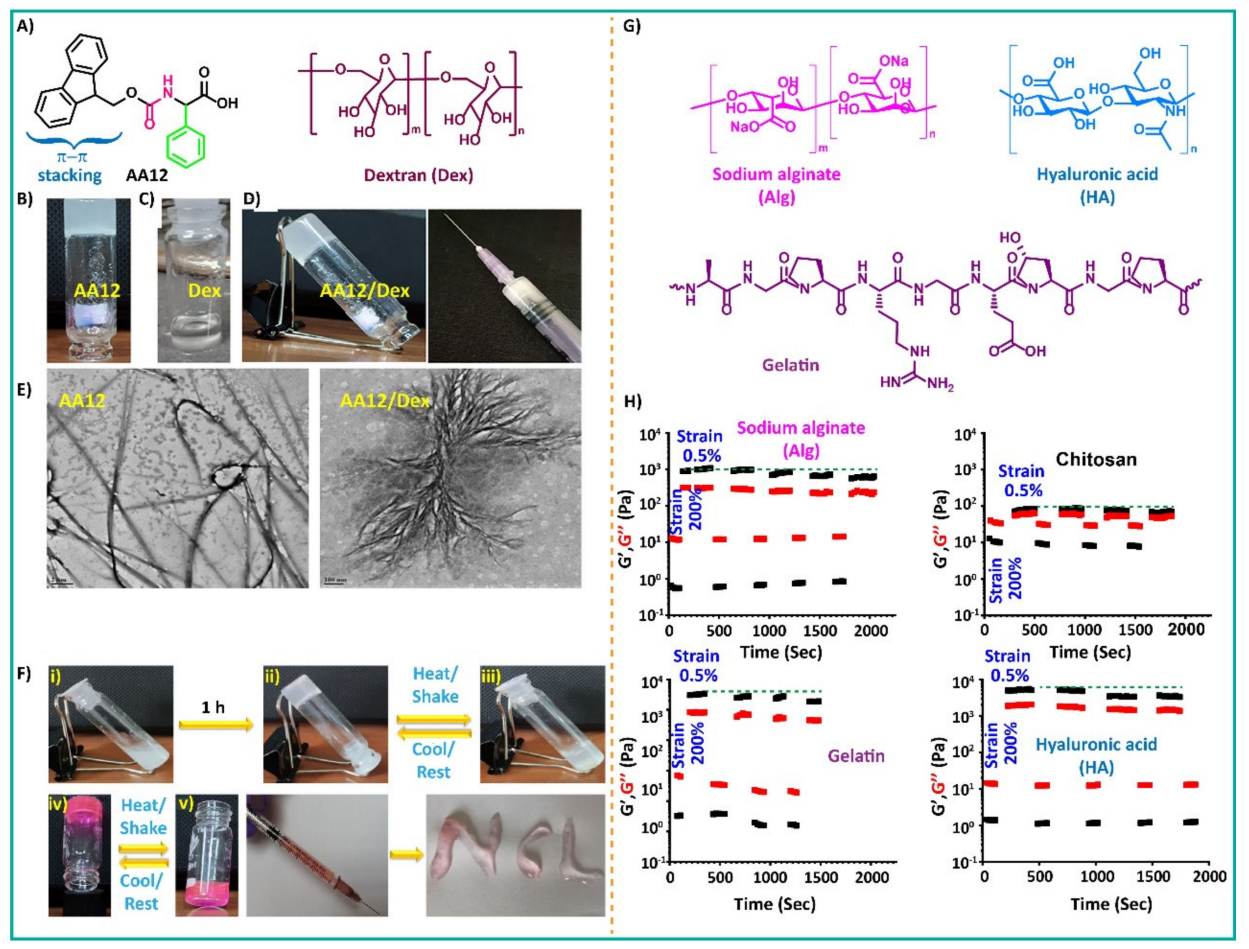
(**A**) Chemical structure of the Fmoc-capped amino acid (AA12) and dextran. (**B**) Photographs of inverted vial of AA12 hydrogel. (**C**) Detran solution and (**D**) AA12/dextran hydrogel in an inverted vial and syringe. (**E**) TEM images of AA12 and AA12/dextran hydrogel. (**F**) Stimuli-responsiveness and injectability nature of AA12/dextran hydrogel; reproduced with permission from Ref. [179]. Copyright (2021) John Wiley and Sons. (**G**) Molecular structure of the biopolymers. (**H**) Rheological step-strain measurement showing thixotropicity in the composite gels; reproduced with permission from Ref. [167]. Copyright (2021) MDPI AG; MDPI.

In this context, a polysaccharide-based composite hydrogel was reported by Abraham et al. in which Fmoc-Phe and non-toxic, hydrophilic, and bacterially derived dextran undergoes self-assembly in PBS buffer at pH 7.4 with a three-dimensional fiber network (Figure 20, Table 7) [179]. The presence of dextran improved the mechanical properties of the composite hydrogel compared to the pure gel of Fmoc-Phe. Both gels displayed thixotropic behavior, as confirmed by the rheological step-strain experiment. This is attributed to the supramolecular association through restructuring the fibrous networks. The recovered moduli value for composite hydrogel was found to be three times higher than pure gel, indicating good self-healing properties. The injectable nature of the composite hydrogel is shown in Figure 20. In 2021, Das et al. demonstrated two-component thixotropic composite hydrogels made of biopolymers such as gelatin, chitosan, hyaluronic acid, sodium alginate, and a self-assembling pyrene-capped dipeptide unit [167]. The presence of the dipeptide unit enhanced the mechanical stabilities of these composite hydrogels, as confirmed by the rheological experiment. This can be explained in terms of the tight woven network of the peptide molecule. At a higher strain (γ = 1000%), G′ was found to be lower than G″, indicating the demolishing of these composite hydrogels into a solution state, whereas at a lower strain (γ = 0.1%), the solution state restored its original gel state, and as a result, G′ became higher than G″, as can be seen from the time-dependent step-strain rheological experiment (Table 7). The experiment was continued for up to the fifth cycle, and no noticeable change was observed. The injectability nature of these hydrogels was also confirmed when the hydrogels were injected through a syringe in bulk water. Next, the mixture of Fmoc-PhePhe and glycol chitosan (GCS) was employed to form a self-supporting hybrid hydrogel in Tris-HCl buffer at pH 7.8 through electrostatic interaction by Yun’s group [169]. As expected, the thixotropic property endowed the gel to be simply injected through a syringe and then returned to its gelled stated after injection. Deng et al. also demonstrated the self-healing behavior of a composite hydrogel made of a series of aromatic-capped group (benzene, naphthalene, 2-anthracene, 9-anthracenemethoxycarbonyl, and 1-pyrene), with the most-studied being diphenylalanine (PhePhe) unit and a biocompatible natural polysaccharide, hyaluronic acid (HA) [180]. Out of these composite hydrogels, the N-PhePhe/HA gel showed better injectability and self-healing ability.

The development of composite materials for biomedical sciences is still demanding because of their fast gelation time, high mechanical stability, good self-healing abilities, injectable properties, high drug loading capacity, and above all, biological safety. Fmoc-PhePhe is the most studied self-assembled dipeptide gelator to create composite hydrogels with various biocompatible polymers such as chitosan, hyaluronic acid, sodium alginate, etc. The composite hydrogels of a pyrene-capped dipeptide, Py-LysCys with gelatin, chitosan, hyaluronic acid, and sodium alginate reported by Das et al. can be alternative choices to explore more owing to their stimuli-responsive dimerization unit.

## 9. Biological Applications

Due to their inherent biocompatibility, biodegradability, and morphological similarities to ECM’s fibrous proteins, self-assembled supramolecular peptide hydrogels have emerged as a leading class of biomaterials for biomedical sciences over the last few decades [25,80,123,179,181]. Among the peptide hydrogels, the hydrogels with injectable behavior have gained enormous attention in the field of drug delivery, wound dressings, cell encapsulation, etc., because of plentiful advantages, including in situ formability, easy incorporation of drugs/cells, tissue repair and regeneration, surgical fillers, and triflingly invasive delivery [25,28,76,81,85]. Many of these reported hydrogels showed their potential applications, such as a scaffold to mimic the extracellular matrix (ECM) for cell growth in a 2D/3D cell culture under physiological conditions [99], 3D cell culture for tumor spheroids and fibroblasts, and some of them are exploited for antibacterial activities [61,72] and enzyme storage [40,62]. In addition, some thixotropic hydrogels showed controlled delivery of their payloads, as conventional drug delivery systems always suffer burst release and repeated cargo administration.

**Figure 21:**
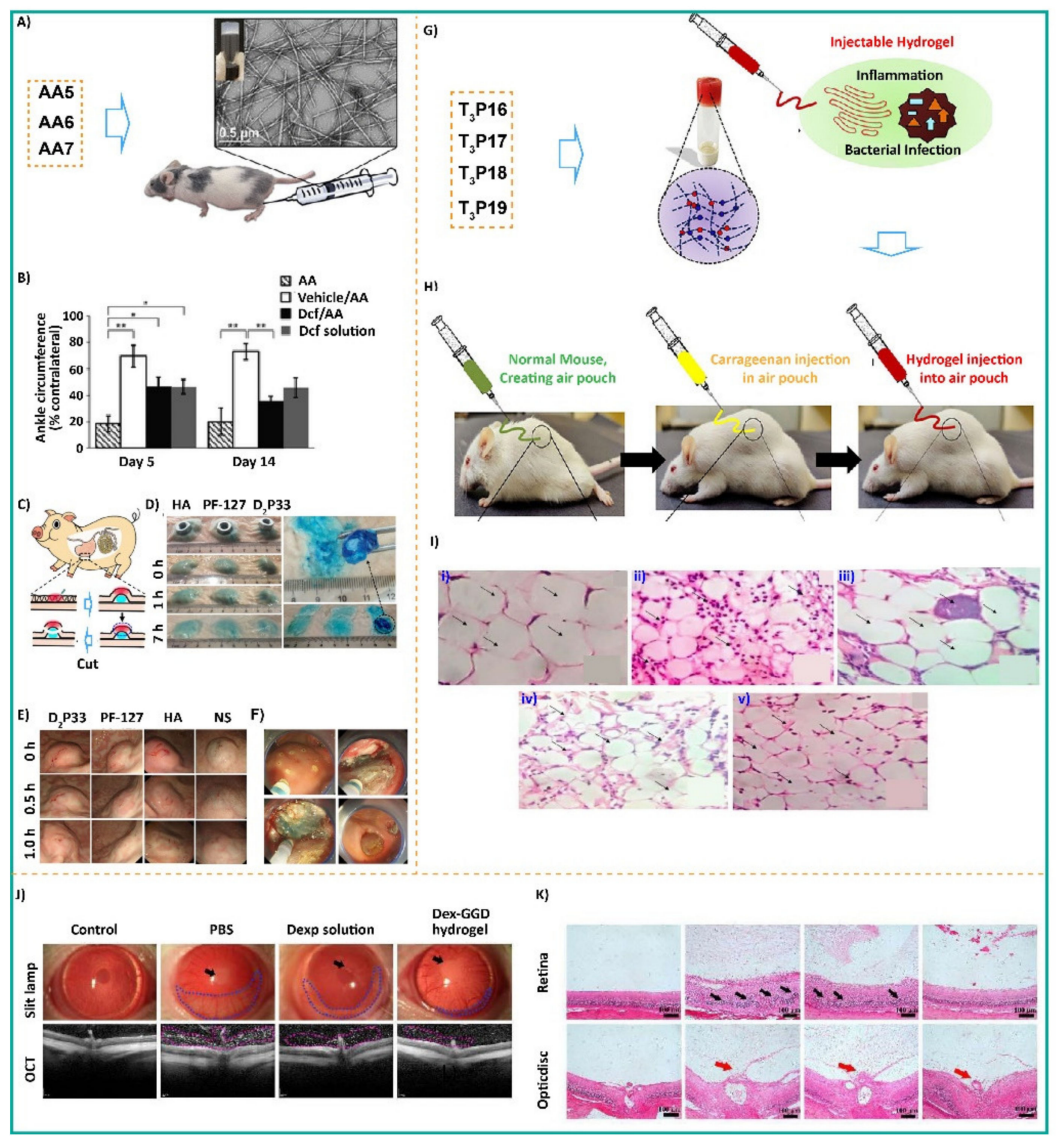
(**A**) Schematic illustration for injectable amino acid gel for in vivo experiment. (**B**) Ankle circumference graph of all four groups of mice with marked statistical differences; reproduced with permission from Ref. [53]. Copyright (2019) American Chemical Society. (**C**) ESD filler graphic of the D_2_P33 hydrogel. (**D**) Photographs of mucosal elevation of resected stomach altered over time at 37 °C. (**E**) Photographs of endoscopic elevation of injected hydrogel in a living mini-pig’s stomach. (**F**) Post-injectable ESD procedure of D_2_P32 hydrogel; reproduced with permission from Ref. [84]. Copyright (2020) American Chemical Society. (**G**,**H**) Schematic illustration of injectable hydrogels for in vitro and in vivo applications. (**I**) Histopathological images of endothelial tissue: (i) normal rat; (ii) carrageenan treated; (iii) dichlorofenac sodium treated; (iv,v) after treating with the nanogels at lower (0.25%) and higher concentrations (0.5%), respectively.; reproduced with permission from Ref. [118]. Copyright (2021) American Chemical Society. (**J**) Slit-lamp and OCT images of EAU rats treated by intravitreal injection of PBS, Dexp solution, and Dex-GGD hydrogel at the 12th day after immunization (blue circle indicates hypopyon, and magenta circle indicates inflammatory cells infiltration). (**K**) Histological observation of the retina and optic disc from EAU rats treated by intravitreal injection of PBS, Dexp solution, and Dex-GGD hydrogel at the 15th day post-immunization (the black arrow indicates retinal folds, and the red arrow indicates inflammatory exudate); reproduced with permission from Ref. [119]. Copyright (2021) Elsevier.

Nilsson et al. employed a diclofenac-loaded thixotropic and injectable hydrogel for in vivo drug delivery [53] (Figure 21A). Diclofenac is a well-known nonsteroidal anti-inflammatory drug used to efficiently relieve pain in vivo in a mouse model. To reach the target, the diclofenac encapsulated hydrogel was administrated through the injection into the afflicted ankle joint of numerous animal groups with the help of anesthesia (1% isoflurane). The drug-loaded hydrogel exhibited sustained drug release for nearly 2 weeks post-injection for effective pain mitigation (Figure 21B). Endoscopic submucosal dissection (ESD), a minimally invasive operation, showed its potential to remove polyps and early-stage tumors. However, cushions are elevated by traditional fluids. Therefore, in situ hydrogel formation is an effective approach to solving the problem and in vivo applications. In connection with this, Bai et al. injected the peptide hydrogel in fresh resected and living mini-pig’s stomachs along with Pluronic F-127 (PF-127, commercial thermosensitive polymer) and sodium hyaluronate (HA, a clinical ESD filler NS) to check the submucosal injection performance [84]. The data suggested that Fmoc-TyrLeu (D_2_P32) can be an ideal filler, as shown in Figure 21D,E. The fractional inflammatory cell infiltration was noted in muscular tissues adjacent to injection sites of PF-127 and HA, whereas D_2_P32 showed nominal pathological changes. It was observed that filling the D_2_P32 hydrogel exhibited higher mucosal elevation compared to the PF-127 hydrogel and HA after instant injection and was more intense at the injection site. In addition, D_2_P32 showed good ESD filler when the gel was injected through an endoscopic entry needle to make submucosal cushions in porcine stomachs, as can be seen from Figure 21.

Despite progress in peptide-based biomaterials in biomedicine, the supramolecular injectable peptide hydrogel for acute inflammation is still challenging [118]. Therefore, to shed light on the therapeutic efficacy of the injectable hydrogels, Konar et al. showed the in vitro and in vivo anti-inflammatory and anti-bacterial properties of the hydrogels (T_3_P16–T_3_P19) (Figure 21G) [118]. To check the effectiveness of the hydrogel, a set of five groups of animals was prepared in which normal mice belonged to Group 1. In contrast, Group 2 and Group 3 were divided into air pouch-developed mice and standard drug dichlorofenac sodium-treated diseased mice, respectively. Groups 4 and 5 were divided based on hydrogel dose, diseased mice with a low and high dose of hydrogel. It was observed that the number of inflammatory cells decreased to a greater extent in hydrogel-injected mice (Group 4 and 5) in comparison to diseased mice (Figure 21H,I). As a result, the tissue regained its normal architecture. The anti-inflammatory response was dose dependent, and therefore, the higher dose showed better efficacy. In addition, the hydrogel is a potent and safe anti-inflammatory agent as revealed from WBC counts and lipid peroxidation (LPO) assay. Similarly, Li et al. employed the hydrogel for the anti-inflammatory response in the LPS-activated RAW 264.7 macrophages in vitro, followed by in vivo application in an experimental autoimmune uveitis (EAU) rat model [119]. The hydrogel displayed a significant alleviated response with less accumulation of pus. In addition, the gel showed a superior inhibitor effect along with inflammatory infiltration in the posterior segments (Figure 21J,K). Since the pioneer demonstration by Jenner about vaccination in the eighteenth century, scientists worldwide have been trying to develop vaccines to treat chronic infections and cancers. To be effective toward chronic infections and cancers, a strong CD8+ T-cell response is always desirable for the vaccines; therefore, there is an increasing interest to develop a vaccine adjuvant with the available licensed vaccine adjuvants, such as aluminum salts, and oil-in-water emulsion MF59 properties. Therefore, Yang et al. applied the resulting thixotropic hydrogel as a vaccine adjuvant [122]. For that, an antigen, ovalbumin (OVA), was mixed during the gel preparation, and the formed gel showed injectable properties owing to its non-covalent interactions. Similarly, the enantiomeric gel was prepared, as D-peptides are well known for their better stability in biological media. Subcutaneously injected D-gel exhibited better anti-OVA IgG titers than L-gel and the other control. It was observed that its vaccine adjuvant potency is highly dependent on its peptide sequence and the appropriate aromatic capping unit. In a similar framework, Huang et al. showed the enhanced (~2.1 fold) antigen uptake by antigen encapsulated hydrogel in comparison to free OVA on bone marrow dendritic cells (BMDCs) through the endocytic pathway [124]. This can be explained in terms of strong interactions between the gel and protein to form the hybrid nanostructure. The gel showed potent anti-OVA IgG production when the gel was subcutaneously injected into C57 mice. In addition, the group utilized hydrogel splenocyte proliferation.

**Figure 22:**
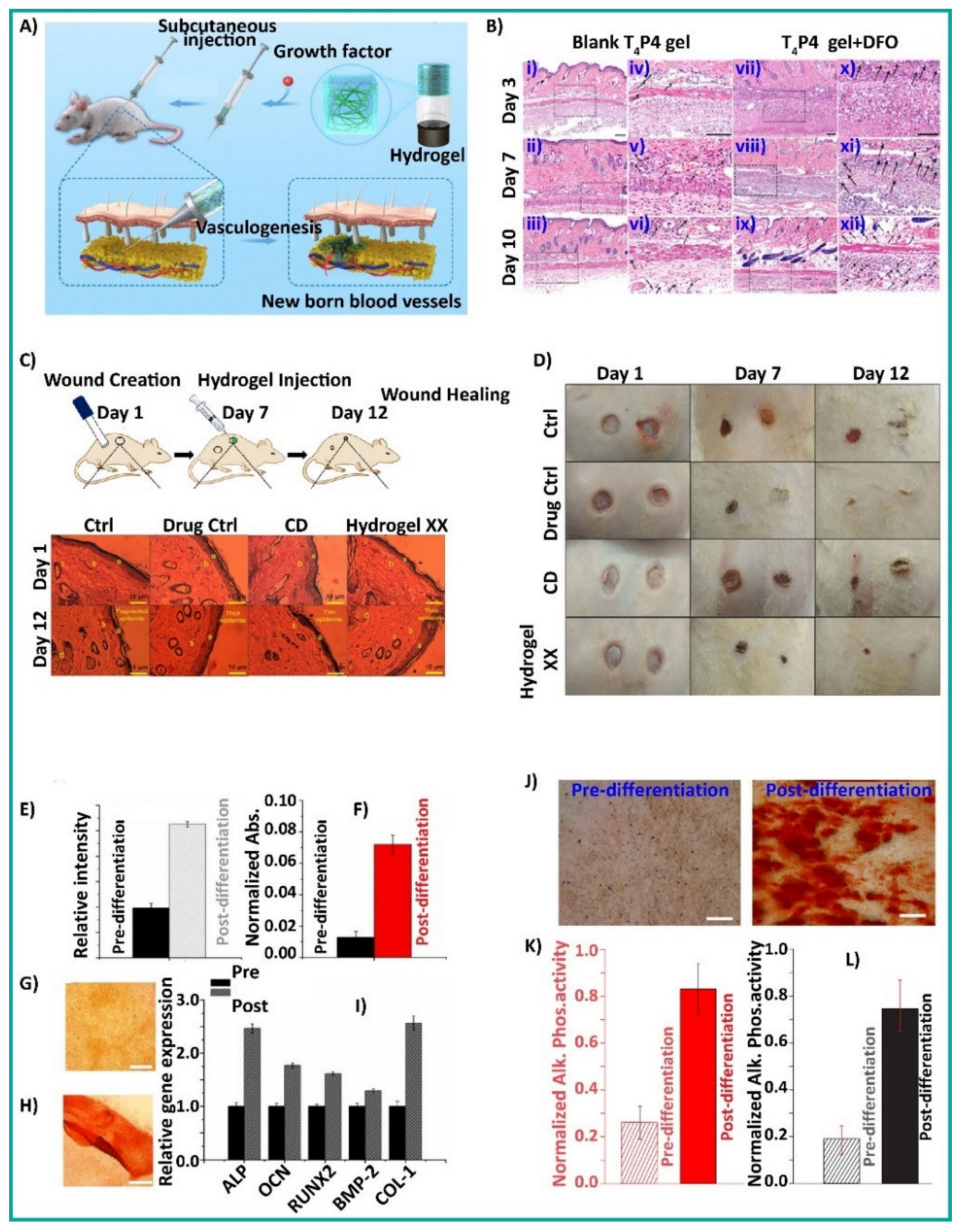
(**A**) Schematic illustration of in vivo angiogenesis employing a tetrapeptide supramolecular hydrogel; (**B**) (i), (ii), and (iii) H&E stained images of the DFO excluded implanted T_4_P4 gel at 3, 7, and 10 days, respectively; (iv), (v), and (vi) high amplification images of the boxed areas shown in (i), (ii), and (iii), respectively; (vii), (viii), and (ix) H&E stained images of the DFO included implanted T_4_P gel at 3, 7, and 10 days, respectively; (x), (xi), and (xii) high amplification images of the boxed areas shown in (vii), (viii), and (ix), respectively; reproduced with permission from Ref. [123]. Copyright (2018) American Chemical Society. (**C**) H&E stained epithelial tissue histology images; a, epidermis layer; b, dermis layer; c, sweat gland; and d, sebaceous gland; (**D**) Digital images of D_2_P12 abd β-CD co-assembled hydrogel that assisted in vivo wound healing at day 1, 7, and 12, respectively; reproduced with permission from Ref. [151]. Copyright (2020) American Chemical Society. Osteogenenic response on the Alginate/D_2_P12 composite hydrogel. (**E**) Alkaline phosphatase (ALP) activity. (**F**) Calcification quantification of Alizarin red stained MC3T3-E1 preosteoblast cells 3 days after seeding on alginate/D_2_P14 composite hydrogel and following 14 days of osteogenic differentiation; optical images of Alizarin red stained MC3T3-E1 preosteoblast cells; (**G**) after 3 days; (**H**) after 14 days. (**I**) Quantification of relative mRNA expression of osteogenic genes (ALP, OCN, RUNX2, BMP-2, and Col-I) of MC3T3-E1 preosteoblast cells 3 days after seeding on alginate/D_2_P14 composite hydrogel and following 14 days of osteogenic differentiation; reproduced with permission from Ref. [175]. Copyright (2019) MDPI AG; MDPI. Osteogenic response of MC3 T3-E1 cells on the HA/D_2_P27 composite hydrogel. (**J**) Optical images of Alizarin red stained MC3 T3-E1 preosteoblast cells prior to (pre-differentiation) and after (post-differentiation) 14 days of osteogenic differentiation on HA/D_2_P27 composite hydrogel; reproduced with permission from Ref. [167]. Copyright (2021) MDPI AG; MDPI. (**K**) Calcification. (**L**) Alkaline phosphatase (ALP) activity quantification of Alizarin red stained of MC3 T3-E1 preosteoblasts before and after 14 days of osteogenic differentiation on HA/D_2_P27 composite hydrogel; reproduced with permission from Ref. [167]. Copyright (2021) MDPI AG; MDPI.

To replace or regenerate the damaged or diseased tissue, tissue engineering showed its ability in the field of biotechnology and nanomedicine [25]. In light of this engineering process, in vivo, the vascularization process comprises a cascade of actions regulated by the complex signals from both ECM and growth factors and the spatial and temporal architectures. Hence, Li et al. implanted deferoxamine (DFO)-loaded T_4_P4 gel through subcutaneous injection into the dorsal side of mice because of their nice shear-shining and recovery properties such as pure gel [123]. DFO is a well-known organic drug used to provoke vascularization. The presence of the DFO-loaded hydrogel showed generation of new red blood cells within the blood vessels. The implantation helped the surrounding connective tissues enhance the blood vessels compared to pure gel at day 3, which further increased over time, as can be seen from H&E staining data (Figure 22A,B). This can be attributed to the bioavailability and biostability of DFO sustainably released from T_4_P4 gel in vivo. Chen et al. employed the hydrogel for the biological application in the field of atherosclerosis, and for that, the drug T0901317 was encapsulated into the hydrogel [182]. Atherosclerosis is one kind of cardiovascular disease triggered by chronic lipid metabolic disorders, which is ultimately the root of morbidity and mortality worldwide. The encapsulated hydrogel selectively attacks only the macrophages of proatherogenic mice, causing activation of cholesterol metabolism without no lipogenic adverse effect, confirmed from in vitro and in vivo studies. As a biological application, Das et al. performed the cutaneous wound healing assay to check the in vivo wound healing efficiency [151]. To do so, in vivo wounds were formed in Wistar albino male rats, and as expected, the hydrogel displayed satisfactory results as compared to controls (Figure 22C,D). This can be explained in terms of nonfibrillar architectures (as a sealant) and inherent antibacterial efficacy.

Bone tissue engineering is a promising process in which restoration of damaged bone architecture and function is maintained with the help of a biomimetic scaffold. Injectable peptide hydrogels showed their potential owing to their minimally invasive nature. Therefore, Li and co-workers demonstrated the biomedical application of the hydrogel in vitro as a scaffold to promote bone marrow MSC adhesion, cell proliferation, and osteogenic differentiation. Furthermore, the gel showed in vivo applications in a rat femoral defect model in which a healing process was observed near bone tissues. Adler-Abramovich applied the composite hydrogel as a platform for bone tissue regeneration (Figure 22E–I) [175]. In this regard, Das and co-workers also employed a composite hydrogel for osteogenesis (Figure 22J–L) [167].

In this section, biomedical applications were discussed employing thixotropic hydrogels based on amino acid, di-, tri-, tetra-peptide, co-assembled, and finally composite ones in various fields from 3D cell culture to tissue engineering.

## 10. Challenges and Future Directions

Although short peptide-based thixotropic hydrogels have shown great promise in the field of biomedicine, several limitations remain. The precise mechanism underlying such a unique behavior is yet unclear, and it is critical to consider whether thixotropic activity in peptide hydrogels is caused solely by H-bonding and π-π stacking or by something else. If this is the case, why are no hydrogels naturally thixotropic? Furthermore, many thixotropic hydrogelators contain aromatic capping moieties, which can reduce solubility and promote cytotoxicity in biological media, limiting their practical applicability. In some cases, additives/external factors such as temperature, light, salt, solvent, and so on are required to generate thixotropic hydrogels. Apart from these issues, it has been found that in many cases, the thixotropic behavior cannot be replicated in more than one cycle. In some cases, CDs are utilized to make supramolecular thixotropic hydrogels via host–guest chemistry. To do this, AAP is inserted into the peptide sequence, and the hydrogel is irradiated with light to produce the thixotropic response, limiting its biological applications, as UV-light-triggered *E*→*Z* isomerization is hazardous and can be vastly distributed in biological tissue or in nanomaterials. As a result, there is plenty of room to investigate host–guest-induced thixotropic supramolecular gels using various host compounds, particularly cucurbituril (CB). Throughout the review, except for the co-assembled hydrogel described by Das et al., which can a demonstrate thixotropic reaction at elevated temperatures, it is difficult to discover thixotropic hydrogelators. As a result, it is preferable to create a short-peptide-based thixotropic hydrogel that addresses all of the difficulties stated. Covalent interactions between peptide building blocks, such as disulfide bond formation and imine bond formation, can be investigated to produce durable thixotropic hydrogels. Only a few have been created and researched. Despite the advances and potential uses of these thixotropic hydrogels, there is still much to learn about the precise control of thixotropic gel formation.

## 11. Conclusions

Supramolecular thixotropic peptide hydrogels have received a growing interest in the field of nanomedicine owing to their various biological applications in, e.g., 3D cell culture, anti-inflammatory, anti-bacterial, drug delivery, tissue engineering, regenerative medicine, to name a few, and therefore, it is essential to identify the short-peptide-based hydrogels with thixotropic behavior. In this review, recently reported (within 5 years) low molecular weight-based thixotropic hydrogels were compiled comprehensively, followed by self-assembly-induced thixotropy and their biological application. The thixotropy property of the hydrogels was characterized with the help of a rheometer in which continuous high- and low-magnitude strain was applied alternatively to disrupt the 3D gel network followed by reconstruction at a time frame. The weak noncovalent interaction within the gel network is solely responsible for obtaining the thixotropy. In addition, injectable behavior was also demonstrated by injecting the gel through a syringe in which the quasi-liquid transforms into a gel after coming out through the needle, confirming the self-healing nature. This review will provide ample opportunity not only to the peptide chemist but also to all scientists related to biology to design and execute short peptide-based thixotropic injectable hydrogels for future regenerative medicine.

## Figures and Tables

**Table 1:** List of amino acid (AA)-based thixotropic hydrogelators and respective gelation parameters.

SL No.	Peptide Sequence	MGC (wt%)	Gelation Media	T_gel_ (°C)	Moduli (Before Mechanical Stress)		Moduli (After Mechanical Stress)		Moduli (Stress-Free Conditions)		Morphology	Application
					G′	G″	G′	G″	G′	G″		
1.	Fmoc-Lys-Bct (AA1) [54]	0.30	Water (pH 7.4)	60	10^3^–10^4^	10^2^–10^3^	~10^1^	10^1^–10^2^	10^3^–10^4^	10^2^–10^3^	Fibrous networks	Cell proliferation and bacterial activity
2.	Fmoc-Tyr-3NO_2_ (AA2) [61]	0.25	Water (pH 7.0)	NR	~10^3^	~10^2^	~10^0^	~10^1^	~10^3^	~10^2^	Fibrillar networks	Antimicrobial activity
3	Myr-Phe-COOH (AA3, L-form) [62]	0.25	PB (pH 7.0)	37	~10^2^	~10^2^	10^0^–10^1^	10^0^–10^1^	10^1^–10^2^	10^1^–10^2^	Long fibers	Enzyme entrapment
4.	Myr-Phe-COOH (AA3, D-form) [62]	0.25	PB (pH 7.0)	37	10^2^–10^3^	10^2^–10^3^	10^0^–10^1^	~10^1^	10^1^–10^2^	10^1^–10^2^	Long fibers	Enzyme entrapment
5.	Fmoc-Phe-DAP (AA5) [53]	1.42	Water	NR	~10^3^	~10^2^	10^0^–10^1^	~10^1^	~10^3^	~10^2^	Flat, tape-like structures	In Vivo Drug delivery
6.	Fmoc-3F-Phe-DAP (AA6) [53]	~1.48	Water	NR	~10^4^	10^3^–10^4^	10^1^–10^2^	10^2^–10^3^	~10^4^	10^3^–10^4^	Fibrils/tapes	In Vivo Drug delivery
7.	Fmoc-F^5^-Phe-DAP (AA7) [53]	1.71	Water	NR	10^3^–10^4^	10^2^–10^3^	10^1^–10^2^	10^2^–10^3^	10^3^–10^4^	10^2^–10^3^	Twisted fibers and tapes	In Vivo Drug delivery
8.	Fmoc-Phe-COOH (AA8) [68]	0.19	Water: DMSO (98:2, *v*/*v*)	NR	NR	NR	NR	NR	NR	NR	NR	NR
		0.19	Water (NaOH-GdL)	NR	NR	NR	NR	NR	NR	NR	NR	NR
9.	Fmoc-3F-Phe-COOH (AA9) [68]	0.20	Water: DMSO (98:2, *v*/*v*)	NR	10^3^–10^5^	10^3^–10^4^	~10^2^	10^2^–10^3^	10^3^–10^5^	10^3^–10^4^	Fibrillar networks	NR
		0.20	Water (NaOH-GdL)	NR	10^2^–10^3^	10^1^–10^2^	~10^1^	10^1^–10^2^	10^2^–10^3^	10^1^–10^2^	Fibrillar networks	NR
10.	Fmoc-F^5^-Phe-COOH (AA10) [68]	~0.24	Water: DMSO (98:2, *v*/*v*)	NR	10^3^–10^4^	~10^3^	10^0^–10^1^	~10^2^	~10^3^	~10^2^	Fibrillar networks	NR
		~0.24	Water (NaOH-GdL)	NR	10^2^–10^3^	10^1^–10^2^	~10^1^	10^1^–10^2^	10^2^–10^3^	10^1^–10^2^	Fibrillar networks	NR
11.	Fmoc-Lys (Fmoc)-COOH (AA11) [69]	0.30	PB (pH 6.0)	NR	10^3^–10^4^	~10^2^	~10^1^	10^1^–10^2^	~10^3^	~10^2^	Fibrous networks	NR
		0.30	PB (pH 7.4)	NR	~10^3^	10^1^–10^2^	~10^0^	~10^0^	10^1^–10^2^	10^0^–10^1^	Fibrous networks	NR

MGC: minimum gelation concentration; wt%: mg/100 μL; T_gel_: gel transition temperature; G′: storage modulus; G″: loss modulus; Fmoc: fluorenyl-9-methoxycarbonyl; Lys: lysine; Bct: biocytine; Tyr: tyrosine; Myr: myristic acid; Phe: phenylalanine; 3F-Phe: 3-fluoro-phenylalanine; F^5^-Phe: 2,3,4,5,6-Pentafluoro-phenylalanine; PB: phosphate buffer; DAP: diaminopropane; DMSO: dimethyl sulfoxide; NaOH: sodium hydroxide; GdL: glucono-δ-lactone; NR: not reported.

**Table 2:** List of dipeptide (D_2_P)-based thixotropic hydrogelators and respective gelation parameters.

SL No.	Peptide Sequence	MGC (wt%)	Gelation Media	T_gel_ (°C)	Moduli (Before Mechanical Stress)		Moduli (After Mechanical Stress)		Moduli (Stress-Free Conditions)		Morphology	Application
					G′	G″	G′	G″	G′	G″		
1.	H_2_N-Gly-Phe-C_12_ (D_2_P1) [72]	0.83	Water (pH 6.6)	48	NA	NA	NA	NA	NA	NA	Intertwined nanofibrillar networks	Antimicrobial activity
2.	H_2_N-β-Ala-Phe-C_12_ (D_2_P2) [72]	1.12	Water (pH 6.6)	52	~10^2^	~10^1^	~10^−1^	10^−1^–10^0^	10^0^–10^2^	10^0^–10^1^	Intertwined nanofibrillar networks	Antimicrobial activity
3.	H_2_N-GABA-Phe-C_12_ (D_2_P3) [72]	0.99	Water (pH 6.6)	58	~10^2^	~10^1^	~10^−1^	10^−1^–10^0^	10^0^–10^2^	10^0^–10^1^	Intertwined nanofibrillar networks	Antimicrobial activity
4.	H_2_N-AVAB-Phe-C_12_ (D_2_P4) [72]	0.90	Water (pH 6.6)	64	~10^2^	~10^1^	~10^−1^	10^−1^–10^0^	10^0^–10^2^	10^0^–10^1^	Intertwined nanofibrillar networks	Antimicrobial activity
5.	H_2_N-EACA-Phe-C_12_ (D_2_P5) [72]	0.77	Water (pH 6.6)	68	10^2^–10^3^	10^1^–10^2^	~10^−1^	10^−1^–10^0^	10^0^–10^3^	10^0^–10^2^	Intertwined nanofibrillar networks	Antimicrobial activity
6.	Boc-Phe-Aib (D_2_P6) [73]	0.4	Water	67.8	~3 × 10^5^	~0.0–0.5 × 10^5^	~0.0 × 10^5^	~0.5 × 10^5^	~2.5–3 × 10^5^	~0.5 × 10^5^	Rod-like fibrillar network	HCl sensing
7.	Nap-Phe-Phe-COOH (D_2_P7) [74]	0.1	Water/Ca^2+^ (pH 3.9)	NR	~10^5^	~10^4^	~10^2^	~10^2^	10^4^–10^5^	10^3^–10^4^	Mat of fibrous networks	NR
		0.1	Water/GdL (pH 3.9)	NR	10^4^–10^5^	10^3^–10^4^	~10^2^	~10^2^	10^4^–10^5^	10^3^–10^4^	Mat of fibrous networks	NR
		0.1	Water/DMSO (pH 3.9)	NR	10^4^–10^5^	10^3^–10^4^	~10^1^	~10^2^	~10^3^	~10^2^	Crystalline morphology	NR
8.	Cbz-Phe-Phe-COOH (D_2_P9) [75]	0.5	Water: MeOH (3:1, *v*/*v*)	NR	10^2^–10^3^	10^1^–10^2^	10^0^–10^1^	~10^1^	~10^3^	10^1^–10^2^	Nanofibers	NR
	Cbz-Phe-Phe-COOH (D_2_P9) [76]	~1.78	Water (pH 7.4)	NR	~10^2^	10^1^–10^2^	~10^0^	~10^0^	10^1^–10^2^	10^0^–10^1^	Fibrous networks	NR
	Cbz-Phe-Phe-COOH (D_2_P9) [77]	1.3	PB (pH ~8) Heat-Cool	50	~10^4^	~10^3^	~10^2^	10^2^–10^3^	10^3^–10^4^	~10^3^	Long entangled nanofibrous networks	Cellular adhesion
	Cbz-Phe-Phe-COOH (D_2_P9) [77]	1.3	PB (pH ~8) Sonication	40	~10^2^	NR	NR	NR	NR	NR	Short twisted ribbons	Cellular adhesion
9.	Ste-^D^Val-^D^Ala-COONa (D_2_P11) [78]	0.02	Water	NR	~4–5 × 10^2^	~2 × 10^2^	~10^0^	~10^1^	~5 × 10^2^	~2 × 10^2^	Left-handed twist nanoribbons	NR
10.	Amoc-Phe-Leu-COOH (D_2_P12) [79]	20 mM	Water (pH 7.4)	NR	10^4^–10^5^	~10^3^	~10^2^	10^2^–10^3^	~10^4^	~10^3^	Nanofibrillar networks	Cell proliferation and antibacterial efficacy
11.	Amoc-Phe-Tyr-COOH (D_2_P13) [79]	20 mM	Water (pH 7.4)	NR	10^3^–10^4^	10^2^–10^3^	10^1^–10^2^	10^1^–10^2^	10^3^–10^4^	10^2^–10^3^	Nanofibrillar networks	Cell proliferation and antibacterial activity
12.	Fmoc-Phe-Phe-COOH (D_2_P14) [76]	~0.13	Water (pH 7.4)	NR	10^3^–10^4^	~10^3^	10^1^–10^2^	~10^2^	10^3^–10^4^	~10^3^	Fibrous networks	NR
13.	Nap-Phe-Phe-COOH (D_2_P15) [76]	~0.25	Water (pH 7.4)	NR	~10^3^	10^2^–10^3^	~10^2^	~10^2^	~10^3^	10^2^–10^3^	Fibrous networks	NR
14.	Fmoc-Phe-Tyr-COOH (D_2_P16) [76]	~0.54	Water (pH 7.4)	NR	~10^3^	~10^2^	NR	NR	NR	NR	Nanotubes	NR
15.	Nap-Phe-Tyr-COOH (D_2_P17) [76]	~1.02	Water (pH 7.4)	NR	~10^3^	~10^2^	NR	NR	NR	NR	Fibrous networks	NR
16.	Cbz-Phe-Tyr-COOH (D_2_P18)	NA	Water (pH 7.4)	NA	NA	NA	NA	NA	NA	NA	Spherical aggregates	NR
17.	Fmoc-Phe-Leu-COOH (D_2_P19) [76]	~0.50	Water (pH 7.4)	NR	~10^3^	~10^2^	NR	NR	NR	NR	Fibrous networks	NR
18.	Nap-Phe-Leu-COOH (D_2_P20) [76]	~0.92	Water (pH 7.4)	NR	10^1^–10^2^	10^1^–10^2^	NR	NR	NR	NR	Nanoribbons	NR
19.	Cbz-Phe-Leu-OH (D_2_P21) [76]	NA	Water (pH 7.4)	NA	NA	NA	NA	NA	NA	NA	Spherical aggregates	NR
20.	Fmoc-Phe-Ser-COOH (D_2_P22) [76]	~0.71	Water (pH 7.4)	NR	10^2^–10^3^	~10^2^	NR	NR	NR	NR	Interwoven fibrous network	NR
21.	Nap-Phe-Ser-COOH (D_2_P23) [76]	~1.74	Water (pH 7.4)	NR	10^1^–10^2^	~10^1^	NR	NR	NR	NR	Fibrous networks	NR
22.	Cbz-Phe-Ser-COOH (D_2_P24) [76]	NA	Water (pH 7.4)	NA	NA	NA	NA	NA	NA	NA	Spherical aggregates	NR
23.	Myr-Phe-Phe-COOH (D_2_P25) [80]	0.05	Water (pH 7.4)	48 ± 4	~10^4^	~10^3^	~10^−1^	10^0^–10^1^	10^3^–10^4^	10^2^–10^3^	Nanofiber networks	Biocompatibility
24.	Myr-Trp-Phe-COOH (D_2_P26) [81]	0.25	Water (pH 7.46)	NR	~10^3^	10^2^–10^3^	~10^0^	~10^1^	~10^3^	10^2^–10^3^	Cross-linked nanofiber network	NR
25.	Py-Lys-Cys-CONH_2_(D_2_P27) [40]	1.0	Tris buffer (pH 8)	75 ± 0.5	~10^2^	10^1^–10^2^	NR	NR	NR	NR	Densely packed thin nanofibrous network	Enzyme entrapment
26.	Azo-Lys-Cys-CONH_2_(D_2_P28) [82]	1.15	Water (pH 10)	57	10^3^–10^4^	10^2^–10^3^	~10^2^	~10^1^	10^3^–10^4^	10^2^–10^3^	Thin nanofibrous network	Dye removal
27.	Fmoc-Lys(Fmoc)-Asp-COOH (D_2_P29) [83]	0.002	Water	NR	10^2^–10^3^	~10^2^	~10^−1^	~10^0^	10^2^–10^3^	~10^2^	Entangled fibrous networks	NR
28.	Fmoc-Phe-Leu-COOH (D_2_P30) [84]	1.001	PBS (pH ~8)	NR	~10^4^	~10^3^	NR	NR	NR	NR	A mixture of nanofibers and straight rods	Endoscopic submucosal dissection filler
29.	Fmoc-Tyr-Leu-COOH (D_2_P31) [84]	1.033	PBS (pH ~8)	NR	~10^4^	~10^3^	NR	NR	NR	NR	Long nano fibrous networks	
30.	Fmoc-Leu-Leu-COOH (D_2_P32) [84]	0.933	PBS (pH ~8)	NR	~10^3^	10^2^–10^3^	NR	NR	NR	NR	Long nano fibrous networks	
31.	Fmoc-Tyr-Leu-COOH (D_2_P33) [84]	0.949	PBS (pH ~8)	NR	~10^3^	~10^2^	NR	NR	NR	NR	Long nano fibrous networks	
32.	Naproxen-Phe-Phe-COOH (D_2_P34) [85]	0.5	Water: DMSO (98:2, *v*/*v*)	202	~10^4^	~10^3^	NR	NR	NR	NR	Nanofibrous networks	NR
33.	Py-Val-Val-COOMe(D_2_P35) [86]	1.0	PB: DMSO (90:10, *v*/*v*), pH 8	NR	~10^3^	10^2^–10^3^	10^0^–10^1^	10^1^–10^2^	~10^3^	10^2^–10^3^	Entangled nanofibrous networks	NR
34.	Py-Leu-Leu-COOMe(D_2_P36) [86]	0.528	PB: DMSO (90:10, *v*/*v*), pH 8	NR	~10^2^	10^1^–10^2^	NR	NR	NR	NR	Entangled nanofibrous networks	NR
35.	Py-Phe-Phe-COOMe(D_2_P37) [86]	0.298	PB: DMSO (90:10, *v*/*v*) pH 8	NR	~10^3^	~10^2^	10^0^–10^1^	10^1^–10^2^	~10^3^	~10^2^	Entangled nanofibrous networks	NR
36.	Fmoc-Phe-Val-COOH (D_2_P38) [87]	0.972	Water (pH 7.4)	NR	10^4^–10^6^	10^4^–10^6^	NR	NR	NR	NR	Fibrous networks	3D cell culture

MGC: minimum gelation concentration; wt%: mg/100 μL; T_gel_: gel transition temperature; G′: storage modulus; G″: loss modulus; C_12_: dodecyl amine; β-Ala: β-alanine; GABA: gamma-aminobutyric acid; AVAB: 5-aminovaleric acid betaine; EACA: 6-aminocaproic acid; Boc: tert-butyloxycarbonyl; Aib: 2-aminoisobutyric acid; Phe: phenylalanine; Fmoc: fluorenyl-9-methoxycarbonyl; Nap: naphthoic acid; Cbz: benzyloxycarbonyl; Ste: stearic acid; Val: valine; Ala: alanine; Amoc: 9-anthracenemethoxycarbonyl; Leu: leucine; Tyr: tyrosine; Ser: serine; Lys: lysine; Trp: tryptophan; Asp: aspartic acid; Myr: myristic acid, Py: pyrene butyric acid; Azo: azobenzene acid; PB: phosphate buffer; Tris: tris(hydroxymethyl)aminomethane; PBS: phosphate buffer saline; DMSO: dimethyl sulfoxide; MeOH: methanol; NaOH: sodium hydroxide; GdL: glucono-δ-lactone; NR: not reported.

**Table 3:** List of cyclic dipeptide (CDP)-based thixotropic hydrogelators and respective gelation parameters.

SL No.	Peptide Sequence	MGC (wt%)	Gelation Media	T_gel_ (°C)	Moduli (Before Mechanical Stress)		Moduli (After Mechanical Stress)		Moduli (Stress-Free Conditions)		Morphology	Application
					G′	G″	G′	G″	G′	G″		
1.	Cyclo[L-Glu(OFmoc)-L-Glu] (CDP-1) [105]	0.4	PBS: DMSO (5:1, *v*/*v*)	161	~10^3^	~10^2^	NR	NR	~10^3^	~10^2^	Tangled fibrous networks	NR
2.	Cyclo[D-Glu(OFmoc)-D-Glu] (CDP-2) [105]	0.4	PBS: DMSO (5:1, *v*/*v*)	160	NR	NR	NR	NR	NR	NR	Tangled fibrous networks	NR
3.	Cyclo-(Leu-Phe) (CDP-3) [108]	0.2	Water: DMSO (9:1, *v*/*v*)	NR	10^4^–10^5^	~10^3^	~10^1^	10^1^–10^2^	10^4^–10^5^	~10^3^	Fibrous networks	NR
4.	Cyclo-(Trp-Tyr) (CDP-4) [106]	0.5	Water	NR	~10^4^	~10^4^	~10^1^	~10^2^	~10^4^	~10^4^	Fibrous networks	NR

Minimum gelation concentration; wt%: mg/100 μL; T_gel_: gel transition temperature; G′: storage modulus; G″: loss modulus; Fmoc: fluorenyl-9-methoxycarbonyl; Glu: glutamic acid; Leu: leucine; Phe: phenylalanine; Trp: tryptophan; Tyr: tyrosine; PBS: phosphate buffer saline; DMSO: dimethyl sulfoxide; NR: not reported.

**Table 4:** List of tripeptide (T_3_P)-based thixotropic hydrogelators and respective gelation parameters.

SL No.	Peptide Sequence	MGC (wt%)	Gelation Media	T_gel_ (°C)	Moduli (Before Mechanical Stress)		Moduli (After Mechanical Stress)		Moduli (Stress-Free Conditions)		Morphology	Application
					G′	G″	G′	G″	G′	G″		
1.	Fmoc-Gly-Phe-Phe-COOH (T_3_P1) [111]	0.1	DMEM (pH 7.4)	NR	10^3^–10^4^	~10^2^	~10^0^	~10^0^	10^2^–10^3^	10^1^–10^2^	Nanofibrous networks	3D Cell Culture of Tumor Spheroids
2.	PTZ-Gly-Phe-Phe-COOH (T_3_P2) [111]	0.05	DMEM (pH 7.4)	NR	10^3^–10^4^	10^2^–10^3^	~10^1^	~10^1^	~10^3^	10^2^–10^3^	Nanofibrous networks	3D Cell Culture of Tumor Spheroids
3.	Py-PhePhe-Lys-CONH_2_ (T_3_P3) [57]	1.8	1:1 Water:ACN	51	~10^3^	~10^2^	10^0^–10^1^	10^1^–10^2^	~10^3^	~10^2^	Tightly knitted fibrous networks	PA sensing
4.	NH_2_-His-Ile-AUDA-COOMe (T_3_P4) [113]	0.4	PBS (pH 7.46)	44	~10^4^	~10^3^	~10^1^	~10^2^	~10^4^	~10^3^	Nanospheres	3D Cell Culture of Fibroblasts
5.	Fmoc-Phe-Phe-Cys-COOH (T_3_P5) [114]	2.8	Water (pH 11)	NR	10^4^–10^5^	10^3^–10^4^	~10^2^	~10^3^	10^4^–10^5^	10^3^–10^4^	Coiled and interlaced fibrous networks	Printable Hydrogels
6.	Fmoc-His-^D^Phe-^D^Phe-CONH_2_ (T_3_P6) [115]	1.0	Water:DMSO (35:65, *v*/*v*)	NR	~10^3^	10^2^–10^3^	~10^2^	~10^2^	~10^3^	~10^2^	Thin and long wavy threads/fibers	Bactericidal Activities
7.	Fmoc-Arg-^D^Phe-^D^Phe-CONH_2_ (T_3_P7) [115]	1.0	Water:DMSO (35:65, *v*/*v*)	NR	~10^4^	~10^3^	10^1^–10^2^	10^1^–10^2^	10^3^–10^4^	10^2^–10^3^	Nanofiber/thread-like structures	Bactericidal Activities
8.	BPAA-β-Ala-Phe-Phe-COOH (T_3_P8) [116]	0.023	PBS (pH 7)	NR	10^3^–10^4^	~10^3^	10^0^–10^1^	~10^1^	10^3^–10^4^	~10^3^	Nanotubes	Cartilage tissue engineering
9.	BPAA-Phe-Phe-β-Ala-COOH (T_3_P9) [116]	0.023	PBS (pH 7)	NR	10^1^–10^2^	10^0^–10^1^	NR	NR	NR	NR	Fibrous networks	Cartilage tissue engineering
10.	BPAA-Phe-Phe-Ala-COOH (T_3_P10) [117]	0.023	Water (pH 7)	NR	~10^4^	~10^3^	10^0^–10^1^	10^1^–10^2^	~10^4^	~10^3^	Short nanoribbons	Tissue engineering
11.	BPAA-Phe-Phe-Gly-COOH (T_3_P11) [117]	0.002	Water (pH 7)	NR	10^2^–10^3^	10^1^–10^2^	~10^1^	10^1^–10^2^	~10^3^	~10^2^	Nanofibrous networks	Tissue engineering
12.	BPAA-Phe-Gly-Phe-COOH (T_3_P12) [117]	NA	NA	NA	NA	NA	NA	NA	NA	NA	NA	NA
13.	BPAA-Gly-Phe-Phe COOH (T_3_P13) [117]	0.002	Water (pH 7)	NR	10^3^–10^4^	10^2^–10^3^	~10^1^	10^1^–10^2^	10^3^–10^4^	10^2^–10^3^	Nanofibrous networks	Tissue engineering
14.	BPAA-Ala-Phe-Phe-COOH (T_3_P14) [117]	0.023	Water (pH 7)	NR	~10^4^	~10^3^	~10^1^	~10^2^	~10^4^	~10^3^	Nanofibrous networks	Tissue engineering
15.	BPAA-Phe-Ala-Phe-COOH (T_3_P15) [117]	NA	NA	NA	NA	NA	NA	NA	NA	NA	NA	NA
16.	Boc-Ava-Phe-Phe-COOH (T_3_P16) [118]	PB pH 7.5	0.07	33	10^3^–10^4^	10^2^–10^3^	~10^1^	~10^2^	10^3^–10^4^	10^2^–10^3^	Fibrillar networks	Anti-Inflammatory and Anti-bacterial activity
17.	Boc-Ava-^L^Phe-^D^Phe-COOH (T_3_P17) [118]	PB pH 7.5	0.15	32	~10^3^	10^2^–10^3^	~10^1^	~10^1^	~10^3^	10^2^–10^3^	Fibrillar networks	Anti-Inflammatory and Anti-bacterial activity
18.	Boc-Ava-^D^Phe-^L^Phe-COOH (T_3_P18) [118]	PB pH 7.5	0.15	35	10^3^–10^4^	~10^3^	10^1^–10^2^	10^0^–10^1^	~10^3^	~10^3^	Fibrillar networks	Anti-Inflammatory and Anti-bacterial activity
19.	Boc-Ava-^D^Phe-^D^Phe-COOH (T_3_P19) [118]	PB pH 7.5	0.08	34	10^4^–10^5^	10^3^–10^4^	~10^2^	~10^3^	10^4^–10^5^	10^3^–10^4^	Fibrillar networks	Anti-Inflammatory and Anti-bacterial activity

MGC: minimum gelation concentration; wt%: mg/100 μL; T_gel_: gel transition temperature; G′: storage modulus; G″: loss modulus; Fmoc: fluorenyl-9-methoxycarbonyl; Boc: tert-butyloxycarbonyl; PTZ: phenothiazine-10-acetic acid; DMEM: Dulbecco’s Modified Eagle Medium; Gly: glycine; Phe: phenylalanine; Lys: lysine; His: histidine; Ile: isoleucine; Arg: arginine; AUDA: 12-(3-Adamantan-1-yl-ureido) dodecanoic acid; Cys: cysteine; Py: pyrene butyric acid; BPAA: 4-biphenylacetic acid; Ava: δ-amino valeric acid; NA: not applicable; NR: not reported.

**Table 5:** List of tetrapeptide (T_4_P)-based thixotropic hydrogelators and respective gelation parameters.

SL No.	Peptide Sequence	MGC (wt%)	Gelation Media	T_gel_ (°C)	Moduli (Before Mechanical Stress)		Moduli (After Mechanical Stress)		Moduli (Stress-Free Conditions)		Morphology	Application
					G′	G″	G′	G″	G′	G″		
1.	Nap-Gly-Phe-Phe-Tyr-COOH (T_4_P1) [122]	NR	PBS (pH 7.4)	NR	10^1^–10^2^	~10^1^	NR	NR	NR	NR	Nanofibrillar structures	Vaccine Adjuvant
2.	Nap-^D^Gly-^D^Phe-^D^Phe-^D^Tyr-COOH (T_4_P2) [122]	NR	PBS (pH 7.4)	NR	10^1^–10^2^	10^0^–10^1^	NR	NR	NR	NR	Nanofibrillar structures	Vaccine Adjuvant
3.	Nap-Phe-Phe-Asp (GlCN)Tyr-COOH (T_4_P4) [123]	0.6	Water (pH 7.4)	NR	10^1^–10^2^	~10^1^	10^0^–10^−1^	~10^0^	10^1^–10^2^	~10^1^	Nanofibrous structures	Vascularization
4.	Nap-His-His-Phe-Phe-COOH (T_4_P5) [124]	0.5	PBS (pH 8.5)	NR	10^1^–10^2^	10^0^–10^−1^	10^−1^–10^−2^	10^−1^–10^0^	10^1^–10^2^	10^0^–10^−1^	Nanofibrous networks	In Vivo Boosts Immune Response
5.	Ac-Phe-Phe-Lys-Cys-CONH_2_ (T_4_P6) [103]	0.5	Tris buffer (pH 8)	69	~10^4^	~10^3^	10^1^–10^2^	~10^2^	~10^4^	~10^3^	Nanofibrous networks	3D Cell Proliferation
6.	Ac-Val-Val-Lys-Cys-CONH_2_ (T_4_P7) [103]	0.5	Tris buffer (pH 8)	61	10^1^–10^2^	~10^1^	~10^−2^	~10^−1^	10^1^–10^2^	~10^1^	Nanofibrous networks	3D Cell Proliferation
7.	Nap-Phe-Glu-Phe-Lys-COOH (T_4_P8) [125]	1.51	Water (pH 2)	NR	10^2^–10^3^	NR	NR	NR	NR	NR	Nanofibrous networks	NR
		1.13	Water (pH 5)	NR	10^3^–10^4^	NR	NR	NR	NR	NR	Nanofibrous networks	NR
		0.75	Water (pH 6)	NR	~10^4^	NR	NR	NR	NR	NR	Nanofibrous networks	NR
		0.75	Water (pH 7)	NR	~10^4^	~10^3^	10^2^–10^3^	10^2^–10^3^	~10^4^	~10^3^	Nanofibrous networks	NR
		0.75	Water (pH 8)	NR	10^4^–10^5^	NR	NR	NR	NR	NR	Nanofibrous networks	NR
		1.51	Water (pH 10)	NR	10^3^–10^4^	NR	NR	NR	NR	NR	Nanofibrous networks	NR
		1.88	Water (pH 12)	NR	10^3^–10^4^	NR	NR	NR	NR	NR	Nanofibrous networks	NR
8.	NH_2_-Lys-Leu-Ile-Ile-COOH (T_4_P9) [126]	NA	Water	NA	NA	NA	NA	NA	NA	NA	NA	NA
9.	NH_2_-Lys-Leu-Ile-Ile-COOH (T_4_P10) [126]	0.5%	Water	NR	~3 × 10^4^	~9 × 10^3^	NR	NR	NR	NR	NR	Antibacterial activity
10.	NH_2_-Lys-Leu-Ile-Ile-COOH (T_4_P11) [126]	NA	Water	NA	NA	NA	NA	NA	NA	NA	NA	NA
11.	Ca-Lys-Leu-Ile-Ile-COOH (T4P12) [126]	NA	Water	NR	NA	NA	NA	NA	NA	NA	NA	NA

MGC: minimum gelation concentration; wt%: mg/100 μL; T_gel_: gel transition temperature; G′: storage modulus; G″: loss modulus; Nap: naphthoic acid; Gly: glycine; Phe: phenylalanine; Tyr: tyrosine; GlCN: glucosamine; His: histidine; Cys: cysteine; Ac: acetyl; Ile: isoleucine; PBS: phosphate buffer saline; Tris: tris(hydroxymethyl)aminomethane; NA: not applicable; NR: not reported.

**Table 6:** List of co-assembled hydrogelators and respective gelation parameters.

SL No.	Peptide Sequence	MGC (wt%)	Gelation Media	T_gel_ (°C)	Moduli (Before Mechanical Stress)		Moduli (After Mechanical Stress)		Moduli (Stress-Free Conditions)		Morphology	Application
					G′	G″	G′	G″	G′	G″		
1.	Fmoc-Arg-Gly-Asp-Ser-COOH(T_4_P13) [138]/	2.5	Water	NR	NR	NR	NR	NR	NR	NR	NR	NR
	Fmoc-Arg-Gly-Asp-Ser-COOH (T_4_P14) [138]	0.25		NR	NR	NR	~10^−2^	NR	10^0^–10^1^	NR	Cross-linked Fibrous network	Payloads release
2.	Nap-Gly-Phe-Phe-Tyr (H_2_PO_3_)-OH/ SF [149]	0.08 0.1	Water (pH 7.4)	NR	10^3^–10^4^	~10^3^	~10^1^	~10^2^	10^3^–10^4^	10^2^–10^3^	Entangled nanofibrous networks	Bone regeneration
3.	Py-Phe-Phe-Lys-CONH_2_ (T3P3)/ NDTA [5]	1.0	Water	∼64	10^3^–10^4^	10^2^–10^3^	~10^0^	~10^1^	~10^4^	10^2^–10^3^	Sheetlike structures	NR
4.	Cbz-Phe-Leu-COOH/ Lipase 1 [17]	2.06	Water (pH 7.4)	NR	10^3^–10^4^	~10^3^	~10^2^	~10^2^	10^3^–10^4^	10^2^–10^3^	Nanofibrous networks	NR
	Cbz-Phe-Leu-COOH/Lipase 2 [17]	2.06	Water (pH 7.4)	NR	~10^4^	~10^3^	~10^2^	~10^2^	~10^4^	~10^3^	Nanofibrous networks	NR
	Cbz-Phe-Leu-COOH/Thermolysin [17]	2.06	Water (pH 7.4)	NR	10^3^–10^4^	10^2^–10^3^	~10^2^	~10^2^	10^3^–10^4^	10^2^–10^3^	Nanofibrous networks	NR
5.	Cbz-Phe-Val-COOH/ Lipase 1 [17]	3.19	Water (pH 7.4)	NR	~10^3^	~10^2^	NR	NR	NR	NR	Nanofibrous networks	NR
	Cbz-Phe-Val-COOH /Lipase 2 [17]	3.19	Water (pH 7.4	NR	NR	NR	NR	NR	NR	NR	NR	NR
	Cbz-Phe-Val-COOH/Thermolysin [17]	3.98	Water (pH 7.4	NR	NR	NR	NR	NR	NR	NR	NR	NR
6.	Cbz-Phe-Ile-COOH/ Lipase 1 [17]	4.26	Water (pH 7.4)	NR	10^2^–10^3^	10^1^–10^2^	NR	NR	NR	NR	Nanofibrous networks	NR
	Cbz-Phe-Ile-COOH/ Lipase 2 [17]	NR	NR	NR	NR	NR	NR	NR	NR	NR	NR	NR
	Cbz-Phe-Ile-COOH/ Thermolysin [17]	NR	NR	NR	NR	NR	NR	NR	NR	NR	NR	NR
7.	Cbz-Phe-Tyr-COOH/ Lipase 1 [17]	3.7	Water (pH 7.4)	NR	~10^3^	~10^2^	NR	NR	NR	NR	Nanofibrous networks	NR
	Cbz-Phe-Tyr-COOH Lipase 2 [17]	NR	NR	NR	NR	NR	NR	NR	NR	NR	NR	NR
	Cbz-Phe-Tyr-COOH/Thermolysin [17]	NR	NR	NR	NR	NR	NR	NR	NR	NR	NR	NR
8.	NH_2_-Leu-Phe-COOH/ N-Form-Met-Leu-Phe-COOH [150]	1 0.25	Sodium acetate buffer (pH 7.4)	NR	~200	0–50	~1	~1	~200	0–50	Nanofibrous networks	Wound dressing
9.	Amoc-Phe-Phe-COOH/β-CD [151]	20 mM	Water (pH 7.4)	NR	~10^2^	10^1^–10^2^	10^0^–10^2^	~10^1^	~10^2^	10^1^–10^2^	Entangled fibrillar networks	Wound healing

MGC: minimum gelation concentration; wt%: mg/100 μL; T_gel_: gel transition temperature; G′: storage modulus; G″: loss modulus; Nap: naphthoic acid; Gly: glycine; Phe: phenylalanine; Tyr: tyrosine; GlCN: glucosamine; His: histidine; Cys: cysteine; Ac: acetyl; Ile: isoleucine; PBS: phosphate buffer saline; Tris: tris(hydroxymethyl)aminomethane; NA: not applicable; NR: not reported.

**Table 7:** List of composite hydrogelators and respective gelation parameters.

SL No.	Peptide Sequence	MGC (wt%)	Gelation Media	T_gel_ (°C)	Moduli (Before Mechanical Stress, Pa)		Moduli (After Mechanical Stress, Pa)		Moduli (Stress-Free Conditions, Pa)		Morphology	Application
					G′	G″	G′	G″	G′	G″		
1.	Fmoc-Phe-Phe-COOH/PLL-SH	0.4	Na_2_B_4_O_7_-EDTA buffer	NR	~10^3^	~10^2^	~10^1^	~10^1^	~10^3^	~10^2^	Nanofibrous networks	Antitumor Immune Response
2.	Fmoc-Phe-Phe-COOH/PLL	0.4	Tris-HCl buffer (pH 7.8)	NR	~10^3^	~10^2^	~10^1^	~10^1^	~10^2^	~10^1^	Nanofibrous networks	Antitumor Photodynamic Therapy
3.	Fmoc-Phe-Phe-COOH/C_60_-PTC	0.4	Water	NR	~10^4^	~10^3^	NR	NR	~10^4^	~10^3^	Nanofibrous Networks and dispersed nanoparticles	Antibacterial therapy
4.	Fmoc-Phe-Phe-COOH/Aniline	0.5	Water	NR	~10^6^	~10^5^	~10^2^	~10^4^	~10^6^	~10^5^	Fibrous networks	Pressure Sensor and Electrogenic Cell Soft Substrate
5.	Fmoc-Arg-Gly-Asp-COOH/Chitosan	1.0	Water	NR	~10^4^	~10^3^	~10^1^	~10^1^	~10^4^	~10^3^	Nanofibrous networks	Antibacterial activity
6.	Boc-D-Phe-γ4-L-Phe-PEA/Chitosan	1.0	Water:DMSO (50:50, *v*/*v*)	NR	10^4^–10^5^	10^3^–10^4^	10^2^–10^3^	~10^2^	~10^4^	~10^3^	Porous networks with lettuce-like morphology	Antibacterial activity
7.	Boc-L-Phe-γ4-L-Phe-PEA/Chitosan	1.0	Water:DMSO (50:50, *v*/*v*)	NR	~10^4^	~10^3^	~10^2^	~10^2^	~10^4^	~10^3^	Porous networks with lettuce-like morphology	Antibacterial activity
8.	Fmoc-Phe-COOH/Dextran	0.6	PBS (pH 7.4)	NR	~10^3^	10^2^–10^3^	~10^1^	~10^1^	10^3^–10^4^	~10^3^	Fibrous networks	NR
9.	Py-Lys-Cys-CONH_2_/Alg	1.0	Tris-buffer (pH 8.0)	80	~10^3^	10^2^–10^3^	~10^0^	~10^1^	~10^3^	10^2^–10^3^	Bundle-like morphology	Osteogenesis
10.	Py-Lys-Cys-CONH_2_/Chitosan	1.0	Tris-buffer (pH 8.0)	72	~10^2^	~10^2^	~10^1^	10^1^–10^2^	~10^2^	~10^2^	Entangled fibrillar networks	Osteogenesis
11.	Py-Lys-Cys-CONH_2_/Gelatin	1.0	Tris-buffer (pH 8.0)	~90	10^3^–10^4^	~10^3^	10^0^–10^−1^	~10^1^	10^3^–10^4^	~10^3^	Bundle-like morphology	Osteogenesis
12.	Py-Lys-Cys-CONH_2_/HA	1.0	Tris-buffer (pH 8.0)	~90	~10^4^	~10^3^	~10^0^	~10^1^	~10^4^	~10^3^	Brick-like structures	Osteogenesis

MGC: minimum gelation concentration; wt%: mg/100 μL; T_gel_: gel transition temperature; G′: storage modulus; G″: loss modulus; Fmoc: fluorenyl-9-methoxycarbonyl; Phe: phenylalanine; EDTA: ethylenediaminetetraacetic acid; PLL: poly-L-lysine; (Ce6): chlorin e6; Tris: tris(hydroxymethyl)aminomethane; C_60_-PTC: C60 pyrrolidine tris–acid; Arg: arginine; Gly: glycine; Boc: tert-butyloxycarbonyl; DMSO: dimethyl sulfoxide; PBS: phosphate buffer saline; Lys: lysine; Cys: cysteine.

## References

[1] Yang L., Li H., Yao L., Yu Y., Ma G. (2019). Amyloid-Based Injectable Hydrogel Derived from Hydrolyzed Hen Egg White Lysozyme. ACS Omega.

[2] Arokianathan J.F., Ramya K.A., Janeena A., Deshpande A.P., Ayyadurai N., Leemarose A., Shanmugam G. (2020). Non-proteinogenic amino acid based supramolecular hydrogel material for enhanced cell proliferation. Colloids Surf. B Biointerfaces.

[3] Dowari P., Pramanik B., Das D. (2020). pH and secondary structure instructed aggregation to a thixotropic hydrogel by a peptide amphiphile. Bull. Mater. Sci..

[4] Kuddushi M., Pandey D.K., Singh D.K., Mata J., Malek N. (2022). An ionic hydrogel with stimuli-responsive, self-healable and injectable characteristics for the targeted and sustained delivery of doxorubicin in the treatment of breast cancer. Mater. Adv..

[5] Pramanik B., Ahmed S., Singha N., Das B.K., Dowari P., Das D. (2019). Unorthodox Combination of Cation−π and Charge-Transfer Interactions within a Donor–Acceptor Pair. Langmuir.

[6] Basu K., Nandi N., Mondal B., Dehsorkhi A., Hamley I.W., Banerjee A. (2017). Peptide-based ambidextrous bifunctional gelator: Applications in oil spill recovery and removal of toxic organic dyes for waste water management. Interface Focus.

[7] Zhuang P., Greenberg Z., He M. (2021). Biologically Enhanced Starch Bio-Ink for Promoting 3D Cell Growth. Adv. Mater. Technol..

[8] Jain M., Matsumura K. (2016). Thixotropic injectable hydrogel using a polyampholyte and nanosilicate prepared directly after cryopreservation. Mater. Sci. Eng. C.

[9] Reddy S.M.M., Shanmugam G., Duraipandy N., Kiran M.S., Mandal A.B. (2015). An additional fluorenylmethoxycarbonyl (Fmoc) moiety in di-Fmoc-functionalized l-lysine induces pH-controlled ambidextrous gelation with significant advantages. Soft Matter.

[10] Shaheen A., Maswal M., Dar A.A. (2021). Synergistic effect of various metal ions on the mechanical, thixotropic, self-healing, swelling and water retention properties of bimetallic hydrogels of alginate. Colloids Surf. A Physicochem. Eng. Asp..

[11] Giuri D., Jurković L., Fermani S., Kralj D., Falini G., Tomasini C. (2019). Supramolecular Hydrogels with Properties Tunable by Calcium Ions: A Bio-Inspired Chemical System. ACS Appl. Bio Mater..

[12] Thakur N., Chaudhary A., Chakraborty A., Kumar R., Sarma T.K. (2021). Ion Conductive Phytic Acid-G Quadruplex Hydrogel as Electrolyte for Flexible Electrochromic Device. ChemNanoMat.

[13] Wang X., Song R., Johnson M., Sigen A., He Z., Milne C., Wang X., Lara-Sáez I., Xu Q., Wang W. (2021). An Injectable Chitosan-Based Self-Healable Hydrogel System as an Antibacterial Wound Dressing. Materials.

[14] Arokianathan J.F., Ramya K.A., Deshpande A.P., Leemarose A., Shanmugam G. (2021). Supramolecular organogel based on di-Fmoc functionalized unnatural amino acid: An attempt to develop a correlation between molecular structure and ambidextrous gelation. Colloids Surf. A Physicochem. Eng. Asp..

[15] Nelson C., Tuladhar S., Launen L., Habib A. (2021). 3D Bio-Printability of Hybrid Pre-Crosslinked Hydrogels. Int. J. Mol. Sci..

[16] Marić I., Šijaković Vujičić N., Pustak A., Gotić M., Štefanić G., Grenèche J.-M., Dražić G., Jurkin T. (2020). Rheological, Microstructural and Thermal Properties of Magnetic Poly(Ethylene Oxide)/Iron Oxide Nanocomposite Hydrogels Synthesized Using a One-Step Gamma-Irradiation Method. Nanomaterials.

[17] Jain R., Pal V.K., Roy S. (2020). Triggering Supramolecular Hydrogelation Using a Protein–Peptide Coassembly Approach. Biomacromolecules.

[18] Yan C., Pochan D.J. (2010). Rheological properties of peptide-based hydrogels for biomedical and other applications. Chem. Soc. Rev..

[19] Mondal S., Das S., Nandi A.K. (2020). A review on recent advances in polymer and peptide hydrogels. Soft Matter.

[20] Das S., Das D. (2021). Rational Design of Peptide-based Smart Hydrogels for Therapeutic Applications. Front. Chem..

[21] Ni M., Zhuo S. (2019). Applications of self-assembling ultrashort peptides in bionanotechnology. RSC Adv..

[22] Habibi N., Kamaly N., Memic A., Shafiee H. (2016). Self-assembled peptide-based nanostructures: Smart nanomaterials toward targeted drug delivery. Nano Today.

[23] Mayr J., Saldías C., Díaz Díaz D. (2018). Release of small bioactive molecules from physical gels. Chem. Soc. Rev..

[24] Liu C., Zhang Q., Zhu S., Liu H., Chen J. (2019). Preparation and applications of peptide-based injectable hydrogels. RSC Adv..

[25] Bertsch P., Diba M., Mooney D.J., Leeuwenburgh S.C.G. (2022). Self-Healing Injectable Hydrogels for Tissue Regeneration. Chem. Rev..

[26] Dasgupta A., Mondal J.H., Das D. (2013). Peptide hydrogels. RSC Adv..

[27] Pramanik B., Ahmed S. (2022). Peptide-Based Low Molecular Weight Photosensitive Supramolecular Gelators. Gels.

[28] Correa S., Grosskopf A.K., Lopez Hernandez H., Chan D., Yu A.C., Stapleton L.M., Appel E.A. (2021). Translational Applications of Hydrogels. Chem. Rev..

[29] Yadav N., Chauhan M.K., Chauhan V.S. (2020). Short to ultrashort peptide-based hydrogels as a platform for biomedical applications. Biomater. Sci..

[30] Gačanin J., Hedrich J., Sieste S., Glaßer G., Lieberwirth I., Schilling C., Fischer S., Barth H., Knöll B., Synatschke C.V. (2019). Autonomous Ultrafast Self-Healing Hydrogels by pH-Responsive Functional Nanofiber Gelators as Cell Matrices. Adv. Mater..

[31] Cai L., Liu S., Guo J., Jia Y.-G. (2020). Polypeptide-based self-healing hydrogels: Design and biomedical applications. Acta Biomater..

[32] Zhang Y., Song W., Lu Y., Xu Y., Wang C., Yu D.-G., Kim I. (2022). Recent Advances in Poly(α-L-glutamic acid)-Based Nanomaterials for Drug Delivery. Biomolecules.

[33] Zanna N., Tomasini C. (2017). Peptide-Based Physical Gels Endowed with Thixotropic Behaviour. Gels.

[34] Wahid F., Zhou Y.-N., Wang H.-S., Wan T., Zhong C., Chu L.-Q. (2018). Injectable self-healing carboxymethyl chitosan-zinc supramolecular hydrogels and their antibacterial activity. Int. J. Biol. Macromol..

[35] Fichman G., Gazit E. (2014). Self-assembly of short peptides to form hydrogels: Design of building blocks, physical properties and technological applications. Acta Biomater..

[36] Jervis P.J., Amorim C., Pereira T., Martins J.A., Ferreira P.M.T. (2020). Exploring the properties and potential biomedical applications of NSAID-capped peptide hydrogels. Soft Matter.

[37] Nolan M.C., Fuentes Caparrós A.M., Dietrich B., Barrow M., Cross E.R., Bleuel M., King S.M., Adams D.J. (2017). Optimising low molecular weight hydrogels for automated 3D printing. Soft Matter.

[38] Shao T., Falcone N., Kraatz H.-B. (2020). Supramolecular Peptide Gels: Influencing Properties by Metal Ion Coordination and Their Wide-Ranging Applications. ACS Omega.

[39] Afzal S., Maswal M., Lone M.S., Ashraf U., Mushtaq U., Dar A.A. (2021). Metal–ligand-based thixotropic self-healing poly (vinyl alcohol) metallohydrogels: Their application in pH-responsive drug release and selective adsorption of dyes. J. Mater. Res..

[40] Singha N., Srivastava A., Pramanik B., Ahmed S., Dowari P., Chowdhuri S., Das B.K., Debnath A., Das D. (2019). Unusual confinement properties of a water insoluble small peptide hydrogel. Chem. Sci..

[41] Saydé T., El Hamoui O., Alies B., Gaudin K., Lespes G., Battu S. (2021). Biomaterials for Three-Dimensional Cell Culture: From Applications in Oncology to Nanotechnology. Nanomaterials.

[42] Antimicrobial Agent. https://physicsworld.com/a/nanotechnology-takes-on-microbial-drug-resistance/.

[43] Drug-Delivery. https://www.aiche.org/chenected/2014/02/evolution-drug-delivery.

[44] Atherosclerosis. https://www.thehealthsite.com/diseases-conditions/atherosclerosis/.

[45] Wound Dressing. https://www.123rf.com/clipart-vector/wound_dressing.html.

[46] Chen H., Cheng R., Zhao X., Zhang Y., Tam A., Yan Y., Shen H., Zhang Y.S., Qi J., Feng Y. (2019). An injectable self-healing coordinative hydrogel with antibacterial and angiogenic properties for diabetic skin wound repair. NPG Asia Mater..

[47] Tissue Engineering. https://www.nist.gov/programs-projects/tissue-engineering.

[48] Liu M., Zeng X., Ma C., Yi H., Ali Z., Mou X., Li S., Deng Y., He N. (2017). Injectable hydrogels for cartilage and bone tissue engineering. Bone Res..

[49] Zhou Z., Samperi M., Santu L., Dizon G., Aboarkaba S., Limón D., Tuck C., Pérez-García L., Irvine D.J., Amabilino D.B. (2021). An imidazolium-based supramolecular gelator enhancing interlayer adhesion in 3D printed dual network hydrogels. Mater. Des..

[50] Zou Q., Chang R., Xing R., Yuan C., Yan X. (2020). Injectable self-assembled bola-dipeptide hydrogels for sustained photodynamic prodrug delivery and enhanced tumor therapy. J. Control. Release.

[51] Panwar V., Babu A., Sharma A., Thomas J., Chopra V., Malik P., Rajput S., Mittal M., Guha R., Chattopadhyay N. (2021). Tunable, conductive, self-healing, adhesive and injectable hydrogels for bioelectronics and tissue regeneration applications. J. Mater. Chem. B.

[52] Adak A., Das G., Khan J., Mukherjee N., Gupta V., Mallesh R., Ghosh S. (2020). Extracellular Matrix (ECM)-Mimicking Neuroprotective Injectable Sulfo-Functionalized Peptide Hydrogel for Repairing Brain Injury. ACS Biomater. Sci. Eng..

[53] Raymond D.M., Abraham B.L., Fujita T., Watrous M.J., Toriki E.S., Takano T., Nilsson B.L. (2019). Low-Molecular-Weight Supramolecular Hydrogels for Sustained and Localized in Vivo Drug Delivery. ACS Appl. Bio Mater..

[54] Reddy S.M.M., Augustine G., Ayyadurai N., Shanmugam G. (2018). Biocytin-Based pH-Stimuli Responsive Supramolecular Multivariant Hydrogelator for Potential Applications. ACS Appl. Bio Mater..

[55] Das A.K., Gavel P.K. (2020). Low molecular weight self-assembling peptide-based materials for cell culture, antimicrobial, anti-inflammatory, wound healing, anticancer, drug delivery, bioimaging and 3D bioprinting applications. Soft Matter.

[56] Singha N., Gupta P., Pramanik B., Ahmed S., Dasgupta A., Ukil A., Das D. (2017). Hydrogelation of a Naphthalene Diimide Appended Peptide Amphiphile and Its Application in Cell Imaging and Intracellular pH Sensing. Biomacromolecules.

[57] Pramanik B., Singha N., Das D. (2019). Sol-, Gel-, and Paper-Based Detection of Picric Acid at Femtogram Level by a Short Peptide Gelator. ACS Appl. Polym. Mater..

[58] Abraham B.L., Liyanage W., Nilsson B.L. (2019). Strategy to Identify Improved N-Terminal Modifications for Supramolecular Phenylalanine-Derived Hydrogelators. Langmuir.

[59] La Manna S., Di Natale C., Onesto V., Marasco D. (2021). Self-Assembling Peptides: From Design to Biomedical Applications. Int. J. Mol. Sci..

[60] Ahmed S., Mondal J.H., Behera N., Das D. (2013). Self-Assembly of Peptide-Amphiphile Forming Helical Nanofibers and in Situ Template Synthesis of Uniform Mesoporous Single Wall Silica Nanotubes. Langmuir.

[61] Singh P., Misra S., Das A., Roy S., Datta P., Bhattacharjee G., Satpati B., Nanda J. (2019). Supramolecular Hydrogel from an Oxidized Byproduct of Tyrosine. ACS Appl. Bio Mater..

[62] Falcone N., Shao T., Rashid R., Kraatz H.-B. (2019). Enzyme Entrapment in Amphiphilic Myristyl-Phenylalanine Hydrogels. Molecules.

[63] Chakraborty P., Gazit E. (2018). Amino Acid Based Self-assembled Nanostructures: Complex Structures from Remarkably Simple Building Blocks. ChemNanoMat.

[64] Liyanage W., Nilsson B.L. (2016). Substituent Effects on the Self-Assembly/Coassembly and Hydrogelation of Phenylalanine Derivatives. Langmuir.

[65] Martí-Centelles R., Escuder B. (2018). Morphology Diversity of L-Phenylalanine-Based Short Peptide Supramolecular Aggregates and Hydrogels. ChemNanoMat.

[66] Aviv M., Cohen-Gerassi D., Orr A.A., Misra R., Arnon Z.A., Shimon L.J.W., Shacham-Diamand Y., Tamamis P., Adler-Abramovich L. (2021). Modification of a Single Atom Affects the Physical Properties of Double Fluorinated Fmoc-Phe Derivatives. Int. J. Mol. Sci..

[67] Singh V., Snigdha K., Singh C., Sinha N., Thakur A.K. (2015). Understanding the self-assembly of Fmoc–phenylalanine to hydrogel formation. Soft Matter.

[68] Quigley E., Johnson J., Liyanage W., Nilsson B.L. (2020). Impact of gelation method on thixotropic properties of phenylalanine-derived supramolecular hydrogels. Soft Matter.

[69] Ramya K.A., Reddy S.M.M., Shanmugam G., Deshpande A.P. (2020). Fibrillar Network Dynamics during Oscillatory Rheology of Supramolecular Gels. Langmuir.

[70] Rajbhandary A., Brennessel W.W., Nilsson B.L. (2018). Comparison of the Self-Assembly Behavior of Fmoc-Phenylalanine and Corresponding Peptoid Derivatives. Cryst. Growth Des..

[71] Du X., Zhou J., Shi J., Xu B. (2015). Supramolecular Hydrogelators and Hydrogels: From Soft Matter to Molecular Biomaterials. Chem. Rev..

[72] Nandi N., Gayen K., Ghosh S., Bhunia D., Kirkham S., Sen S.K., Ghosh S., Hamley I.W., Banerjee A. (2017). Amphiphilic Peptide-Based Supramolecular, Noncytotoxic, Stimuli-Responsive Hydrogels with Antibacterial Activity. Biomacromolecules.

[73] Nandi S.K., Maji K., Haldar D. (2018). Self-Healing Hydrogel from a Dipeptide and HCl Sensing. ACS Omega.

[74] Colquhoun C., Draper E.R., Schweins R., Marcello M., Vadukul D., Serpell L.C., Adams D.J. (2017). Controlling the network type in self-assembled dipeptide hydrogels. Soft Matter.

[75] Brown N., Lei J., Zhan C., Shimon L.J.W., Adler-Abramovich L., Wei G., Gazit E. (2018). Structural Polymorphism in a Self-Assembled Tri-Aromatic Peptide System. ACS Nano.

[76] Jain R., Khandelwal G., Roy S. (2019). Unraveling the Design Rules in Ultrashort Amyloid-Based Peptide Assemblies toward Shape-Controlled Synthesis of Gold Nanoparticles. Langmuir.

[77] Kaur H., Jain R., Roy S. (2020). Pathway-Dependent Preferential Selection and Amplification of Variable Self-Assembled Peptide Nanostructures and Their Biological Activities. ACS Appl. Mater. Interfaces.

[78] Tong Q., Zhang L., Li Y., Li B., Yang Y. (2018). Alignment of twisted nanoribbons formed by C17H35CO-Val-Ala sodium salts. Soft Matter.

[79] Gavel P.K., Dev D., Parmar H.S., Bhasin S., Das A.K. (2018). Investigations of Peptide-Based Biocompatible Injectable Shape-Memory Hydrogels: Differential Biological Effects on Bacterial and Human Blood Cells. ACS Appl. Mater. Interfaces.

[80] Falcone N., Shao T., Andoy N.M.O., Rashid R., Sullan R.M.A., Sun X., Kraatz H.-B. (2020). Multi-component peptide hydrogels—A systematic study incorporating biomolecules for the exploration of diverse, tuneable biomaterials. Biomater. Sci..

[81] Mondal B., Bairagi D., Nandi N., Hansda B., Das K.S., Edwards-Gayle C.J.C., Castelletto V., Hamley I.W., Banerjee A. (2020). Peptide-Based Gel in Environmental Remediation: Removal of Toxic Organic Dyes and Hazardous Pb^2+^ and Cd^2+^ Ions from Wastewater and Oil Spill Recovery. Langmuir.

[82] Das B.K., Pramanik B., Chowdhuri S., Scherman O.A., Das D. (2020). Light-triggered syneresis of a water insoluble peptide-hydrogel effectively removes small molecule waste contaminants. Chem. Commun..

[83] Chakraborty P., Tang Y., Yamamoto T., Yao Y., Guterman T., Zilberzwige-Tal S., Adadi N., Ji W., Dvir T., Ramamoorthy A. (2020). Unusual Two-Step Assembly of a Minimalistic Dipeptide-Based Functional Hypergelator. Adv. Mater..

[84] Ren P., Li J., Zhao L., Wang A., Wang M., Li J., Jian H., Li X., Yan X., Bai S. (2020). Dipeptide Self-assembled Hydrogels with Shear-Thinning and Instantaneous Self-healing Properties Determined by Peptide Sequences. ACS Appl. Mater. Interfaces.

[85] Fu W., Farhadi Sabet Z., Liu J., You M., Zhou H., Wang Y., Gao Y., Li J., Ma X., Chen C. (2020). Metal ions modulation of the self-assembly of short peptide conjugated nonsteroidal anti-inflammatory drugs (NSAIDs). Nanoscale.

[86] Kaur H., Roy S. (2021). Enzyme-Induced Supramolecular Order in Pyrene Dipeptide Hydrogels for the Development of an Efficient Energy-Transfer Template. Biomacromolecules.

[87] Najafi H., Tamaddon A.M., Abolmaali S., Borandeh S., Azarpira N. (2021). Structural, mechanical, and biological characterization of hierarchical nanofibrous Fmoc-phenylalanine-valine hydrogels for 3D culture of differentiated and mesenchymal stem cells. Soft Matter.

[88] Ahmed S., Pramanik B., Sankar K.N.A., Srivastava A., Singha N., Dowari P., Srivastava A., Mohanta K., Debnath A., Das D. (2017). Solvent Assisted Tuning of Morphology of a Peptide-Perylenediimide Conjugate: Helical Fibers to Nano-Rings and their Differential Semiconductivity. Sci. Rep..

[89] Ahmed S., Amba Sankar K.N., Pramanik B., Mohanta K., Das D. (2018). Solvent Directed Morphogenesis and Electrical Properties of a Peptide–Perylenediimide Conjugate. Langmuir.

[90] Marchesan S., Vargiu A.V., Styan K.E. (2015). The Phe-Phe Motif for Peptide Self-Assembly in Nanomedicine. Molecules.

[91] Dasgupta A., Das D. (2019). Designer Peptide Amphiphiles: Self-Assembly to Applications. Langmuir.

[92] Levin A., Hakala T.A., Schnaider L., Bernardes G.J.L., Gazit E., Knowles T.P.J. (2020). Biomimetic peptide self-assembly for functional materials. Nat. Rev. Chem..

[93] Arakawa H., Takeda K., Higashi S.L., Shibata A., Kitamura Y., Ikeda M. (2020). Self-assembly and hydrogel formation ability of Fmoc-dipeptides comprising α-methyl-L-phenylalanine. Polym. J..

[94] Smith A.M., Williams R.J., Tang C., Coppo P., Collins R.F., Turner M.L., Saiani A., Ulijn R.V. (2008). Fmoc-Diphenylalanine Self Assembles to a Hydrogel via a Novel Architecture Based on π–π Interlocked β-Sheets. Adv. Mater..

[95] Tamamis P., Adler-Abramovich L., Reches M., Marshall K., Sikorski P., Serpell L., Gazit E., Archontis G. (2009). Self-Assembly of Phenylalanine Oligopeptides: Insights from Experiments and Simulations. Biophys. J..

[96] Adler-Abramovich L., Gazit E. (2014). The physical properties of supramolecular peptide assemblies: From building block association to technological applications. Chem. Soc. Rev..

[97] Fan T., Yu X., Shen B., Sun L. (2017). Peptide Self-Assembled Nanostructures for Drug Delivery Applications. J. Nanomater..

[98] Ji W., Tang Y., Makam P., Yao Y., Jiao R., Cai K., Wei G., Gazit E. (2021). Expanding the Structural Diversity and Functional Scope of Diphenylalanine-Based Peptide Architectures by Hierarchical Coassembly. J. Am. Chem. Soc..

[99] Bian S., Cai H., Cui Y., He M., Cao W., Chen X., Sun Y., Liang J., Fan Y., Zhang X. (2017). Temperature and ion dual responsive biphenyl-dipeptide supramolecular hydrogels as extracellular matrix mimic-scaffolds for cell culture applications. J. Mater. Chem. B.

[100] Biswas S., Rasale D.B., Das A.K. (2016). Blue light emitting self-healable graphene quantum dot embedded hydrogels. RSC Adv..

[101] Pramanik B., Das D. (2018). Aggregation-Induced Emission or Hydrolysis by Water? The Case of Schiff Bases in Aqueous Organic Solvents. J. Phys. Chem. C.

[102] Singha N., Das B.K., Pramanik B., Das S., Das D. (2019). Freeze the dynamicity: Charge transfer complexation assisted control over the reaction pathway. Chem. Sci..

[103] Chowdhuri S., Saha A., Pramanik B., Das S., Dowari P., Ukil A., Das D. (2020). Smart Thixotropic Hydrogels by Disulfide-Linked Short Peptides for Effective Three-Dimensional Cell Proliferation. Langmuir.

[104] Najafi H., Abolmaali S.S., Heidari R., Valizadeh H., Jafari M., Tamaddon A.M., Azarpira N. (2021). Nitric oxide releasing nanofibrous Fmoc-dipeptide hydrogels for amelioration of renal ischemia/reperfusion injury. J. Control. Release.

[105] Wang L., Jin X., Ye L., Zhang A.-y., Bezuidenhout D., Feng Z.-g. (2017). Rapidly Recoverable Thixotropic Hydrogels from the Racemate of Chiral OFm Monosubstituted Cyclo(Glu-Glu) Derivatives. Langmuir.

[106] You Y., Xing R., Zou Q., Shi F., Yan X. (2019). High-tolerance crystalline hydrogels formed from self-assembling cyclic dipeptide. Beilstein J. Nanotechnol..

[107] Scarel M., Marchesan S. (2021). Diketopiperazine Gels: New Horizons from the Self-Assembly of Cyclic Dipeptides. Molecules.

[108] Yang M., Xing R., Shen G., Yuan C., Yan X. (2019). A versatile cyclic dipeptide hydrogelator: Self-assembly and rheology in various physiological conditions. Colloids Surf. A Physicochem. Eng. Asp..

[109] Zhao K., Xing R., Yan X. (2021). Cyclic dipeptides: Biological activities and self-assembled materials. Pept. Sci..

[110] Choi S.-j., Jeong W.-j., Kang S.-K., Lee M., Kim E., Ryu D.Y., Lim Y.-b. (2012). Differential Self-Assembly Behaviors of Cyclic and Linear Peptides. Biomacromolecules.

[111] Wojciechowski J.P., Martin A.D., Mason A.F., Fife C.M., Sagnella S.M., Kavallaris M., Thordarson P. (2017). Choice of Capping Group in Tripeptide Hydrogels Influences Viability in the Three-Dimensional Cell Culture of Tumor Spheroids. ChemPlusChem.

[112] Ou C., Zhang J., Zhang X., Yang Z., Chen M. (2013). Phenothiazine as an aromatic capping group to construct a short peptide-based ‘super gelator’. Chem. Commun..

[113] Bairagi D., Biswas P., Basu K., Hazra S., Hermida-Merino D., Sinha D.K., Hamley I.W., Banerjee A. (2019). Self-Assembling Peptide-Based Hydrogel: Regulation of Mechanical Stiffness and Thermal Stability and 3D Cell Culture of Fibroblasts. ACS Appl. Bio Mater..

[114] Yang X., Wang Y., Qi W., Xing R., Yang X., Xing Q., Su R., He Z. (2019). Disulfide crosslinking and helical coiling of peptide micelles facilitate the formation of a printable hydrogel. J. Mater. Chem. B.

[115] Chauhan N., Singh Y. (2020). Self-Assembled Fmoc-Arg-Phe-Phe Peptide Gels with Highly Potent Bactericidal Activities. ACS Biomater. Sci. Eng..

[116] Li X., Bian S., Zhao M., Han X., Liang J., Wang K., Jiang Q., Sun Y., Fan Y., Zhang X. (2021). Stimuli-responsive biphenyl-tripeptide supramolecular hydrogels as biomimetic extracellular matrix scaffolds for cartilage tissue engineering. Acta Biomater..

[117] Sun Y., Li X., Zhao M., Chen Y., Xu Y., Wang K., Bian S., Jiang Q., Fan Y., Zhang X. (2022). Bioinspired supramolecular nanofiber hydrogel through self-assembly of biphenyl-tripeptide for tissue engineering. Bioact. Mater..

[118] Tiwari P., Gupta A., Shukla D.N., Mishra A.K., Basu A., Dutt Konar A. (2021). Chiral Orchestration: A Tool for Fishing Out Tripeptide-Based Mechanoresponsive Supergelators Possessing Anti-Inflammatory and Antimicrobial Properties. ACS Appl. Bio Mater..

[119] Zhang R., Zhou J., Lin D., Hu Y., Jin B., Wang Y., Wang J., Vakal S., Li X. (2021). Dexamethasone-peptide prodrug supramolecular hydrogel effectively alleviates experimental autoimmune uveitis (EAU). Chem. Eng. J..

[120] Chai Q., Jiao Y., Yu X. (2017). Hydrogels for Biomedical Applications: Their Characteristics and the Mechanisms behind Them. Gels.

[121] Jonker A.M., Löwik D.W.P.M., van Hest J.C.M. (2012). Peptide- and Protein-Based Hydrogels. Chem. Mater..

[122] Luo Z., Wu Q., Yang C., Wang H., He T., Wang Y., Wang Z., Chen H., Li X., Gong C. (2017). A Powerful CD8+ T-Cell Stimulating D-Tetra-Peptide Hydrogel as a Very Promising Vaccine Adjuvant. Adv. Mater..

[123] Qi J., Yan Y., Cheng B., Deng L., Shao Z., Sun Z., Li X. (2018). Enzymatic Formation of an Injectable Hydrogel from a Glycopeptide as a Biomimetic Scaffold for Vascularization. ACS Appl. Mater. Interfaces.

[124] Zhao Y., Wang Z., Mei C., Jiang Z., Feng Y., Gao R., Wang Q., Huang J. (2018). Protein Enables Conformation Transition of a Hydrogel Based on Pentapeptide and Boosts Immune Response In Vivo. Bioconjug. Chem..

[125] Kaur H., Sharma P., Patel N., Pal V.K., Roy S. (2020). Accessing Highly Tunable Nanostructured Hydrogels in a Short Ionic Complementary Peptide Sequence via pH Trigger. Langmuir.

[126] Prasad Dewangan R., Kumari S., Kumar Mahto A., Jain A., Pasha S. (2020). Self assembly and hydrogelation of N-terminal modified tetrapeptide for sustained release and synergistic action of antibacterial drugs against methicillin resistant *S. aureus*. Bioorg. Chem..

[127] Makam P., Gazit E. (2018). Minimalistic peptide supramolecular co-assembly: Expanding the conformational space for nanotechnology. Chem. Soc. Rev..

[128] Chakraborty P., Aviv M., Netti F., Cohen-Gerassi D., Adler-Abramovich L. (2022). Molecular Co-Assembly of Two Building Blocks Harnesses Both their Attributes into a Functional Supramolecular Hydrogel. Macromol. Biosci..

[129] Wu A., Guo Y., Li X., Xue H., Fei J., Li J. (2021). Co-assembled Supramolecular Gel of Dipeptide and Pyridine Derivatives with Controlled Chirality. Angew. Chem. Int. Ed..

[130] Kim K.Y., Ok M., Kim J., Jung S.H., Seo M.L., Jung J.H. (2020). Pyrene-Based Co-Assembled Supramolecular Gel; Morphology Changes and Macroscale Mechanical Property. Gels.

[131] Giraud T., Bouguet-Bonnet S., Stébé M.-J., Richaudeau L., Pickaert G., Averlant-Petit M.-C., Stefan L. (2021). Co-assembly and multicomponent hydrogel formation upon mixing nucleobase-containing peptides. Nanoscale.

[132] Zhai Z., Xu K., Mei L., Wu C., Liu J., Liu Z., Wan L., Zhong W. (2019). Co-assembled supramolecular hydrogels of cell adhesive peptide and alginate for rapid hemostasis and efficacious wound healing. Soft Matter.

[133] Mantooth S.M., Munoz-Robles B.G., Webber M.J. (2019). Dynamic Hydrogels from Host–Guest Supramolecular Interactions. Macromol. Biosci..

[134] Liu G., Yuan Q., Hollett G., Zhao W., Kang Y., Wu J. (2018). Cyclodextrin-based host–guest supramolecular hydrogel and its application in biomedical fields. Polym. Chem..

[135] Xiong H., Li Y., Ye H., Huang G., Zhou D., Huang Y. (2020). Self-healing supramolecular hydrogels through host–guest interaction between cyclodextrin and carborane. J. Mater. Chem. B.

[136] Lu H., Hao J., Wang X. (2022). Host-Fueled Transient Supramolecular Hydrogels. ChemSystemsChem.

[137] Madl A.C., Myung D. (2021). Supramolecular Host–Guest Hydrogels for Corneal Regeneration. Gels.

[138] Chu C.-W., Ravoo B.J. (2017). Hierarchical supramolecular hydrogels: Self-assembly by peptides and photo-controlled release via host–guest interaction. Chem. Commun..

[139] Dowari P., Saha S., Pramanik B., Ahmed S., Singha N., Ukil A., Das D. (2018). Multiple Cross-Linking of a Small Peptide to Form a Size Tunable Biopolymer with Efficient Cell Adhesion and Proliferation Property. Biomacromolecules.

[140] Larik F.A., Fillbrook L.L., Nurttila S.S., Martin A.D., Kuchel R.P., Al Taief K., Bhadbhade M., Beves J.E., Thordarson P. (2021). Ultra-Low Molecular Weight Photoswitchable Hydrogelators. Angew. Chem. Int. Ed..

[141] Stricker L., Fritz E.-C., Peterlechner M., Doltsinis N.L., Ravoo B.J. (2016). Arylazopyrazoles as Light-Responsive Molecular Switches in Cyclodextrin-Based Supramolecular Systems. J. Am. Chem. Soc..

[142] Redondo-Gómez C., Abdouni Y., Becer C.R., Mata A. (2019). Self-Assembling Hydrogels Based on a Complementary Host–Guest Peptide Amphiphile Pair. Biomacromolecules.

[143] Jain M., Nowak B.P., Ravoo B.J. (2022). Supramolecular Hydrogels Based on Cyclodextrins: Progress and Perspectives. ChemNanoMat.

[144] Lange S.C., Unsleber J., Drücker P., Galla H.-J., Waller M.P., Ravoo B.J. (2015). pH response and molecular recognition in a low molecular weight peptide hydrogel. Org. Biomol. Chem..

[145] Himmelein S., Ravoo B.J. (2017). A Self-Assembled Sensor for Carbohydrates on the Surface of Cyclodextrin Vesicles. Chem. Eur. J..

[146] Volarić J., Szymanski W., Simeth N.A., Feringa B.L. (2021). Molecular photoswitches in aqueous environments. Chem. Soc. Rev..

[147] Li L., Scheiger J.M., Levkin P.A. (2019). Design and Applications of Photoresponsive Hydrogels. Adv. Mater..

[148] Jia S., Fong W.-K., Graham B., Boyd B.J. (2018). Photoswitchable Molecules in Long-Wavelength Light-Responsive Drug Delivery: From Molecular Design to Applications. Chem. Mater..

[149] Li H., Zhang J., Liu S., Yan Y., Li X. (2020). Consecutive dephosphorylation by alkaline phosphatase-directed in situ formation of porous hydrogels of SF with nanocrystalline calcium phosphate ceramics for bone regeneration. J. Mater. Chem. B.

[150] Thota C.K., Berger A.A., Elomaa L., Nie C., Böttcher C., Koksch B. (2020). Coassembly Generates Peptide Hydrogel with Wound Dressing Material Properties. ACS Omega.

[151] Gavel P.K., Kumar N., Parmar H.S., Das A.K. (2020). Evaluation of a Peptide-Based Coassembled Nanofibrous and Thixotropic Hydrogel for Dermal Wound Healing. ACS Appl. Bio Mater..

[152] Toledano S., Williams R.J., Jayawarna V., Ulijn R.V. (2006). Enzyme-Triggered Self-Assembly of Peptide Hydrogels via Reversed Hydrolysis. J. Am. Chem. Soc..

[153] Criado-Gonzalez M., Rodon Fores J., Wagner D., Schröder A.P., Carvalho A., Schmutz M., Harth E., Schaaf P., Jierry L., Boulmedais F. (2019). Enzyme-assisted self-assembly within a hydrogel induced by peptide diffusion. Chem. Commun..

[154] Dubey P., Kumar S., Aswal V.K., Ravindranathan S., Rajamohanan P.R., Prabhune A., Nisal A. (2016). Silk Fibroin-Sophorolipid Gelation: Deciphering the Underlying Mechanism. Biomacromolecules.

[155] Chouhan D., Lohe T.-u., Samudrala P.K., Mandal B.B. (2018). In Situ Forming Injectable Silk Fibroin Hydrogel Promotes Skin Regeneration in Full Thickness Burn Wounds. Adv. Healthc. Mater..

[156] Hai Z., Li J., Wu J., Xu J., Liang G. (2017). Alkaline Phosphatase-Triggered Simultaneous Hydrogelation and Chemiluminescence. J. Am. Chem. Soc..

[157] Wang Y., Zhang Y., Li X., Li C., Yang Z., Wang L. (2018). A Peptide-Based Supramolecular Hydrogel for Controlled Delivery of Amine Drugs. Chem. Asian J..

[158] Bartocci S., Berrocal J.A., Guarracino P., Grillaud M., Franco L., Mba M. (2018). Peptide-Driven Charge-Transfer Organogels Built from Synergetic Hydrogen Bonding and Pyrene–Naphthalenediimide Donor–Acceptor Interactions. Chem. Eur. J..

[159] Kobaisi M.A., Bhosale R.S., El-Khouly M.E., La D.D., Padghan S.D., Bhosale S.V., Jones L.A., Antolasic F., Fukuzumi S., Bhosale S.V. (2017). The sensitivity of donor—Acceptor charge transfer to molecular geometry in DAN—NDI based supramolecular flower-like self-assemblies. Sci. Rep..

[160] Ghosh S., Pramanik B., Das D. (2018). Self-Aggregation of a Naphthalene-Monoimide Amphiphile and Its Charge-Transfer-Complex Driven Morphogenesis in Water. ChemNanoMat.

[161] Peng X., Wang L., Chen S. (2021). Donor–acceptor charge transfer assemblies based on naphthalene diimides(NDIs). J. Incl. Phenom. Macrocycl. Chem..

[162] Nalluri S.K.M., Berdugo C., Javid N., Frederix P.W.J.M., Ulijn R.V. (2014). Biocatalytic Self-Assembly of Supramolecular Charge-Transfer Nanostructures Based on n-Type Semiconductor-Appended Peptides. Angew. Chem. Int. Ed..

[163] Nandi S.K., Sarkar R., Jaiswar A., Roy S., Haldar D. (2022). Miniature β-Hairpin Mimetic by Intramolecular Hydrogen Bond and C–H···π Interactions. ACS Omega.

[164] Sonallya T., Sruthi L., Deshpande A.P., Shanmugam G. (2021). Tweaking of supramolecular hydrogel property of single and two-component gel systems by a bifunctional molecule. J. Mol. Liq..

[165] Mahmood A., Patel D., Hickson B., DesRochers J., Hu X. (2022). Recent Progress in Biopolymer-Based Hydrogel Materials for Biomedical Applications. Int. J. Mol. Sci..

[166] Radvar E., Azevedo H.S. (2019). Supramolecular Peptide/Polymer Hybrid Hydrogels for Biomedical Applications. Macromol. Biosci..

[167] Chowdhuri S., Ghosh M., Adler-Abramovich L., Das D. (2021). The Effects of a Short Self-Assembling Peptide on the Physical and Biological Properties of Biopolymer Hydrogels. Pharmaceutics.

[168] Tang C., Smith A.M., Collins R.F., Ulijn R.V., Saiani A. (2009). Fmoc-Diphenylalanine Self-Assembly Mechanism Induces Apparent pKa Shifts. Langmuir.

[169] Shim J., Kang J., Yun S.I. (2021). Chitosan–dipeptide hydrogels as potential anticancer drug delivery systems. Int. J. Biol. Macromol..

[170] Choe R., Yun S.I. (2020). Fmoc-diphenylalanine-based hydrogels as a potential carrier for drug delivery. e-Polymers.

[171] Xing R., Li S., Zhang N., Shen G., Möhwald H., Yan X. (2017). Self-Assembled Injectable Peptide Hydrogels Capable of Triggering Antitumor Immune Response. Biomacromolecules.

[172] Abbas M., Xing R., Zhang N., Zou Q., Yan X. (2018). Antitumor Photodynamic Therapy Based on Dipeptide Fibrous Hydrogels with Incorporation of Photosensitive Drugs. ACS Biomater. Sci. Eng..

[173] Zhang Y., Zhang H., Zou Q., Xing R., Jiao T., Yan X. (2018). An injectable dipeptide–fullerene supramolecular hydrogel for photodynamic antibacterial therapy. J. Mater. Chem. B.

[174] Chakraborty P., Guterman T., Adadi N., Yadid M., Brosh T., Adler-Abramovich L., Dvir T., Gazit E. (2019). A Self-Healing, All-Organic, Conducting, Composite Peptide Hydrogel as Pressure Sensor and Electrogenic Cell Soft Substrate. ACS Nano.

[175] Ghosh M., Halperin-Sternfeld M., Grinberg I., Adler-Abramovich L. (2019). Injectable Alginate-Peptide Composite Hydrogel as a Scaffold for Bone Tissue Regeneration. Nanomaterials.

[176] Chakraborty P., Ghosh M., Schnaider L., Adadi N., Ji W., Bychenko D., Dvir T., Adler-Abramovich L., Gazit E. (2019). Composite of Peptide-Supramolecular Polymer and Covalent Polymer Comprises a New Multifunctional, Bio-Inspired Soft Material. Macromol. Rapid Commun..

[177] Das Mahapatra R., Dey J., Weiss R.G. (2019). Poly(vinyl alcohol)-induced thixotropy of an l-carnosine-based cytocompatible, tripeptidic hydrogel. Soft Matter.

[178] Malhotra K., Shankar S., Chauhan N., Rai R., Singh Y. (2020). Design, characterization, and evaluation of antibacterial gels, Boc-D-Phe-γ4-L-Phe-PEA/chitosan and Boc-L-Phe-γ4-L-Phe-PEA/chitosan, for biomaterial-related infections. Mater. Sci. Eng. C.

[179] Abraham J.N., Joseph S., Trivedi R., Karle M. (2021). Injectable dextran-fluorenylmethoxycarbonyl phenylalanine composite hydrogels with improved mechanical properties. Polym. Int..

[180] Wang L., Li J., Xiong Y., Wu Y., Yang F., Guo Y., Chen Z., Gao L., Deng W. (2021). Ultrashort Peptides and Hyaluronic Acid-Based Injectable Composite Hydrogels for Sustained Drug Release and Chronic Diabetic Wound Healing. ACS Appl. Mater. Interfaces.

[181] Li Y., Wang F., Cui H. (2016). Peptide-based supramolecular hydrogels for delivery of biologics. Bioeng. Transl. Med..

[182] Ma C., Feng K., Yang X., Yang Z., Wang Z., Shang Y., Fan G., Liu L., Yang S., Li X. (2021). Targeting macrophage liver X receptors by hydrogel-encapsulated T0901317 reduces atherosclerosis without effect on hepatic lipogenesis. Br. J. Pharmacol..

